# Cross-Clustering: A Partial Clustering Algorithm with Automatic Estimation of the Number of Clusters

**DOI:** 10.1371/journal.pone.0152333

**Published:** 2016-03-25

**Authors:** Paola Tellaroli, Marco Bazzi, Michele Donato, Alessandra R. Brazzale, Sorin Drăghici

**Affiliations:** 1 Department of Statistical Sciences, University of Padova, Padova, Italy; 2 Department of Computer Science, Wayne State University, Detroit, MI, United States of America; 3 Department of Obstetrics and Gynecology, Wayne State University School of Medicine, Detroit, MI, United States of America; University of Ulm, GERMANY

## Abstract

Four of the most common limitations of the many available clustering methods are: i) the lack of a proper strategy to deal with outliers; ii) the need for a good *a priori* estimate of the number of clusters to obtain reasonable results; iii) the lack of a method able to detect when partitioning of a specific data set is not appropriate; and iv) the dependence of the result on the initialization. Here we propose Cross-clustering (CC), a partial clustering algorithm that overcomes these four limitations by combining the principles of two well established hierarchical clustering algorithms: Ward’s minimum variance and Complete-linkage. We validated CC by comparing it with a number of existing clustering methods, including Ward’s and Complete-linkage. We show on both simulated and real datasets, that CC performs better than the other methods in terms of: the identification of the correct number of clusters, the identification of outliers, and the determination of real cluster memberships. We used CC to cluster samples in order to identify disease subtypes, and on gene profiles, in order to determine groups of genes with the same behavior. Results obtained on a non-biological dataset show that the method is general enough to be successfully used in such diverse applications. The algorithm has been implemented in the statistical language R and is freely available from the CRAN contributed packages repository.

## Introduction

Clustering is the process of partitioning elements into a number of groups (clusters) such that elements in the same cluster are more similar than elements in different clusters. Clustering has been applied in a wide variety of fields, ranging from medical sciences, economics, computer sciences, engineering, social sciences, to earth sciences [1, 2], reflecting its important role in scientific research. With several hundred clustering methods in existence [3], there is clearly no shortage of clustering algorithms but, at the same time, satisfactory answers to some basic questions are still to come.

Clustering methods are nowadays essential tools for the analysis of gene expression data, becoming routinely used in many research projects [4]. Many papers have shown that genes or proteins of similar function cluster together [5–10], and clustering methods have been used to solve many problems of biological nature. One of the most interesting of these problems is related to *disease subtyping*, i.e. the stratification of different patients in terms of underlying disease characteristics. This is extremely important in the drug development process, in which the correct identification of the subgroup of patients who are most likely to respond to the drug may be needed in order to get the drug approved by FDA. Also, ultimately, disease subtyping is expected to be the key for personalized therapies.

A widely used type of clustering is *K*-means [11–13], the best known squared error-based clustering algorithm [14]. This method consists in initializing a number of random centroids, one for each cluster, and then associating each element to the nearest centroid. This procedure is repeated until the locations of the centroids do not change anymore.

A similar clustering algorithm is Partition Around Medoids (PAM) [15], which intend to find a sequence of elements called *medoids* that are centrally located in clusters, with the goal to minimize the sum of the dissimilarities of all elements to their nearest medoid. Also Affinity Propagation [16] starts from a similar idea, identifying *exemplars* among data points and building clusters around these exemplars.

Another widely used clustering algorithm is Spectral clustering, which makes use of the eigenvalues of the similarity matrix of the data before clustering.

Many of the most widely used clustering methods, including *K*-means, PAM, and Spectral clustering, require the estimation of the most appropriate number of clusters for the data. Ideally, the resulting clusters should not only have good properties (compact, well-separated, and stable), but also give biologically meaningful results. This is an issue that derives from the more general problem of defining the term “cluster” [3] and has been extensively treated in the literature [17]. Furthermore, *K*-means is not a deterministic method, because the results are dependent on the initialization of the algorithm and can change between successive runs. The same not-deterministic property is shared by SOM [18], a neural network clustering method, which, even if it does not need the number of clusters to be defined *a priori*, requires the user to specify the maximum number of clusters. Another similar clustering algorithm we consider is AutoSOME [19], which, however, is also able to estimate the number of clusters.

Another very popular clustering methods category is *hierarchical clustering* (HC), where the term *hierarchical* refers to the relation between clusters, which are nested according to a pairwise distance matrix. Results obtained with HC methods are usually represented with a dendrogram, a tree diagram where the height of the vertical lines is proportional to the difference between each pair of elements or clusters. HC methods can be divided into *agglomerative* or *divisive* methods. Agglomerative clustering (also called *bottom up*) starts with each element belonging to one different cluster (singleton cluster). Following the initialization, a series of *merge* operations groups pairs of clusters based on a predefined distance metric, until only one cluster remains, containing all elements. Divisive clustering (also called *top down*) proceeds in the opposite way. At the beginning all elements belong to the same cluster and the algorithm successively divides it until all clusters are singleton clusters. Based on different definitions of the distance between two clusters, there are many agglomerative clustering algorithms. Two of the simplest and most popular methods include Complete-linkage (CL) [20] and Ward’s minimum variance criterion [21] henceforth referred to as “Ward”. The former uses the greatest among the distances of each pair of elements in a cluster to define the inter-cluster distance. The latter assumes that a cluster is represented by its centroid (the location that corresponds to the means of the coordinates in the multivariate space) and measures the proximity between two clusters in terms of the increase in the Sum of Squared Error (SSE) that results from merging two clusters.

One advantage of HC is that the number of clusters is not required as a parameter. However, this method does not solve this issue completely, as the number of clusters is not determined exactly. In HC the number of clusters can be chosen after the inspection of the dendrogram, by cutting it at a certain depth or choosing a number of clusters based on intuition (see Chap. 18 in [4]). As a disadvantage, HC has a great computational complexity of the order of *O*(*n*^2^) where *n* is the number of data points to be clustered [14].

The determination of the number of clusters is a common issue in many clustering methods [17, 22]. This problem is often solved by involving the use of indices that measure the quality of clusters according to their compactness and separation. However, a disadvantage shared by most of these indices is that they do not offer any indication whether the data should be clustered at all [22].

The requirement that the number of clusters has to be known in advance is only one of the most common limitations of clustering methods. Many existing methods will assign *all* the input data to some cluster. Even if there exist methods that allow for *overlapping* clustering (an element can simultaneously belong to more than one group), and *partial* clustering (not every element belongs to a cluster), the most widely used methods result in *complete* clustering, where every element is assigned to exactly one cluster, disregarding the possibility that some elements might be *outliers* that do not belong to any real group. There are various applications for which it makes little or no sense to force all data items to belong to some group, and doing so inevitably leads to poorly-coherent clusters. For example, genetic data often contain a significant amount of noise, and complete clustering leads to the presence of many outliers among the genetic entities that are clustered.

In this work we present the Cross-Clustering (CC) algorithm, a technique that combines two well-established bottom-up HC algorithms in order to identify the correct number of clusters with little input by the user, while also detecting and removing outliers that could undermine the quality of the clusters obtained.

## Methods

The CC algorithm proposed here overcomes four of the most common limitations of the existing clustering algorithms. First, it does not necessarily group all the elements into clusters, thus falling in the category of partial clustering methods. The basic assumption made in this algorithm is that the data points that greatly deteriorate the quality of the clusters represent noise, and thus should not be included in the final clustering. Second, the algorithm automatically identifies the optimal number of clusters. Third, CC allows the possibility of obtaining one cluster as result, suggesting that partitioning that specific data may not be appropriate. Fourth, CC is a deterministic algorithm that does not depend on any initialization, and always produces reproducible results. These results are obtained by combining the two basic principles of Ward and CL. In principle, we could consider all the possible combinations of clustering methods in order to find the combination that works best, but we decided to combine these two algorithms for two intuitive reasons: i) as Ward attempts to minimize the sum of the squared distances of points from their cluster centroids, it is able to build well-separated clusters and to provide a good estimate of the number of clusters; ii) as CL defines the proximity of two clusters as the maximum of the distance between any two points in the two different clusters, it is not optimal in separating clusters appropriately, but it is able to clearly identify outliers.

The parameters required by CC are optional and include a reasonable interval of values for the number of clusters for Ward (*n*_*W*_), and a maximum number of clusters to be used in CL (*n*_*C*_). Partitions are computed for both methods for each value in the input range. Then, each partition obtained cutting the Ward dendrogram in correspondence with *n*_*W*_ clusters is combined with each partition resulting from the cutting of CL with a number of clusters *n*_*C*_, which has to be higher than *n*_*W*_, allowing for the identification of small and singleton clusters, likely to contain outliers. The algorithm then choses the partitioning yielding the maximum consensus between the two methods, providing the number of clusters and the elements to be considered as outliers. Note that *n*_*W*_ and *n*_*C*_ are there just to reduce the computational effort. In principle, the cluster range can always be set from a minimum of 2 clusters to a maximum number of clusters equal to the number of data points.

### The algorithm

As explained above, CC can use two optional parameters in order to reduce the computational complexity. The first one is the range for the number of clusters $I^{W}\;=\;[n_{W_{min}},\ldots ,n_{W_{max}}]$. This range can be chosen by performing Ward and choosing a reasonable interval after the visual inspection of the results though the dendrogram. A set of different partitions $k_{i}^{W}$, where *i* = 1, … , length(I^W^), is obtained by cutting the tree at the levels associated to each *n*_*W*_*i*__ ∈ *I*^*W*^. The second parameter is the range for the number of clusters in CL, $I^{C}\;=\;[n_{W_{min}}+1,\ldots ,n_{W_{max}}]$, where *n*_*C*_*max*__ is an arbitrary number, greater than *n*_*W*_*max*__, but smaller than the number of elements minus one. Also this range can be chosen after the visual inspection of the CL dendrogram. Setting a high *n*_*C*_*max*__ allows to isolate those elements which are less likely to belong to a cluster, eliminating them from the final partition. Then, the CC algorithm works as follows:

1. A set of different partitions $k_{j}^{C}$, where *j* = 1, …, length(I^C^), is obtained by cutting the tree at the levels associated to each *n*_*C*_*j*__ ∈ *I*^*C*^.
2. For all the possible pairs $k_{j}^{C}$,$k_{i}^{W}$ such that *n*_*C*_*j*__ > *n*_*W*_*i*__ build the contingency table *A*, where the element *a*_*rs*_ represents the number of elements belonging simultaneously to cluster $c_{r}^{W}$ (the *r*-th cluster obtained with Ward when the number of total clusters is set at $n_{i}^{W}$) and to cluster $c_{s}^{C}$ (the *s*-th cluster obtained with CL when the number of total clusters is set at $n_{j}^{C}$). The requirement *n*_*C*_*j*__ > *n*_*W*_*i*__ if essential in order to isolate as much noise and singleton elements as possible.
3. Permute the columns of matrix *A* until the sum of elements on the diagonal of *A*, denoted as sum(diag(A)), is the largest possible. The term max(sum(diag(A))) represents the maximum amount of overlap between partitions $k_{i}^{W}$ and $k_{j}^{C}$, and it will be denoted henceforth as *MO*_*ij*_.
4. Choose the pair(i*,j*) = argmax_i,j_MO(i,j). The optimal number of clusters is indicated by $n_{W_{i}}^{*}$, and this follows from the ability of Ward to identify well-separated clusters. The optimal partitioning is identified by the consensus clusters obtained by combining $k_{j*}^{C}$ and $k_{i*}^{W}$.

For discussion on special cases and for an example refer to the Supporting Information.

## Results

In order to assess the performance of CC, we compared it on simulated data with the methods on which it is based, as well as with a method that attempts to find the correct number of clusters and to identify outliers, the DBSCAN method [23], and with PAM, Affinity propagation, autoSOME, and Spectral clustering. Furthermore, we also compared CC with Ward, CL, DBSCAN, *K*-means and SOM on three real datasets, showing that CC is able to obtain meaningful results that represent the underlying partitioning of the data. Finally, we compared CC with two approaches for cancer subtyping, non-Negative Matrix Factorization (NMF) [24] and SPARCoC [25], applying the algorithms on the data from the paper where SPARCoC was first introduced.

### Simulation study

Our simulated study was set up as follows: we generated 100 simulated datasets, each describing the expression profiles of 2,000 genes for 5 samples. The expression profile of each gene was chosen among five distinct *behaviors* over the samples: constant at a positive value (500 genes), increasing (250 genes), decreasing (700 genes), oscillating (300 genes), and convex (100 genes). We added random noise from N(μ=0,σ=0.2), N(μ=0,σ=0.5), N(μ=0,σ=1), N(μ=0,σ=1.5) to each of the 100 datasets, resulting in 4 groups of 100 datasets. An additional set of 150 gene profiles were drawn from a Uniform distribution between 0 and the maximum simulated value present in the dataset, and they were added to each of the 400 datasets. We performed principal component analysis (PCA) on the data, and plotted the first three principal components (cumulative percentage of variance explained 89.8%). Each gene in S1 Fig in S1 File has been plotted as a point in the three-dimensional space corresponding to the first three principal components, denoted with PC1, PC2, and PC3, and it shows how categories are well defined even when *σ* = 1.5, while the outlier genes are distributed uniformly around the center.

We applied the Ward and CL algorithms to the data, using the Euclidean distance. The number of clusters *k* is chosen as follows. We computed the clusterings for each *k* in the interval [2, 20] and we used the value of *k* for which the Average Silhouette Width (ASW) was maximum. This represents a reasonably good choice for *k*, as the Silhouette Width is defined as the average of the degree of confidence of an element to be in a cluster. The Silhouette Width lies in [−1, 1] and should be maximized (more details about the Silhouette Width can be found in the Supporting Information). Intuitively, by using the maximum of the average Silhouette Width we are choosing in an unsupervised way the partition with the best quality of the results. The only parameters to be set in CC are the interval for the number of clusters in Ward, in this case set to *k* ∈ [2: 19], and the maximum number of clusters in CL, which is set to 20. These parameters have been used in all the applications of CC throughout this work.

DBSCAN determines automatically the number of clusters and classifies points in low-density regions as noise. The issue with this algorithm is that it needs the user to determine two parameters, *ϵ* and *MinPts*. A point is a *core point*, i. e. is in the interior of a cluster, if it has more than a specified number of points (*MinPts*) within a radius of *ϵ*, the reachability index (a sort of radius essential in order to compute the density). The criterion suggested by the authors [23] for the choice of the parameters only works for two-dimensional datasets. The idea is that points in a cluster are roughly at same distance from their *n*^*th*^ nearest neighbor, while the distance from noise points is higher. The suggested *n* for two-dimensional data is 4. Therefore, plotting the sorted distances, sorted in descending order, of each point from its *n*^*th*^ nearest neighbor gives hints concerning the proximity of the elements in the data. A threshold point *p* should be chosen to be the first one in the first “valley” of the sorted distances: all the points on the left of the threshold are considered to be noise, while all other points are assigned to some cluster. The parameters are then set such as *ϵ* = *dist*(*p*) and *MinPts* = *n*. As it is not practical to use this criterion on 100 simulations, here we set *ϵ* ∈ [0.1, 10] and *MinPts* equal 5, the default value of the fpc R package [26].

The adjusted Rand Index (ARI) [27], an updated form of the Rand Index [28], measures the agreement between two partitionings correcting it for chance agreement. This index has an expected value of 0 for independent partitionings and maximal value 1 for identical clusterings. Negative values are possible and indicate less agreement than expected by chance. There are several external indices like the ARI in the literature, such as Hubbert [29] and Jaccard [30], but they can be sensitive to the number of classes in the partitions or to the distributions of elements in the cluster [31]. The ARI is not affected by any of these issues [32] and has been found to have the most desirable properties in a comparative study of several pairwise clustering agreement criteria [33], making it the choice as main measure of comparison. In this work, we used the ARI to compare the partitionings obtained with different clustering methods with the reality, in order to measure the quality of the prediction. However, as the ARI compares only partitions of the same length, elements identified to be outliers by CC have been considered as a single cluster. For computing the ARIs we used the R package clues [34]. S2 Fig in S1 File shows that CC had a good performance when compared with the methods that constitute it, resulting in values of ARI always higher than these coming from CL and very similar to Ward results. S3 Fig in S1 File shows a very similar performance also when the chosen distance is Chebychev.

An issue in using the ARI to score different results is that we are considering the 150 outlier profiles as a single cluster, while they could represent 150 different clusters. Therefore, we analyzed the ability of various methods to cluster elements correctly. Assuming that we are analyzing a dataset with a specific method M that produces a partitioning of the genes into clusters. Given a *real* cluster C, assume that the majority of elements of C is clustered together by M in a cluster X. We then identify X as being representative of C. Given these assumptions, we can define:

- True Positives: genes belonging to cluster C clustered together by M in the cluster with the largest number of genes actually coming from cluster C.
- False Positives: genes not belonging to cluster C clustered together by M in the cluster with the largest number of genes actually coming from cluster C.
- False Negatives: genes belonging to cluster C not clustered by M in the cluster with the largest number of genes actually coming from cluster C.
- True Negatives: genes not belonging to cluster C not clustered by M in the cluster with the largest number of genes actually coming from cluster C.

Once these categories are defined, we can plot, for each method, the Receiver Operating Characteristic (ROC) curve, to study their ability to identify correct cluster memberships. The measure used to summarize the performance is the Area Under the Curve (AUC). The AUC combines sensitivity and specificity, where sensitivity measures the proportion of actual positives that are correctly identified as such, while specificity measures the proportion of negatives that are correctly identified as such. AUC for each of the *K* = 6 clusters (considering the outliers as a single cluster) has been computed with the R package pROC [35]. Also the sensitivity, the Positive Predictive Value (PPV), and geometrical accuracy (Acc_g) have been computed.

S4–S7 Figs in S1 File show boxplots of the AUC for each of the six clusters over 100 simulations, with a varying *σ* in the added noise, for CC, CL, and Ward. The last group represents the outliers. S8–S11 Figs in S1 File show boxplots of the average AUC over the six clusters for 100 simulations, for various values of *ϵ*. The comparisons show that CC obtains an higher AUC in every cluster and with each *σ* with respect to the methods that constitute it, proving its ability in detecting the correct clusters and in identifying the outliers. S12–S15 Figs in S1 File show that CC obtains higher AUCs for each cluster with respect to Ward and CL also when the distance metric chosen is Chebychev. The comparison with DBSCAN shows a high variability of this method in the resulting AUC, showing its sensitivity to parameter choice and to *σ*, with performance decreasing as *σ* increases. S16–S51 Figs in S1 File show boxplots of the sensitivity, PPV, and geometrical accuracy for Ward, CL, and K-means methods, showing that CC performs generally better. We also compared the quality of the results in terms of *entropy*. The entropy is a common way to measure the level of *impurity* (confusion) in a group: the higher it is, the more the information content. The *information gain* can be used as a quality scoring of clustering results, as it estimates the amount estimates the amount of information gained by clustering data as measured by the reduction in class entropy [36] (the higher, the better). We computed the information gain of Ward, CL and CC for each number of clusters using the R package FSelector [37]. Results are shown through boxplots for *σ* = 0.5 in S128–S130 Figs in S1 File, and in terms of average information gain in S4 Table in S1 File. This analysis proves that CC always led to the highest information gain and to the lower standard deviation, meaning that the quality of the clusters in terms of purity is always higher when using CC. We also compared CC to Affinity Propagation, autoSOME, Partitioning Around Medoids (PAM), and Spectral clustering. Results are shown in S52–S103 Figs in S1 File, where the superiority of CC in terms of ARI, sensitivity, PPV, and geometrical accuracy is proven. Please, note that the comparisons are performed on 100 simulations for each method with each of the four different added variability, every time choosing the number of clusters in an unsupervised way.

We compared the ability of CC to detect the correct number of clusters. In order to do so, we compared the results of CC with several well established methods: CH [38], Silhouette Width [39], Dunn Index [40], Beale Index [41], C-Index [42], Duda Index [43], H [1], KL [44], Gap [45], and Jump [46]. A good summary of these methods is given by [22], while [17] conducted a simulation study of the performance of 30 decision rules. These methods were combined with Ward, CL, and *K*-means. The results of such comparisons are reported in S104–S115 Figs in S1 File and in S5 Table in S1 File, and show that CC always detected the correct number of clusters, while the other methods showed variability in the results.

Lastly, we wanted to assess how stable the method is with regard to the input parameters ($n_{W_{min}}$, $n_{W_{max}}$, and $n_{C_{max}}$). Therefore, we performed CC with 10 different pairs of values for the boundaries of *I*^*W*^, while setting a high $n_{C_{max}}=99$ on the simulated data after the removal of outliers (a representation of the intervals can be seen in S116 Fig in S1 File). The results, in terms of number of clusters identified by CC, are shown in S117–S120 Figs in S1 File and prove the robustness of the method, which is always able to identify the correct number of clusters (*K* = 5) also with *I*^*W*^ very large or asymmetric and independently from *σ*.

### Application on real data

#### Brain tumors dataset

Central nervous system embryonal tumors (CNSET) are a group of tumors characterized by high heterogeneity. The understanding of the biological mechanisms underlying CNSET is still limited [47]. Although the classification of these tumors based on histopathological appearance is still debated, they are usually divided in: medulloblastoma (MD), CNS primitive neuroectodermal tumors (PNET), rhabdoid tumors (Rhab), and malignant glioma (Mglio). The public dataset used by [47] contains 5,299 gene expression profiles of 42 samples: 10 MD, 10 Rhab, 8 PNET, 10 Mglio, and 4 normal human cerebella (Ncer). One of the particular aspects of this case study is that we know the ground truth, i.e. we know the underlying clusters. The dataset has been log_2_ transformed and scaled to mean zero and variance one.

Similarly to the analysis performed on simulated data, we used the ASW in order to choose the number of clusters and we compared the results obtained by applying CC, Ward, CL, DBSCAN, *K*-means, and SOM on the brain tumors dataset. Furthermore, we performed the Fisher exact test for count data in order to test if any cluster was over-represented in any particular subtype. All the p-values obtained were corrected for multiple testing with the BH method [48].

**Fig 1:**
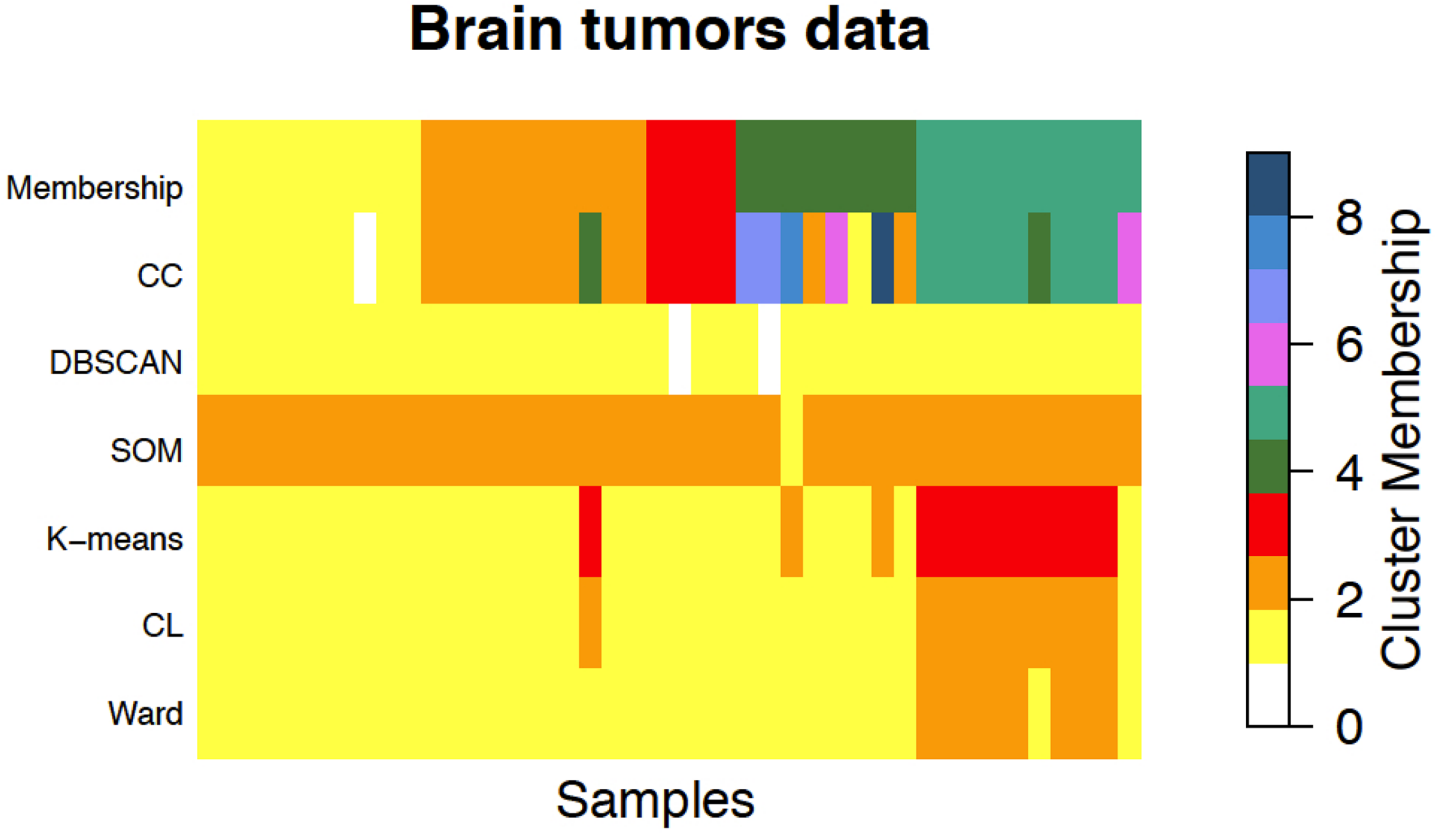
Graphical representation of the true membership (first row) of the 42 samples in brain tumors data, compared with the memberships resulting from CC, DBSCAN, SOM, K-means, CL, and Ward. The five subtypes in the order of the first row of the image are: medulloblastoma (MD), malignant gliomas (MGlio), normal human cerebella (Ncer), primitive neuroectodermal tumors (PNET), and atypical teratoid/rhabdoid tumors (Rhab). The colors represent the index of the cluster given by each method. The white color represents outliers, only detected by CC and DBSCAN. Classical approaches performed poorly, obtaining ARI values ranging from 0.003 to 0.19, the highest value being obtained by *K*-means. In terms of number of clusters, the ASW criterion for Ward, CL, and SOM identified two clusters (maximum ASW of 0.19 in each method), while *K*-means resulted in three clusters (maximum ASW of 0.17). In contrast, CC obtained an ARI of 0.64, identifying nine clusters and one sample as an outlier. Although CC identified more than five clusters, four of them almost perfectly represented four of the real subtypes while PNET, a subtype known to present heterogeneous histological characteristics, was fragmented in six clusters.

We applied Ward and CL to the data using the same parameters used for the simulation study. In order to address the dependence of *K*-means to the initialization of the parameters, we ran the algorithm 10 times, every time choosing the *k* in the interval [2, 20] that maximizes the ASW. We applied SOM with a rectangular topology and all the default parameters of the R package kohonen ( [49]—see the Supporting Information). In order to choose the dimensions of the grid, we applied SOM with 70 combinations of dimensions (see S6 Table in S1 File) and we computed the ASW for each combination. Classical approaches performed poorly, obtaining ARI values ranging from 0.003 to 0.19, the highest value being obtained by *K*-means. In terms of number of clusters, the ASW criterion for Ward, CL, and SOM identified two clusters (maximum ASW of 0.19 in each method), while *K*-means resulted in three clusters (maximum ASW of 0.17). Among the two clusters identified by Ward, one cluster represented the subtype Rhab (p-value of 3.81*e*^−06^). CL reported two clusters, one of which represented the Rhab subtype (p-value of 0.0003). *K*-means reported three clusters, one of which represented the PNET subtype (p-value of 0.0005). None of the clusters reported by SOM was over-represented in any subtype, as expected from the low ARI. In contrast, CC obtained an ARI of 0.64, identifying nine clusters and one sample as an outlier. Although CC identified more than five clusters, four of them almost perfectly represented MD, MGlio, Ncer, and Rhab subtypes (p-values, respectively, of 9.53*e*^−06^, 1.94*e*^−05^, 1.12*e*^−04^, 9.53*e*^−06^), as it is shown in Fig 1. The PNET subtype was represented by six of CC clusters. This particular subtype is defined by the World Health Organization (WHO) with useful guidelines for diagnosis its heterogeneous histological characteristics and malignancy grade [50]. All the contingency tables reporting both the real classification in subtypes and the clusters identified by each method are shown in S7–S11 Tables in S1 File. A summarization of the results can be found in S12 Table in S1 File, while dendrograms for the HC methods can be seen in S121–S122 Figs in S1 File.

Lastly, we applied DBSCAN trying different types of *n*-th nearest neighbors. Setting the value of *ϵ* and *MinPts* to each of the pairs obtained for different values of *n* (*ϵ* = 25.50, *MinPts* = 4;*ϵ* = 27.41, *MinPts* = 5;*ϵ* = 27.12, *MinPts* = 6;*ϵ* = 27.65, *MinPts* = 7) always led to a unique non-noise cluster (2 outliers and 40 elements grouped in a single cluster).

#### Breast cancer dataset

More than 1.7 million new cases of breast cancer occurred among women worldwide in 2012 [51], making breast cancer the most common cancer in women worldwide. The incidence of breast cancer in women in 2011 (most recent data available) was of 124.3 per 100,000, while the mortality was of 21.5 per 100,000 [52]. Increasing evidence suggests that breast cancer can be classified in multiple subtypes based on the kind of treatment, level of aggressivity, risk factors, and survival rates. Depending on the number of biological markers (proteins associated with mechanisms underlying the disease), most studies divide breast cancer into four major molecular subtypes: luminal A, luminal B, triple negative/basal-like (TN), and HER2 over-expression (approximate prevalences of, respectively, 40%, 20%, 20%, and 15%). The remaining cases are less common and often listed as unclassified.

The public dataset GSE38888 from the Gene Expression Omnibus database [53] describes the expression profiles of 719,690 probesets and 30 samples, classified in two subtypes: 16 luminal and 14 TN [54]. Similarly to the brain tumor dataset, we compared the results obtained by applying CC, Ward, CL, DBSCAN, K- means, and SOM.

**Fig 2:**
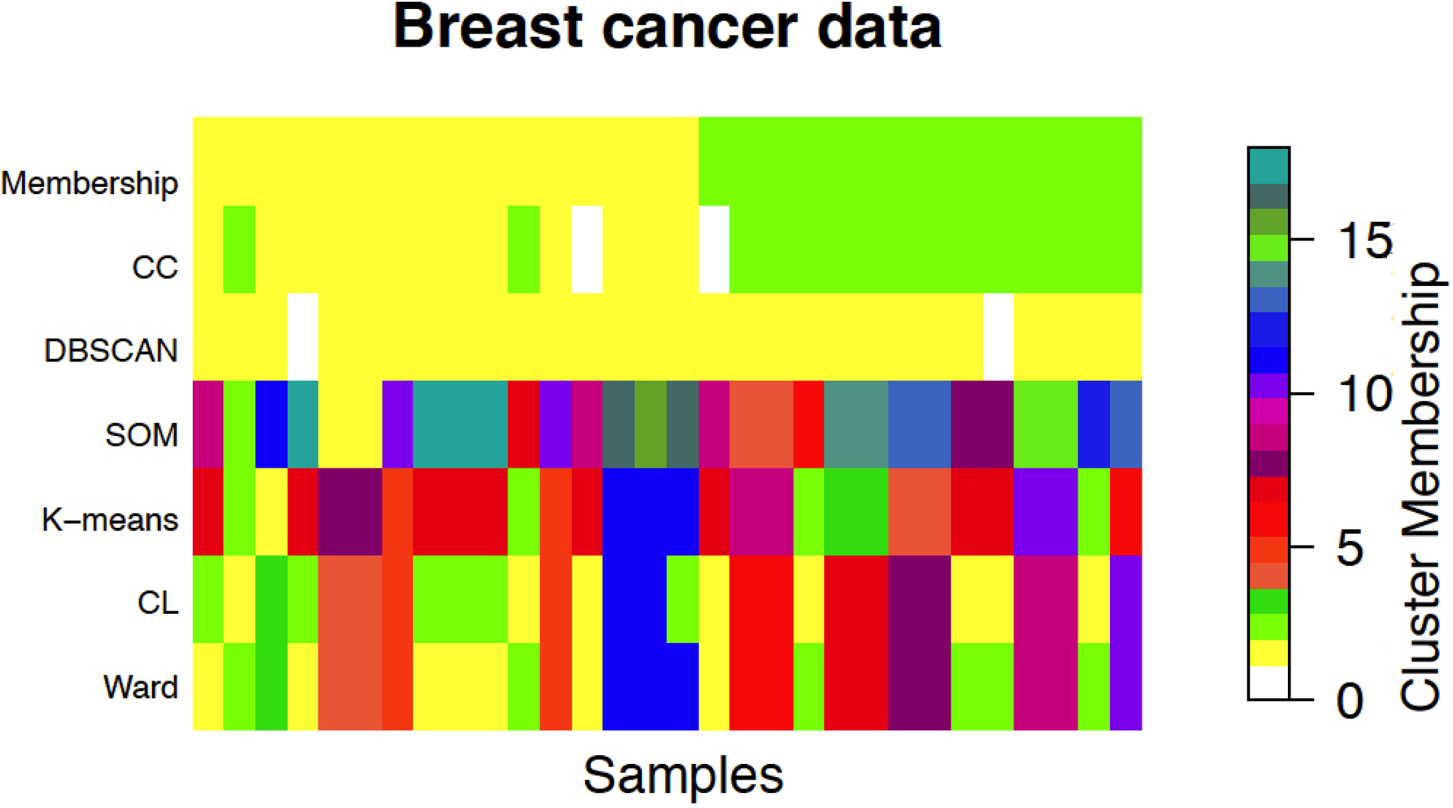
Graphical representation of the true membership (first row) of the 30 samples in breast cancer data, compared with the memberships resulting from Cross-clustering (CC), DBSCAN, SOM, K-means, Complete-linkage (CL), and Ward. There are two subtypes: luminal and triple negative (TN). The yellow color represents the luminal subtype, the green color represents the TN subtype, while white color represents outliers, only detected by CC and DBSCAN. Different colors represent only the index of the cluster given by each method. Classical approaches performed poorly, obtaining ARI values ranging from 0.04 to 0.1, the highest value being obtained by CL. In terms of number of clusters, the ASW criterion for Ward, CL, and *K*-means identified 11 clusters (maximum ASW of 0.24, 0.24, and 0.25 respectively), while SOM resulted in 18 clusters (maximum ASW = 0.18), out of which two were empty. DBSCAN detected one cluster containing 28 of the 30 elements. In contrast, CC obtained an ARI of 0.63, showing great agreement with the ground truth, and identifying correctly the number of clusters. Two out of the 30 elements were considered outliers. Furthermore, it is important to notice that, while CC requires a loose set of parameters (a range where the real number of clusters has to be found), *K*-means require the correct number of clusters, to be found with one of the many techniques available, and SOM requires two parameters whose choice is not easy.

Classical approaches performed poorly, obtaining ARI values ranging from 0.04 to 0.1, the highest value being obtained by CL. In terms of number of clusters, the ASW criterion for Ward, CL, and *K*-means identified 11 clusters (maximum ASW of 0.24, 0.24, and 0.25 respectively), while SOM resulted in 18 clusters (maximum ASW of 0.18), out of which two were empty. DBSCAN detected one cluster containing 28 of the 30 elements. In contrast, CC obtained an ARI of 0.63, showing great agreement with the ground truth, and identifying correctly the number of clusters. Two out of the 30 elements were considered outliers. A graphical representation of the results is shown in Fig 2, while a summarization of the results can be found in S13 Table in S1 File, and dendrograms for the HC methods can be seen in S123 and S124 Figs in S1 File.

CC was the only method to yield clusters that were over-represented in elements belonging to real subtypes. The two clusters identified by CC represented the two cancer subtypes, luminal with a p-value of 2.81*e*^−05^, TN with a p-value of 3.28*e*^−05^. All the contingency tables reporting both the real classification in subtypes and the clusters identified by each method are reported in S14–S18 Tables in S1 File.

#### Olive oil dataset

Finally, in order to prove that CC is general enough to be successfully used for clustering a wide range of data, we clustered the real, public, and not biological oliveoil data set in the pdfCluster R package. This data set contains eight measurements on 572 different specimen of olive oil produced in various regions in Italy: northern Apulia, southern Apulia, Calabria, Sicily, inland Sardinia, coast Sardinia, eastern and western Liguria, Umbria. The data set is used to evaluate the ability of the clustering methods of reconstructing the region of origin of the olive oils.

CC outperformed the other methods, obtaining an ARI of 0.60. The other methods obtained sensibly lower values: Ward’s 0.29, Complete-linkage 0.31, K-means 0.29, and SOM 0.30. A graphical representation of the results is shown in S125 Fig in S1 File, where the colors represent the index of the cluster given by each method. The white color represents outliers, only detected by CC and DBSCAN. CC detected three clusters and an outliers cluster, while other methods always detected only two clusters, with the exception of DBSCAN that identified only one cluster and a few outliers. As can be seen in S19 to S24 Tables in S1 File, identifying more than two clusters allowed to better represent the true classification.

#### Other subtyping methods

In order to compare CC with the two well-known subtyping methods SPARCoC [25] and NMF [24] we applied our method on two sub-datasets of the Jacob dataset (GSE68465 from the Gene Expression Omnibus database) on which SPARCoC has been validated and compared to NMF. Data were *log*_2_ transformed and scaled to mean zero and variance one, then it was divided in two distinct sub-datasets TM and HM (as done in the original SPARCoC paper). Kaplan-Meier plots (S126–S127 Figs in S1 File) show statistically significant differences in 5-year overall survival between the two clusters of patients for each dataset (p-values: *p* = 0.0426 for TM and *p* = 0.0002 for HM by log-rank test). In the original paper, SPARCoC proved to be more robust than NMF, achieving a p-value of 0.0032 for TM and *p* = 0.0106 for HM. These results show that CC is able to correctly separate the two groups, correctly determining the underlying biological differences among them. These results are comparable with SPARCoC and NMF over the two sub-datasets.

## Discussion

The CC algorithm provides an intuitive and easily implementable approach for clustering of gene expression data. CC presents several advantages over existing methods: i) it does not require *a priori* knowledge on the number of clusters, ii) it leaves outlier elements unassigned, and iii) it can yield only one cluster as result, suggesting that data should not be clustered at all. Furthermore, even though in this paper we only report results obtained on biological data, CC can be successfully applied on any type of data, as demonstrated by the results obtained on the simulated data. The only (unavoidable) weakness of CC is its quadratic computational complexity, inherited from the methods that constitute it. The expected time complexity of Ward’s method is upper-bounded by *O*(*n* log *n*) [55], while the worse case time complexity of the Complete-linkage clustering is *O*(*n*^2^log *n*) [56], where *n* is the number of data points to be clustered. Defining with *α* the cardinality of the interval for the number of clusters *I*^*W*^ for Ward’s algorithm, and with *β* the cardinality of the interval for the number of clusters *I*^*C*^ for Complete-linkage; in the worst case launching CC we are running *αβ* algorithms. Then, the maximum computational complexity of CC is equal to *O*(*n*^2^ *αβ* log *n*). Also in terms of running time CC resulted to be competitive with the other clustering and subtyping methods, as can be seen in S25 Table in S1 File.

We compared CC with the most widely used clustering methods and CC consistently obtained partitions closer to the reality than the results obtained with these methods. As the distance is a key element in cluster analysis, we performed our comparisons using both Euclidean and Chebychev distances in order to check how sensible results are to the choice of the distance, and in both cases CC performed better than the methods it was compared with. Furthermore, CC proved to be an useful tool for detecting a suitable number of clusters in the data, better of most of the well established criteria proposed in the literature. When compared to DBSCAN, CC showed a good performance and more robustness, while DBSCAN was highly sensible to parameters choice and data variability. Furthermore, in DBSCAN the only available approach to parameters choice works only for two dimensional datasets. When applied on three real publicly available datasets, CC was able to identify subtypes better than the other approaches. *K*-means and SOM produced different results in different runs as the two methods are strictly dependent on the initialization of the algorithm, yielding results ranging from very good to very poor. In contrast, the results of CC were stable, as it does not involve any random initialization. Finally, when applying CC, NMF and SPARCoC on the same two sub-datasets, we obtained similar results in terms of ability to recognize cohorts with different survivals.

## Supporting Information

S1 File Contains supporting Figures S1–S130 and Tables S1–S25. (PDF)
