# Supplementary material for: Cross-Clustering: A Partial Clustering Algorithm with Automatic Estimation of the Number of Clusters
Source: PLoS One. 2016 Mar 25;11(3):e0152333. doi: 10.1371/journal.pone.0152333 (PMC4807765; doi:10.1371/journal.pone.0152333)
Supplement: S1 File — (PDF) [file pone.0152333.s001.pdf]

Cross-clustering: a partial clustering algorithm with automatic estimation of the number of clusters - Submission to PLOS Journals - Supporting Information

Paola Tellaroli [1*], Marco Bazzi[1], Michele Donato [2], Alessandra R. Brazzale [1], Sorin Drăghici [2,3]

1 Department of Statistical Sciences, University of Padova, Padova, Italy
2 Department of Computer Science, Wayne State University, Detroit, MI, 48202, U.S.A.
3 Department of Obstetrics and Gynecology, Wayne State University School of Medicine, Detroit, MI, 48201, U.S.A.

List of Figures

S1 Principal Component Analysis plot of one simulation of the data with the highest variance on the first three principal components. 8
S2 Boxplots of the Adjusted Rand Index resulting from applying CC, CL, and Ward's algorithm on simulated data. 9
S3 Boxplots of the Adjusted Rand Index resulting from applying CC, CL, and Ward's algorithm on simulated data with Chebychev distance. . . 10
S4 Boxplots of the Area Under the Curve in each simulated clusters with CC, CL, and Ward algorithm when $\sigma = 0.2$. 11
S5 Boxplots of the Area Under the Curve in each simulated clusters with CC, CL, and Ward algorithm when $\sigma = 0.5$. 11
S6 Boxplots of the Area Under the Curve in each simulated clusters with CC, CL, and Ward algorithm when $\sigma = 1$. 12
S7 Boxplots of the Area Under the Curve in each simulated clusters with CC, CL, and Ward algorithm when $\sigma = 1.5$. 12
S8 Boxplots of the average Area Under the Curve over the simulated clusters coming from CC and DBSCAN when $\sigma = 0.2$. 13
S9 Boxplots of the average Area Under the Curve over the simulated clusters coming from CC and DBSCAN when $\sigma = 0.5$. 13
S10 Boxplots of the average Area Under the Curve over the simulated clusters coming from CC and DBSCAN when $\sigma = 1$. 14
S11 Boxplots of the average Area Under the Curve over the simulated clusters coming from CC and DBSCAN when $\sigma = 1.5$. 15
S12 Boxplots of the Area Under the Curve in each simulated clusters with CC, CL, and Ward algorithm when $\sigma = 0.2$ using the Chebychev distance. 15
S13 Boxplots of the Area Under the Curve in each simulated clusters with CC, CL, and Ward algorithm when $\sigma = 0.5$ using the Chebychev distance. 16
S14 Boxplots of the Area Under the Curve in each simulated clusters with CC, CL, and Ward algorithm when $\sigma = 1$ using the Chebychev distance. 17
S15 Boxplots of the Area Under the Curve in each simulated clusters with CC, CL, and Ward algorithm when $\sigma = 1.5$ using the Chebychev distance. 18
S16 Boxplots of the sensitivity with CC and Ward when $\sigma = 0.2$. 19

S17 Boxplots of the sensitivity with CC and Ward when $\sigma = 0.5$. 19
S18 Boxplots of the sensitivity with CC and Ward when $\sigma = 1$. 20
S19 Boxplots of the sensitivity with CC and Ward when $\sigma = 1.5$. 20
S20 Boxplots of the PPV with CC and Ward when $\sigma = 0.2$ 21
S21 Boxplots of the PPV with CC and Ward when $\sigma = 0.5$ 21
S22 Boxplots of the PPV with CC and Ward when $\sigma = 1$ 22
S23 Boxplots of the PPV with CC and Ward when $\sigma = 1.5$ 22
S24 Boxplots of the Acc_g with CC and Ward when $\sigma = 0.2$ 23
S25 Boxplots of the Acc_g with CC and Ward when $\sigma = 0.5$ 23
S26 Boxplots of the Acc_g with CC and Ward when $\sigma = 1$ 24
S27 Boxplots of the Acc_g with CC and Ward when $\sigma = 1.5$ 24
S28 Boxplots of the sensitivity with CC and CL when $\sigma = 0.2$. 25
S29 Boxplots of the sensitivity with CC and CL when $\sigma = 0.5$. 25
S30 Boxplots of the sensitivity with CC and CL when $\sigma = 1$. 26
S31 Boxplots of the sensitivity with CC and CL when $\sigma = 1.5$. 26
S32 Boxplots of the PPV with CC and CL when $\sigma = 0.2$. 27
S33 Boxplots of the PPV with CC and CL when $\sigma = 0.5$. 27
S34 Boxplots of the PPV with CC and CL when $\sigma = 1$. 28
S35 Boxplots of the PPV with CC and CL when $\sigma = 1.5$. 28
S36 Boxplots of the Acc_g with CC and CL when $\sigma = 0.2$. 29
S37 Boxplots of the Acc_g with CC and CL when $\sigma = 0.5$. 29
S38 Boxplots of the Acc_g with CC and CL when $\sigma = 1$. 30
S39 Boxplots of the Acc_g with CC and CL when $\sigma = 1.5$. 30
S40 Boxplots of the sensitivity with CC and K-means when $\sigma = 0.2$. 31
S41 Boxplots of the sensitivity with CC and K-means when $\sigma = 0.5$. 31
S42 Boxplots of the sensitivity with CC and K-means when $\sigma = 1$. 32
S43 Boxplots of the sensitivity with CC and K-means when $\sigma = 1.5$. 32
S44 Boxplots of the PPV with CC and K-means when $\sigma = 0.2$. 33
S45 Boxplots of the PPV with CC and K-means when $\sigma = 0.5$. 33
S46 Boxplots of the PPV with CC and K-means when $\sigma = 1$. 34
S47 Boxplots of the PPV with CC and K-means when $\sigma = 1.5$. 34
S48 Boxplots of the Acc_g with CC and K-means when $\sigma = 0.2$. 35
S49 Boxplots of the Acc_g with CC and K-means when $\sigma = 0.5$. 35
S50 Boxplots of the Acc_g with CC and K-means when $\sigma = 1$. 36
S51 Boxplots of the Acc_g with CC and K-means when $\sigma = 1.5$. 36
S52 Boxplots of the ARI resulting from CC and autoSOME on simulated data. 38
S53 Boxplots of the sensitivity with CC and autoSOME when $\sigma = 0.2$. . . . 38
S54 Boxplots of the sensitivity with CC and autoSOME when $\sigma = 0.5$. . . . 39
S55 Boxplots of the sensitivity with CC and autoSOME when $\sigma = 1$. 39
S56 Boxplots of the sensitivity with CC and autoSOME when $\sigma = 1.5$. . . . 40
S57 Boxplots of the PPV with CC and autoSOME when $\sigma = 0.2$. 40
S58 Boxplots of the PPV with CC and autoSOME when $\sigma = 0.5$. 41
S59 Boxplots of the PPV with CC and autoSOME when $\sigma = 1$. 41
S60 Boxplots of the PPV with CC and autoSOME when $\sigma = 1.5$. 42
S61 Boxplots of the geometric accuracy with CC and autoSOME when $\sigma = 0.2$ 42
S62 Boxplots of the geometric accuracy with CC and autoSOME when $\sigma = 0.5$ 43
S63 Boxplots of the geometric accuracy with CC and autoSOME when $\sigma = 1$ 43
S64 Boxplots of the geometric accuracy with CC and autoSOME when $\sigma = 1.5$ 44
S65 Boxplots of the ARI resulting from CC and Affinity Propagation on simulated data. 45
S66 Boxplots of the sensitivity with CC and Affinity Propagation when $\sigma = 0.2$ 45
S67 Boxplots of the sensitivity with CC and Affinity Propagation when $\sigma = 0.5$ 46

S68 Boxplots of the sensitivity with CC and Affinity Propagation when $\sigma = 1$ 46
S69 Boxplots of the sensitivity with CC and Affinity Propagation when $\sigma = 1.5$ 47
S70 Boxplots of the PPV with CC and Affinity Propagation when $\sigma = 0.2$. 48
S71 Boxplots of the PPV with CC and Affinity Propagation when $\sigma = 0.5$. 48
S72 Boxplots of the PPV with CC and Affinity Propagation when $\sigma = 1$ . . 49
S73 Boxplots of the PPVwith CC and Affinity Propagation when $\sigma = 1.5$ . . 49
S74 Boxplots of the geometric accuracy with CC and Affinity Propagation when $\sigma = 0.2$ 50
S75 Boxplots of the geometric accuracy with CC and Affinity Propagation when $\sigma = 0.5$ 50
S76 Boxplots of the geometric accuracy with CC and Affinity Propagation when $\sigma = 1$ 51
S77 Boxplots of the geometric accuracy with CC and Affinity Propagation when $\sigma = 1.5$ 51
S78 Boxplots of the ARI resulting from CC and PAM on simulated data. . . 52
S79 Boxplots of the sensitivity with CC and PAM when $\sigma = 0.2$ 52
S80 Boxplots of the sensitivity with CC and PAM when $\sigma = 0.5$ 53
S81 Boxplots of the sensitivity with CC and PAM when $\sigma = 1$ 54
S82 Boxplots of the sensitivity with CC and PAM when $\sigma = 1.5$ 54
S83 Boxplots of the PPV with CC and PAM when $\sigma = 0.2$ 55
S84 Boxplots of the PPV with CC and PAM when $\sigma = 0.5$ 55
S85 Boxplots of the PPV with CC and PAM when $\sigma = 1$ 56
S86 Boxplots of the PPV with CC and PAM when $\sigma = 1.5$ 56
S87 Boxplots of the geometric accuracy with CC and PAM when $\sigma = 0.2$ . . 57
S88 Boxplots of the geometric accuracy with CC and PAM when $\sigma = 0.5$ . . 57
S89 Boxplots of the geometric accuracy with CC and PAM when $\sigma = 1$ . . . 58
S90 Boxplots of the geometric accuracy (Acc_g) with CC and PAM when $\sigma = 1.5$ 58
S91 Boxplots of the ARI resulting from CC and Spectral clustering on simulated data. 59
S92 Boxplots of the sensitivity with CC and Spectral clustering when $\sigma = 0.2$ 59
S93 Boxplots of the sensitivity with CC and Spectral clustering when $\sigma = 0.5$ 60
S94 Boxplots of the sensitivity with CC and Spectral clustering when $\sigma = 1$ 60
S95 Boxplots of the sensitivity with CC and Spectral clustering when $\sigma = 1.5$ 61
S96 Boxplots of the PPV with CC and Spectral clustering when $\sigma = 0.2$ . . 62
S97 Boxplots of the PPV with CC and Spectral clustering when $\sigma = 0.5$ . . 62
S98 Boxplots of the PPV with CC and Spectral clustering when $\sigma = 1$ . . . 63
S99 Boxplots of the PPV with CC and Spectral clustering when $\sigma = 1.5$ . . 63
S100 Boxplots of the geometric accuracy with CC and Spectral clustering when $\sigma = 0.2$ 64
S101 Boxplots of the geometric accuracy with CC and Spectral clustering when $\sigma = 0.5$ 64
S102 Boxplots of the geometric accuracy with CC and Spectral clustering when $\sigma = 1$ 65
S103 Boxplots of the geometric accuracy with CC and Spectral clustering when $\sigma = 1.5$ 65
S104 Boxplots reporting the number of clusters detected on simulated data with $\sigma = 0.2$ by CC and CL method using different methods. 66
S105 Boxplots reporting the number of clusters detected on simulated data with $\sigma = 0.5$ by CC and CL method using different methods. 67
S106 Boxplots reporting the number of clusters detected on simulated data with $\sigma = 1$ by CC and CL method using different methods. 68

S107 Boxplots reporting the number of clusters detected on simulated data with $\sigma = 1.5$ by CC and CL method using different methods. 69
S108 Boxplots reporting the number of clusters detected on simulated data with $\sigma = 0.2$ by CC and Ward using different methods. 70
S109 Boxplots reporting the number of clusters detected on simulated data with $\sigma = 0.5$ by CC and Ward using different methods. 71
S110 Boxplots reporting the number of clusters detected on simulated data with $\sigma = 1$ by CC and Ward using different methods. 72
S111 Boxplots reporting the number of clusters detected on simulated data with $\sigma = 1.5$ by CC and Ward using different methods. 73
S112 Boxplots reporting the number of clusters detected on simulated data with $\sigma = 0.2$ by CC and K-means using different methods. 74
S113 Boxplots reporting the number of clusters detected on simulated data with $\sigma = 0.5$ by CC and K-means using different methods. 75
S114 Boxplots reporting the number of clusters detected on simulated data with $\sigma = 1$ by CC and K-means using different methods. 76
S115 Boxplots reporting the number of clusters detected on simulated data with $\sigma = 1.5$ by CC and K-means using different methods. 77
S116 Representation of the boundaries of the ten different intervals used for choosing the number of clusters in the CC algorithm. 78
S117 Boxplots of the number of clusters found by CC with ten different intervals and $\sigma = 0.2$. 79
S118 Boxplots of the number of clusters found by CC with ten different intervals and $\sigma = 0.5$. 80
S119 Boxplots of the number of clusters found by CC with ten different intervals and $\sigma = 1$. 81
S120 Boxplots of the number of clusters found by CC with ten different intervals and $\sigma = 1.5$. 82
S121 Dendrogram resulting from clustering 42 brain tumors samples with Ward. 84
S122 Dendrogram resulting from clustering 42 brain tumors samples with CL. 85
S123 Dendrogram resulting from clustering 30 breast cancer tumor samples with Ward on Euclidean distance. 90
S124 Dendrogram resulting from clustering 30 breast cancer tumor samples with CL on Euclidean distance. 91
S125 Graphical representation of the true membership (first row) of the 572 specimens of oliveoil in olive oil data, compared with the memberships resulting from CC, DBSCAN, SOM, K-means, CL, and Ward. 93
S126 Kaplan-Meier survival curves of the 2 clusters obtained with CC on the HM subgroup of the Jacob dataset. 95
S127 Kaplan-Meier survival curves of the 2 clusters obtained with CC on the TM subgroup of the Jacob dataset. The log-rank test p-value for the difference among the survival curves resulted equal 0.0426. 95
S128 Boxplots of the information gain computed over Ward's results on 100 simulated datasets with $\sigma = 0.5$. 97
S129 Boxplots of the information gain computed over CL results on 100 simulated datasets with $\sigma = 0.5$. 98
S130 Boxplots of the information gain computed over CC results on 100 simulated datasets with $\sigma = 0.5$. 99

List of Tables

S1 Example of a contingency matrix that will end up with an empty cluster. 6

S2 Results in the CC example from the simulated data at step 5 of the algorithm. 7
S3 Example of the contingency matrix used in the CC algorithm. 7
S4 The average information gained and its standard deviation, computed for Ward, CL, CC on simulated data. 18
S5 Percentages of success with K-means, Ward, CL and CC for $\sigma = 0.2, 0.5, 1, 1.5$ in correspondence with different indexes. 37
S6 Combinations of x and y dimensions for the SOM's grid. 83
S7 Contingency table reporting both the real classification in subtypes and the clusters identified by Ward on the brain tumors dataset. 86
S8 Contingency table reporting both the real classification in subtypes and the clusters identified by the CL method on the brain tumors dataset. . 86
S9 Contingency table reporting both the real classification in subtypes and the clusters identified by the K-means method on the brain tumors dataset. 86
S10 Contingency table reporting both the real classification in subtypes and the clusters identified by the SOM method on the brain tumors dataset. 86
S11 Contingency table reporting both the real classification in subtypes and the clusters identified by CC on the brain tumors dataset. 87
S12 Summarization of results on brain tumors dataset with Euclidean distance. 87
S13 Summarization of results on breast cancer dataset with Euclidean distance. 88
S14 Contingency table reporting both the real classification in subtypes and the clusters identified by Ward on the breast cancer dataset. 88
S15 Contingency table reporting both the real classification in subtypes and the clusters identified by CL on the breast cancer dataset. 88
S16 Contingency table reporting both the real classification in subtypes and the clusters identified by SOM on the breast cancer dataset. 89
S17 Contingency table reporting both the real classification in subtypes and the clusters identified by K-means on the breast cancer dataset. 89
S18 Contingency table reporting both the real classification in subtypes and the clusters identified by CC on the breast cancer dataset. 89
S19 Summarization of results on the olive oil dataset with Euclidean distance. 92
S20 Contingency table reporting both the real classification in subtypes and the clusters identified by Ward method on the olive oil dataset. 92
S21 Contingency table reporting both the real classification in subtypes and the clusters identified by CL method on the olive oil dataset. 92
S22 Contingency table reporting both the real classification in subtypes and the clusters identified by K-means method on the olive oil dataset. . . 94
S23 Contingency table reporting both the real classification in subtypes and the clusters identified by SOM method on the olive oil dataset. 94
S24 Contingency table reporting both the real classification in subtypes and the clusters identified by CC method on the olive oil dataset. 94
S25 Running times of different methods in clustering the same 100 simulated data sets, sorted by increasing average time. 96

Average Silhouette Width

The Silhouette Width [1] is defined as the average of the degree of confidence of an element to be in a cluster. For an element i:

$$S(i) = \frac{b_i - a_i}{max(b_i, a_i)},$$

where a_i is average distance between i and elements in the same cluster, and b_i is the average distance between i and elements in the nearest cluster. $S(i)$ lies in $[-1, 1]$ and should be maximized. Taking the average of the $S(i)$ for all objects i belonging to that cluster we obtain the Average Silhouette Width (ASW), which allows us to identify weak clusters.

Special cases for Cross-clustering

Multiple optimal contingency matrices

There could be cases in which more than one A is associated with the same maximum number of clustered elements. In order to choose a unique solution, the largest ASW is used to select the best combination of $\mathbf{k}_j^C$ and $\mathbf{k}_i^W$. In the case of more than one partition obtaining the same maximum ASW, the algorithm will choose the one with the smaller number of clusters in the Ward algorithm.

Empty clusters

		**CL**			
		Cluster 1	Cluster 2	Cluster 3	Cluster 4
**Ward**	Cluster 1	5	0	0	0
	Cluster 2	0	2	0	4
	Cluster 3	6	0	3	0

Table S1. Example of a contingency matrix that will end up with an empty cluster.

The algorithm may end in empty clusters, an example is the case of the contingency table in Table S1. Here A^* starts as an empty matrix of size 3×3. The algorithm choses $\max(\mathrm{A}) = 6$, it puts it in $A^*[1, 1]$ and removes the row and column where max(A) appears, obtaining the sub-matrix A^-. Then, the algorithm sets $A^*[2, 2] = \max(\mathrm{A}^-) = 4$, removes the row and column where $\max(\mathrm{A}^-)$ appears, obtaining a sub-matrix A^{2-} which is of dimensions 1×2 and contains only zero values. In the last available cell on the diagonal of A^* we will put $\max(\mathrm{A}^{2-}) = 0$. In this case, such clusters are simply eliminated and the number of clusters will be decreased, also in the case of just one left cluster.

Example of Cross-clustering

Data in a 10×7 matrix have been simulated with the rows representing genes and columns identifying samples. The random values of the first two samples are drawn from $\mathcal{N}(\mu = 10, \sigma = 0.1)$, the values of samples 3 and 4 are drawn from $\mathcal{N}(\mu = 20, \sigma = 0.1)$, the values of samples 5 and 6 are drawn from $\mathcal{N}(\mu = 5, \sigma = 0.1)$, while the last sample is designed to be the outlier, with values drawn from $\mathcal{U}(0, 1)$. We used RStudio (version 0.98.507) [2] setting a seed of 123.

Ward	CL	Elements classified	ASW
2	3	5	-
2	4	4	-
2	5	3	-
2	6	3	-
3	4	**6**	0.84
3	5	5	-
3	6	4	-
4	5	**6**	0.56
4	6	5	-
5	6	**6**	0.28

Table S2. Results from the simulated data at step 5 of the algorithm. The first two columns represent the number of clusters in Ward's minimum variance and CL methods, while the third one shows how many samples have been classified together simultaneously by both algorithms. The last column reports the average Silhouette Width (ASW) computed in cases of multiple maximum values of elements classified (6, in this case) in order to choose the optimal combination.

		**CL**			
		Cluster 1	Cluster 2	Cluster 3	Cluster 4
**Ward**	Cluster 1	0	2	0	0
	Cluster 2	0	0	2	1
	Cluster 3	2	0	0	0

Table S3. Contingency matrix A obtained after step 7 of CC algorithm on simulated data when the number of clusters for Ward algorithm is set to 3 and for CL is set to 4.

The distance chosen for the algorithm is Euclidean, as it is the most commonly used metric [3]. In this example the interval of plausible number of clusters is easy to set and will be between $n_{W_{min}} = 2$ and $n_{W_{max}} = 5$ for the Ward algorithm, to let the Complete-linkage (CL) have always a higher number of clusters ($n_{C_{min}} = 3$ and $n_{C_{max}} = 6$) and be able to isolate the outlier. This results in 10 contingency tables A with a number of rows varying between 2 and 5, and a number of columns varying between 3 and 6, representing all the possible combinations of Ward and CL partitions. The list of possible combinations is shown in Table S2. An example of a combination is shown in Table S3, where the number of clusters for the Ward algorithm is set equal to 3 and with the CL is set equal to 4. A^* starts as an empty matrix of size $n^*_{W_i} \times n^*_{C_j}$. The algorithm chooses max(A), it puts it in $A^*[1,1]$ and removes the row and column where max(A) appears, obtaining a sub-matrix A^-. Then, the algorithm sets $A^*[2,2]$ as max(A^-), removes the row and column where max(A^-) appears, obtaining a sub matrix of A^-, defined as A^{2-}. Lastly, it sets $A^*[3,3]$ as max(A^{2-}), removes the rows and columns where max(A^{2-}) appears, and stops, as an empty matrix is obtained. In this case A^* is a 3×3 matrix with all 2 on the diagonal. Cross-clustering (CC) repeats this operation for every combination of number of clusters. In this example we obtain 10 different A^* matrices. The pairs that maximize the sum of the diagonal (representing the maximum number of elements clustered together by both Ward and CL) is obtained for combinations [(3,4), (4,5), (5,6)]. The combination (3,4) is chosen as it yields the largest ASW of 0.84. CC here suggests 3 clusters of the first three pairs of samples, excluding the last sample, which is correctly identified as an outlier.

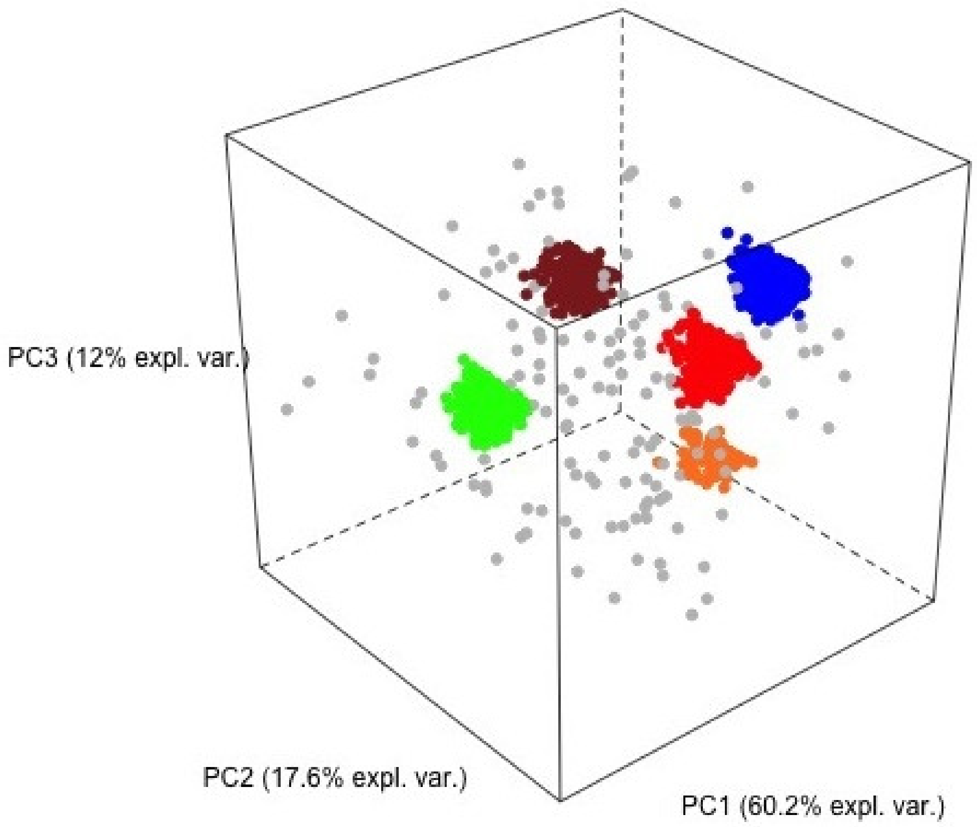

Figure S1. Principal Component Analysis plot of one simulation of the data with the highest variance ($\sigma = 1.5$) on the first three principal components. Each group of points corresponds to one of the five prototypes used to define the general behavior of the genes. The gray points represent the 150 completely random genes added to the set. The cumulative percentage of variance explained is 89.8%. Categories are well defined even when the variability added to the data is high, while the outlier genes are distributed uniformly around the center.

Simulation study

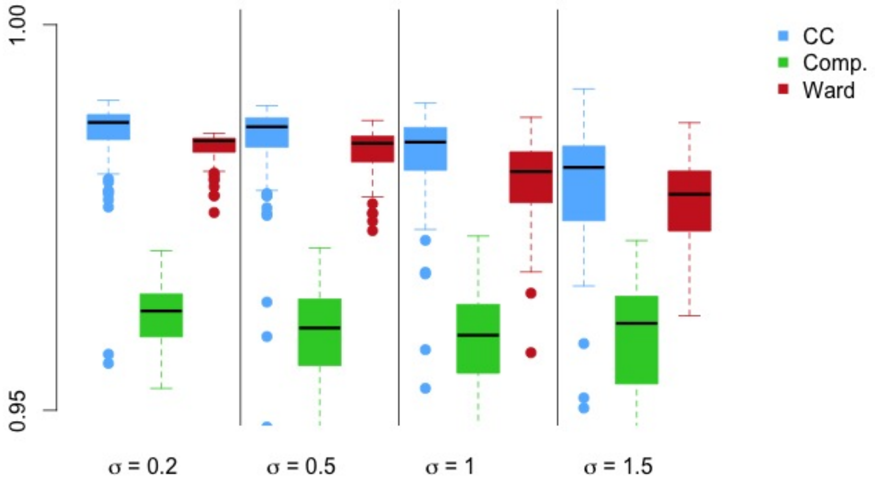

Figure S2. Boxplots of the Adjusted Rand Index (ARI) resulting from applying CC, CL, and Ward's algorithm (all with Euclidean distance) with $\sigma = 0.2, 0.5, 1, 1.5$ on simulated data. The ARI here is used to measure the agreement between the obtained partition with each method and the real partition (the higher, the better), proving that CC performs at least as well as the two methods that constitute it. The y-axis has been cut in order to focus on the central part of the distribution and not on outlier values.

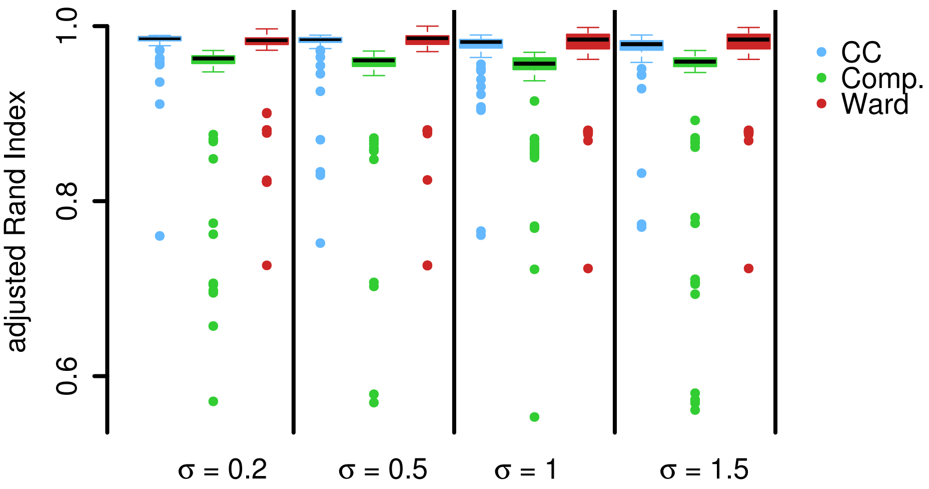

Figure S3. Boxplots of the Adjusted Rand Index (ARI) resulting from CC, CL, and Ward's algorithm (all with Chebychev distance in order to check if the choice of the distance undermines the quality of results) with $\sigma = 0.2, 0.5, 1, 1.5$ on simulated data. The ARI here is used to measure the agreement between the obtained partition with each method and the real partition, proving that CC performs at least as well as the two methods that constitute it.

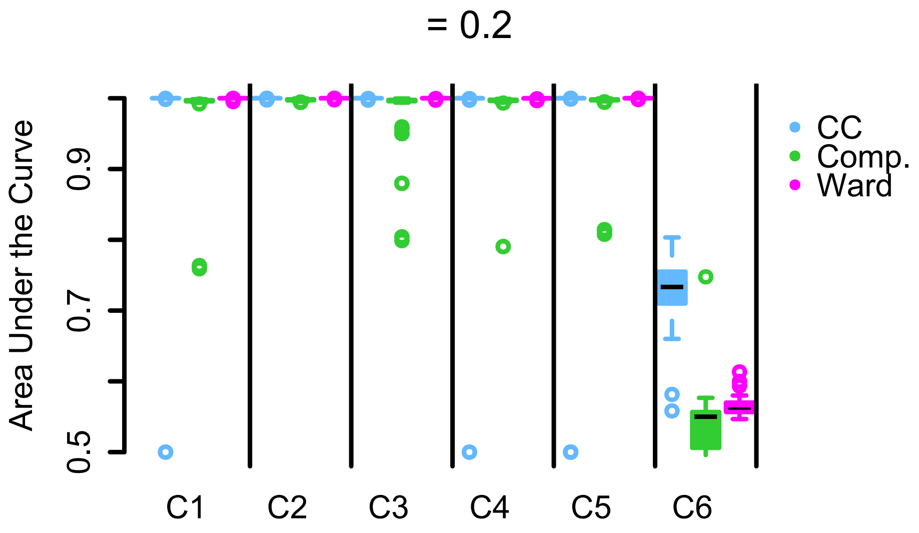

Figure S4. Boxplots of the Area Under the Curve (AUC) in each of the $K = 6$ simulated clusters for $S = 100$ simulations with CC, CL, and Ward algorithm when $\sigma = 0.2$. High AUC values represent high true positive rates and low false positive rates, thus, the higher the better. In the first clusters the performance is approximately the same, while in the last group, which is the outliers one, CC performs better.

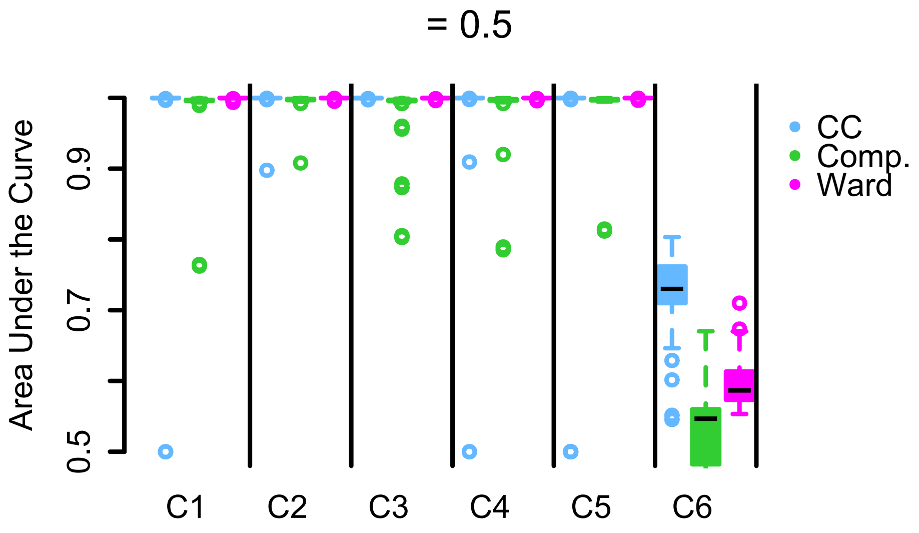

Figure S5. Boxplots of the Area Under the Curve (AUC) in each of the $K = 6$ simulated clusters for $S = 100$ simulations with CC, CL, and Ward algorithm when $\sigma = 0.5$. High AUC values represent high true positive rates and low false positive rates, thus, the higher the better. In the first clusters the performance is approximately the same, while in the last group, which is the outliers one, CC performs better.

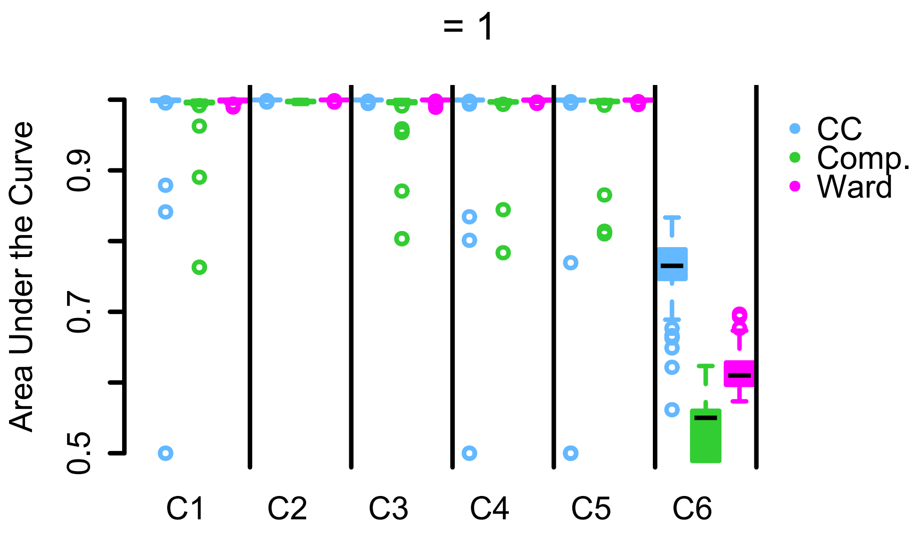

Figure S6. Boxplots of the Area Under the Curve (AUC) in each of the $K = 6$ simulated clusters for $S = 100$ simulations with CC, CL, and Ward algorithm when $\sigma = 1$. High AUC values represent high true positive rates and low false positive rates, thus, the higher the better. In the first clusters the performance is approximately the same, while in the last group, which is the outliers one, CC performs better.

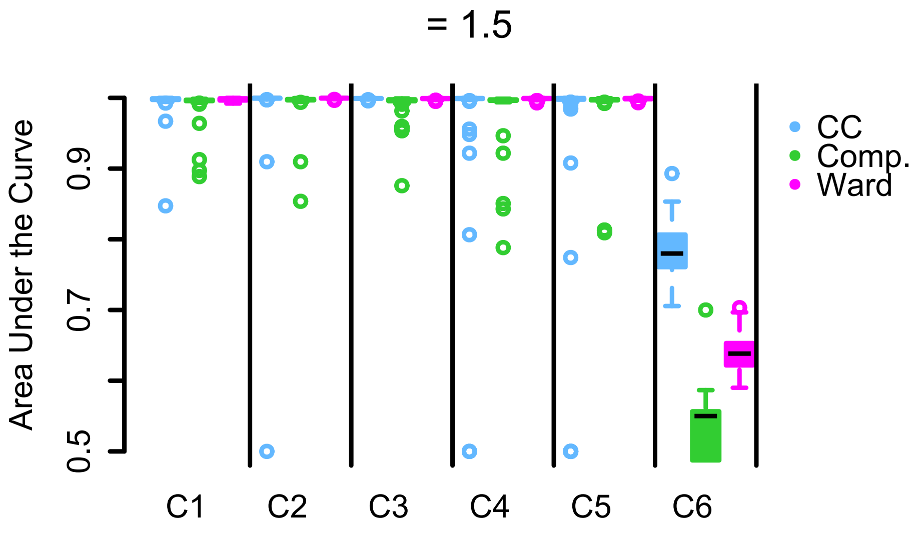

Figure S7. Boxplots of the Area Under the Curve (AUC) in each of the $K = 6$ clusters for $S = 100$ simulations with CC, CL, and Ward algorithm in the case of highest variability ($\sigma = 1.5$). High AUC values represent high true positive rates and low false positive rates, thus, the higher the better. In the first clusters the performance is approximately the same, while in the last group, which is the outliers one, CC performs better.

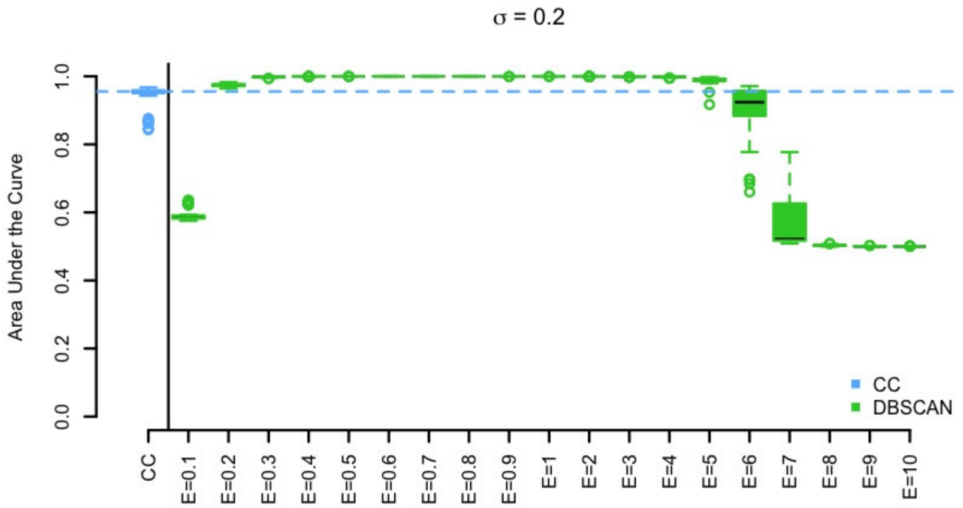

Figure S8. Boxplots of the average Area Under the Curve (AUC) over the $K = 6$ simulated clusters coming from CC and DBSCAN where ϵ is changing ($\epsilon \in [0.1, 10]$) and the minimum number of points within ϵ is set equal 5 (as by default in the R package used), when $\sigma = 0.2$. High values represent high true positive rates and low false positive rates, thus, the higher the better. The horizontal line represents CC's median value, which is higher than most of the values resulting from DBSCAN. This figure shows how sensitive DBSCAN is to the choice of parameters.

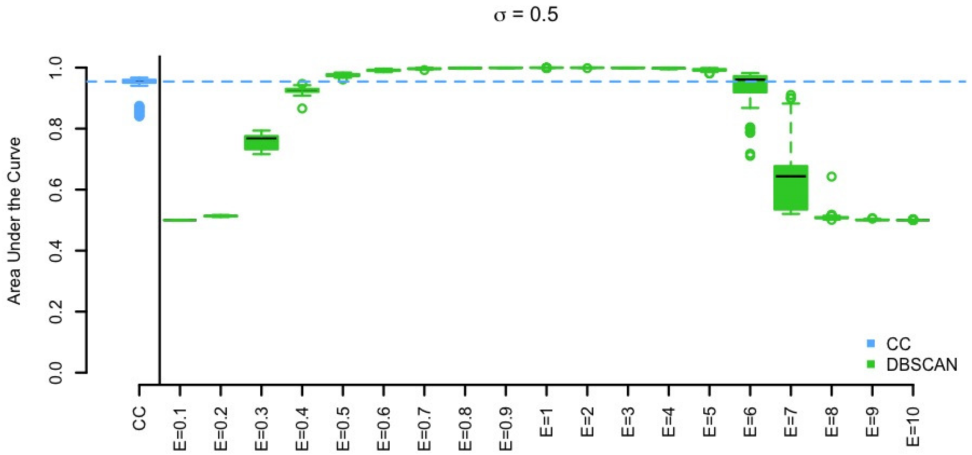

Figure S9. Boxplots of the average Area Under the Curve (AUC) over the $K = 6$ simulated groups coming from CC and DBSCAN where ϵ is changing ($\epsilon \in [0.1, 10]$) and the minimum number of points within ϵ is set equal 5 (as by default), when $\sigma = 0.5$. High values represent high true positive rates and low false positive rates, thus, the higher the better. The horizontal line represents CC's median value, which is higher than most of the values resulting from DBSCAN. This figure shows how sensitive DBSCAN is to the choice of parameters.

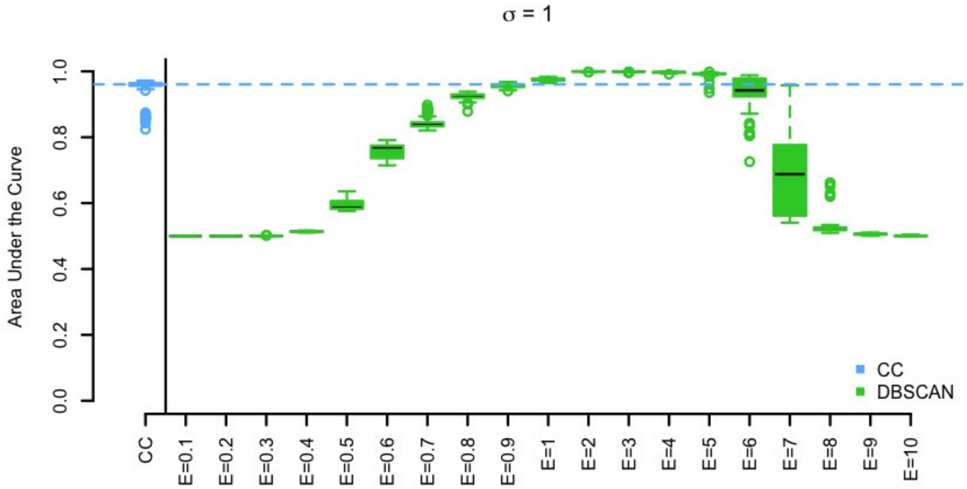

Figure S10. Boxplots of the average Area Under the Curve (AUC) over the $K = 6$ simulated groups coming from CC and DBSCAN where ϵ is changing ($\epsilon \in [0.1, 10]$) and the minimum number of points within ϵ is set equal 5 (as by default), when $\sigma = 1$. High values represent high true positive rates and low false positive rates, thus, the higher the better. The horizontal line represents CC's median value, which is higher than most of the values resulting from DBSCAN. This figure shows how sensitive DBSCAN is to the choice of parameters.

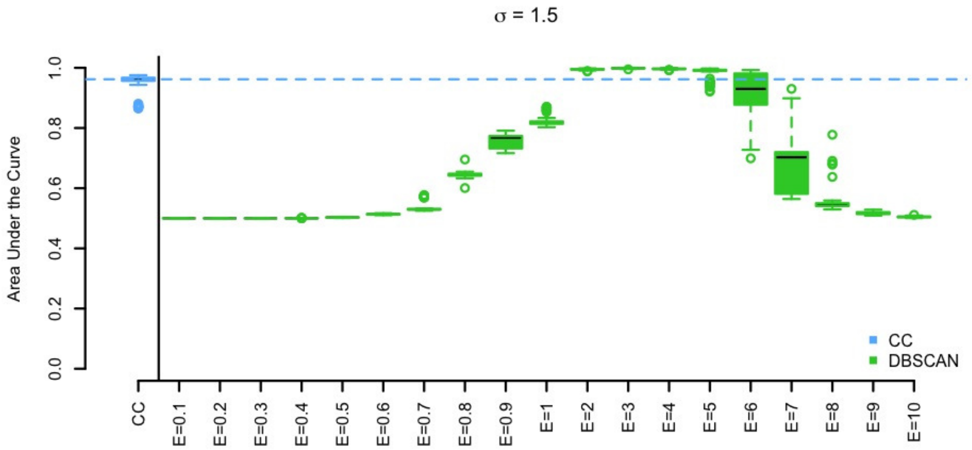

Figure S11. Boxplots of average Area Under the Curve over the $K = 6$ simulated clusters coming from CC and DBSCAN where ϵ is changing ($\epsilon \in [0.1, 10]$) and the minimum number of points within ϵ is set equal 5 (as by default), in the case of highest variability in data ($\sigma = 1.5$). High values represent high true positive rates and low false positive rates, thus, the higher the better. The horizontal line represents CC's median value, which is higher than most of the values resulting from DBSCAN. This figure shows how sensitive DBSCAN is to the choice of parameters.

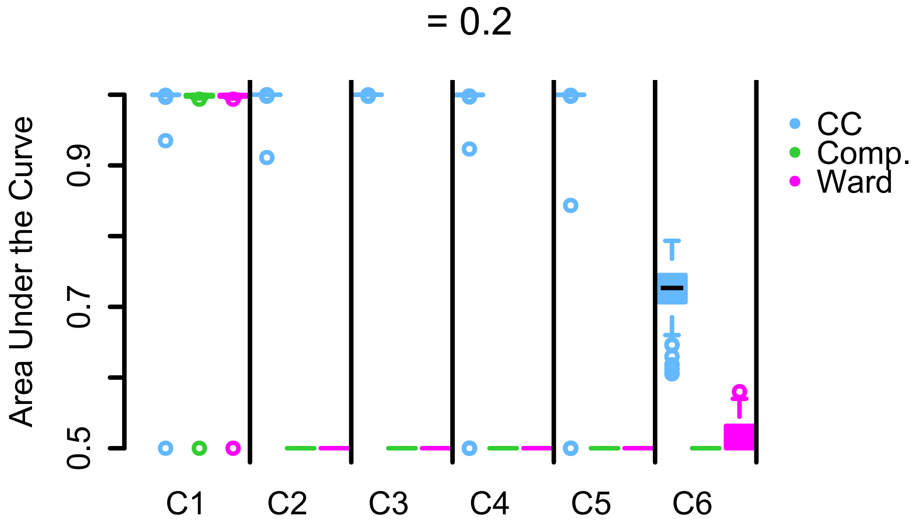

Figure S12. Boxplots of the Area Under the Curve (AUC) in each of the $K = 6$ simulated clusters for $S = 100$ simulations with CC, CL, and Ward algorithm when $\sigma = 0.2$ using the Chebychev distance in order to check if the choice of the distance undermines the quality of results. High AUC values represent high true positive rates and low false positive rates, thus, the higher the better. In the first clusters the performance is approximately the same, while in the last group, which is the outliers one, CC performs better.

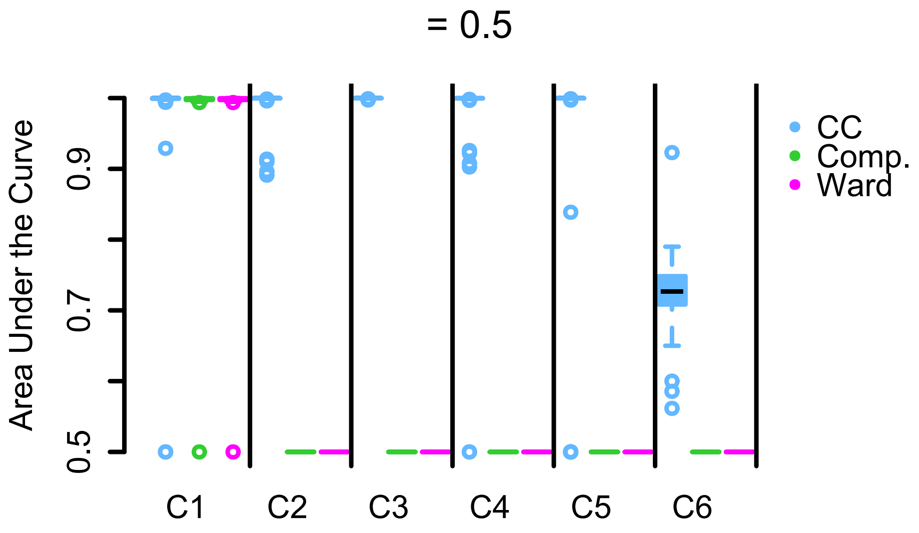

Figure S13. Boxplots of the Area Under the Curve in each of the $K = 6$ simulated clusters for $S = 100$ simulations with CC, CL, and Ward algorithm when $\sigma = 0.5$ using the Chebychev distance. The last group is the outliers one. As the CC's AUC is always at least as well as the competitors', we can conclude that it performs better than them in terms of sensitivity and specificity.

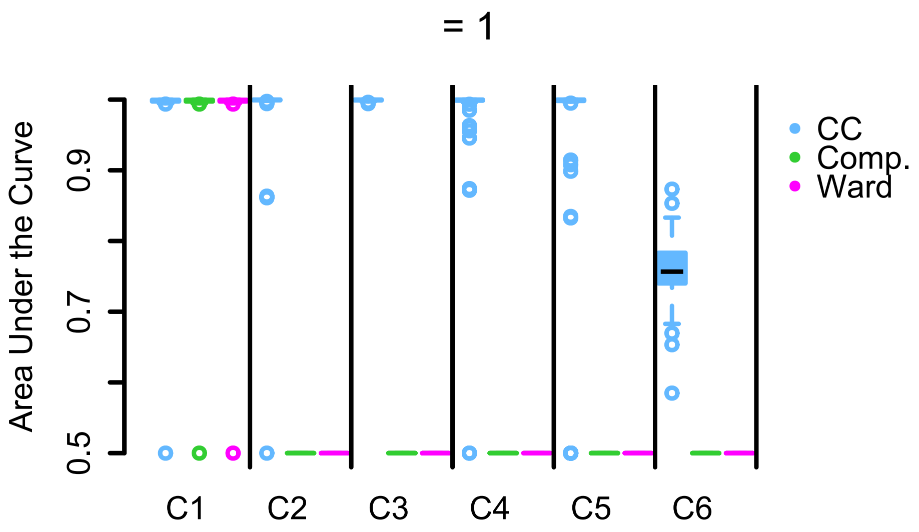

Figure S14. Boxplots of the Area Under the Curve in each of the $K = 6$ simulated clusters for $S = 100$ simulations with CC, CL, and Ward algorithm when $\sigma = 1$ using the Chebychev distance in order to check if the choice of the distance undermines the quality of results. High AUC values represent high true positive rates and low false positive rates, thus, the higher the better. In the first clusters the performance is approximately the same, while in the last group, which is the outliers one, CC performs better.

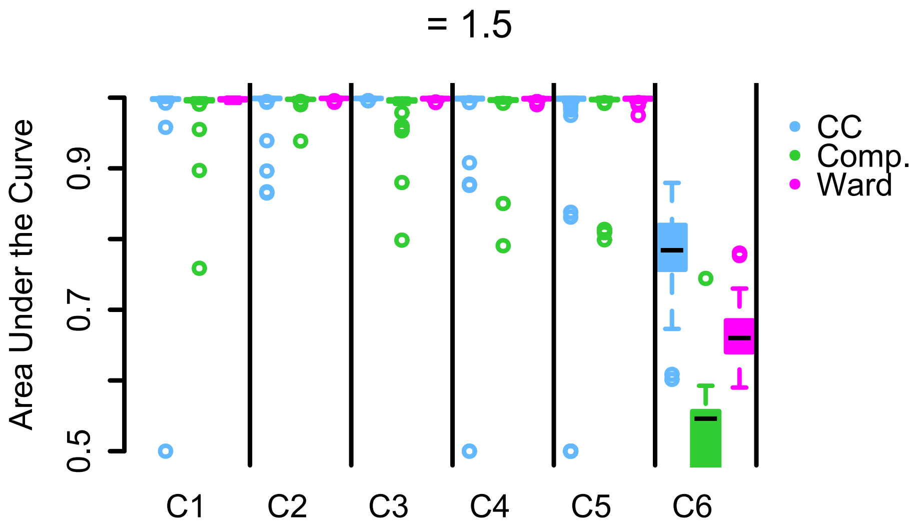

Figure S15. Boxplots of the Area Under the Curve in each of the $K = 6$ simulated clusters for $S = 100$ simulations with CC, CL, and Ward algorithm when $\sigma = 1.5$ using the Chebychev distance in order to check if the choice of the distance undermines the quality of results. High AUC values represent high true positive rates and low false positive rates, thus, the higher the better. In the first clusters the performance is approximately the same, while in the last group, which is the outliers one, CC performs better.

**Methods:**	$\sigma = 0.2$	$\sigma = 0.5$	$\sigma = 1$	$\sigma = 1.5$
**Ward**	1.0055 ± 0.1655	1.0066 ± 0.1623	1.0009 ± 0.1565	0.9909 ± 0.1523
**CL**	0.8136 ± 0.2307	0.8107 ± 0.2305	0.8126 ± 0.2272	0.7946 ± 0.2350
**CC**	1.0681 ± 0.0146	1.0627 ± 0.0276	1.0480 ± 0.0525	1.0382 ± 0.0432

Table S4. The average information gained along with its standard deviation, computed for each of the considered methods (Ward, CL, CC) over all the number of clusters (in Ward and CL the number was ranging between 2 and 20, in CC the algorithm automatically determined it) in each of the 100 simulations for every σ added to the data (0.2, 0.5, 1.0, 1.5), is reported in the above table. Here you can see that CC led always to the highest information gain and to the lower standard deviation, meaning that the quality of the clusters in terms of purity is always higher when using CC.

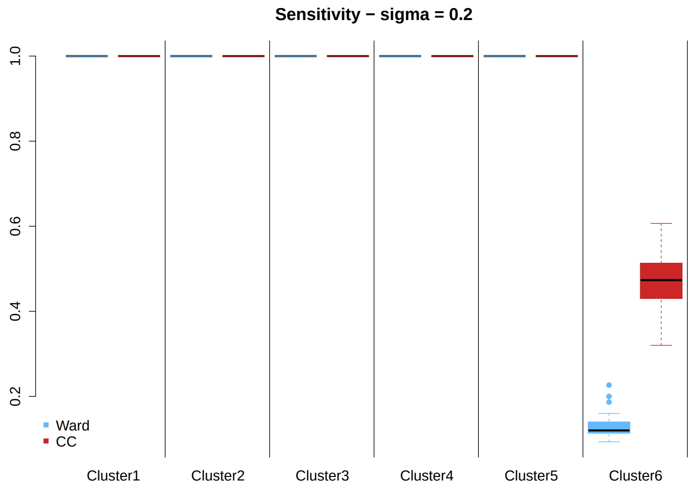

Figure S16. Boxplots of the Sensitivity in each of the $K = 6$ simulated clusters for $S = 100$ simulations with CC and Ward when $\sigma = 0.2$. The sensitivity is the rate between the number of true positives and the sum of true positives and false negatives. In the first clusters the performance is approximately the same, while in the last cluster CC performs better.

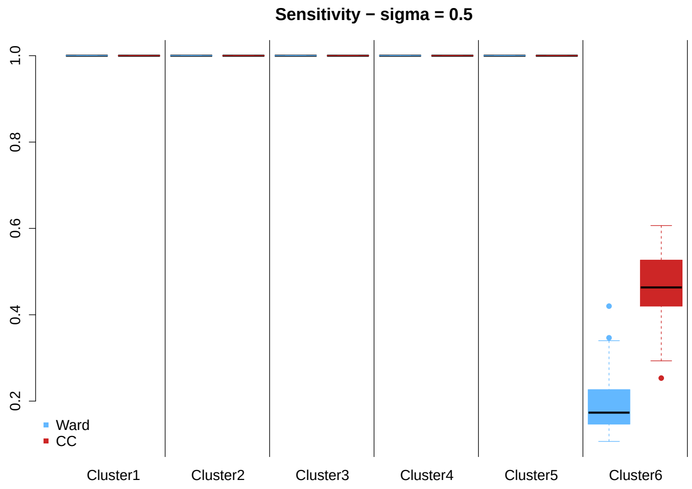

Figure S17. Boxplots of the Sensitivity in each of the $K = 6$ simulated clusters for $S = 100$ simulations with CC and Ward when $\sigma = 0.5$. The sensitivity is the rate between the number of true positives and the sum of true positives and false negatives. In the first clusters the performance is approximately the same, while in the last cluster CC performs better.

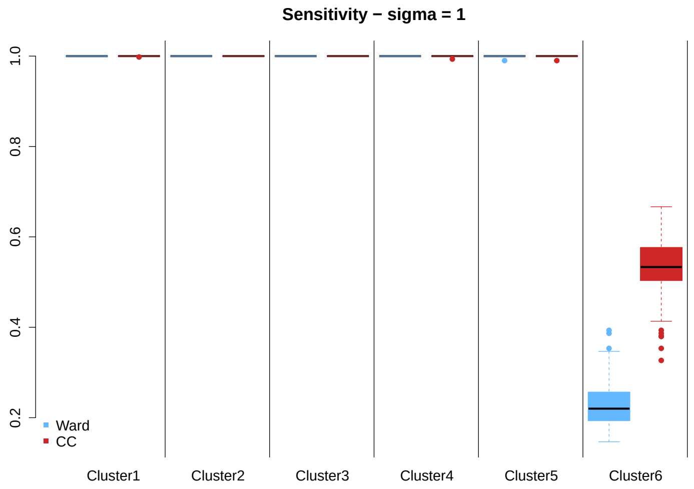

Figure S18. Boxplots of the Sensitivity in each of the $K = 6$ simulated clusters for $S = 100$ simulations with CC and Ward when $\sigma = 1$. The sensitivity is the rate between the number of true positives and the sum of true positives and false negatives. In the first clusters the performance is approximately the same, while in the last cluster CC performs better.

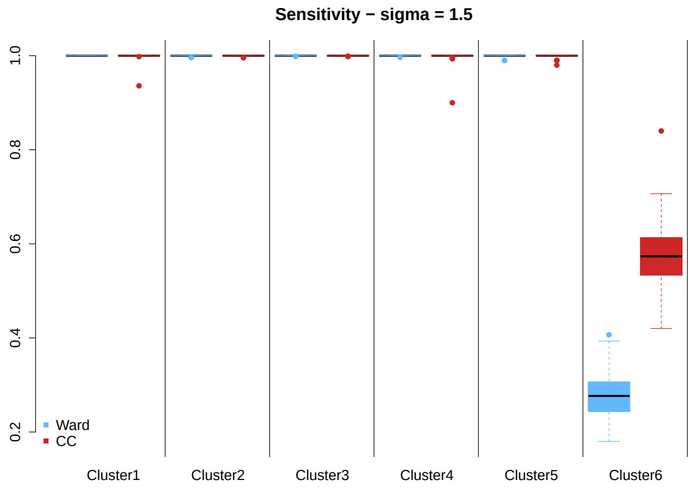

Figure S19. Boxplots of the sensitivity in each of the $K = 6$ simulated clusters for $S = 100$ simulations with CC and Ward when $\sigma = 1.5$. The sensitivity is the rate between the number of true positives and the sum of true positives and false negatives. In the first clusters the performance is approximately the same, while in the last cluster CC performs better.

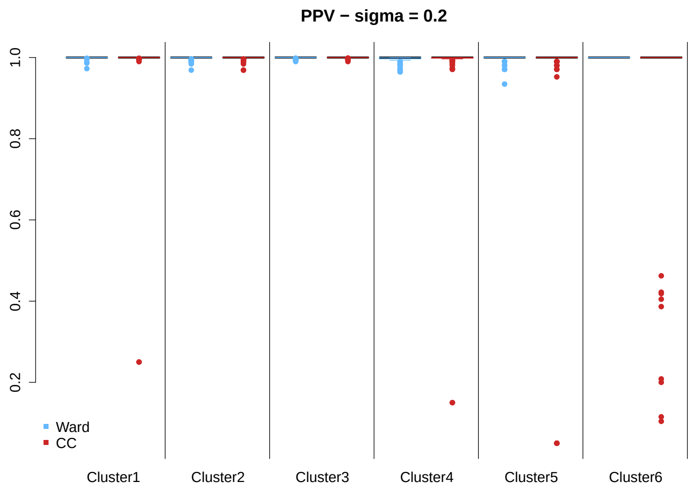

Figure S20. Boxplots of the PPV in each of the $K = 6$ simulated clusters for $S = 100$ simulations with CC and Ward when $\sigma = 0.2$. The PPV is the rate between the number of true positives and the sum of true positives and false positives. They perform approximately in the same way.

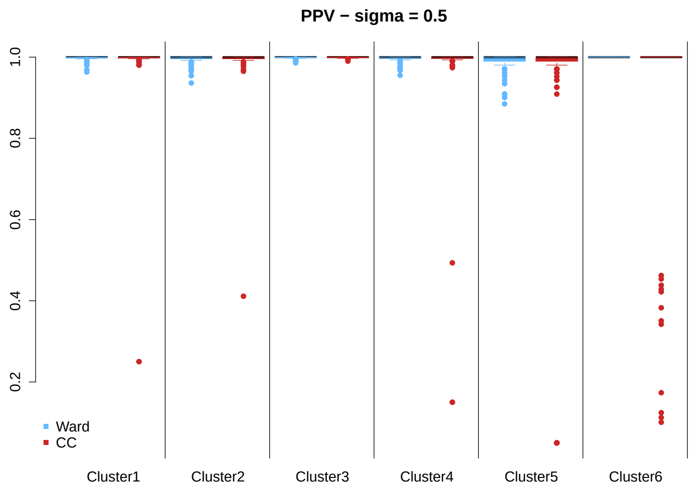

Figure S21. Boxplots of the PPV in each of the $K = 6$ simulated clusters for $S = 100$ simulations with CC and Ward when $\sigma = 0.5$. The PPV is the rate between the number of true positives and the sum of true positives and false positives. They perform approximately in the same way.

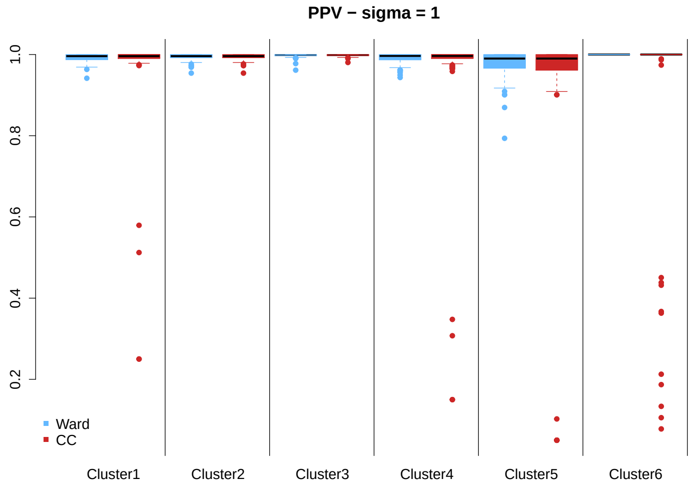

Figure S22. Boxplots of the PPV in each of the $K = 6$ simulated clusters for $S = 100$ simulations with CC and Ward when $\sigma = 1$. The PPV is the rate between the number of true positives and the sum of true positives and false positives. They perform approximately in the same way.

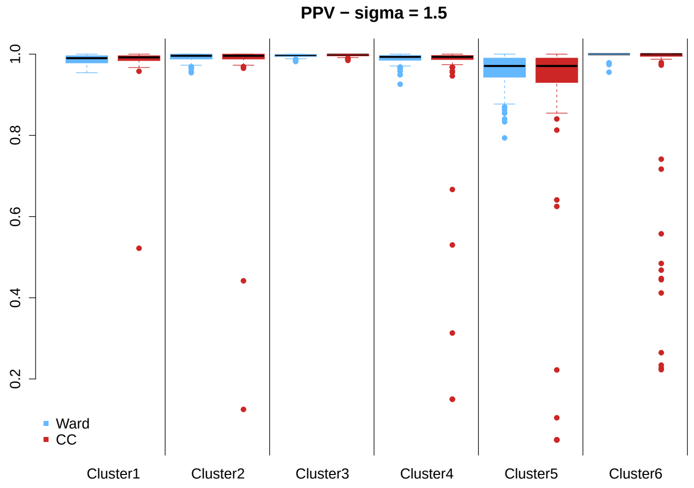

Figure S23. Boxplots of the PPV in each of the $K = 6$ simulated clusters for $S = 100$ simulations with CC and Ward when $\sigma = 1.5$. The PPV is the rate between the number of true positives and the sum of true positives and false positives. They perform approximately in the same way.

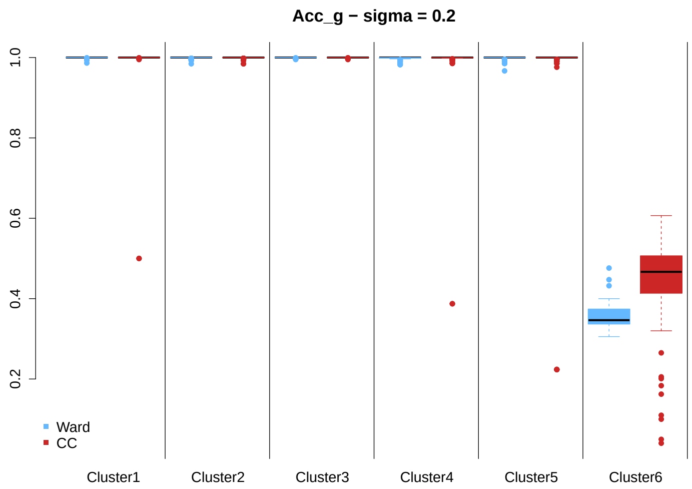

Figure S24. Boxplots of the Geometric Accuracy (Acc_g) in each of the $K = 6$ simulated clusters for $S = 100$ simulations with CC andWard when $\sigma = 0.2$. The Geometric Accuracy is the geometric mean of sensitivity and PPV. CC performed always better.

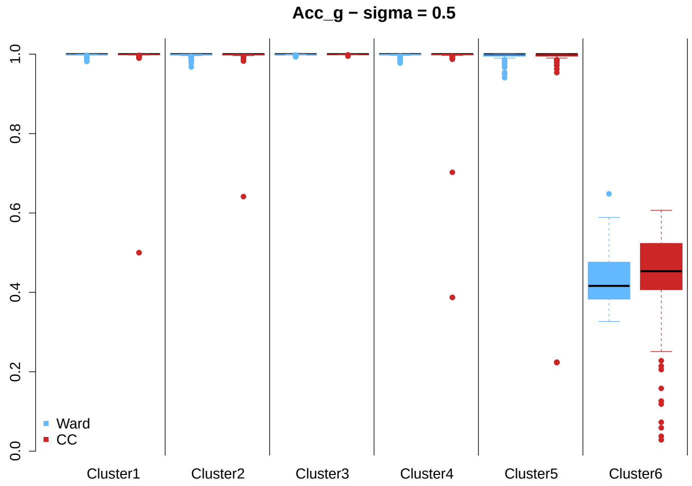

Figure S25. Boxplots of the Geometric Accuracy (Acc_g) in each of the $K = 6$ simulated clusters for $S = 100$ simulations with CC and Ward when $\sigma = 0.5$.The Geometric Accuracy is the geometric mean of sensitivity and PPV. CC performed always better.

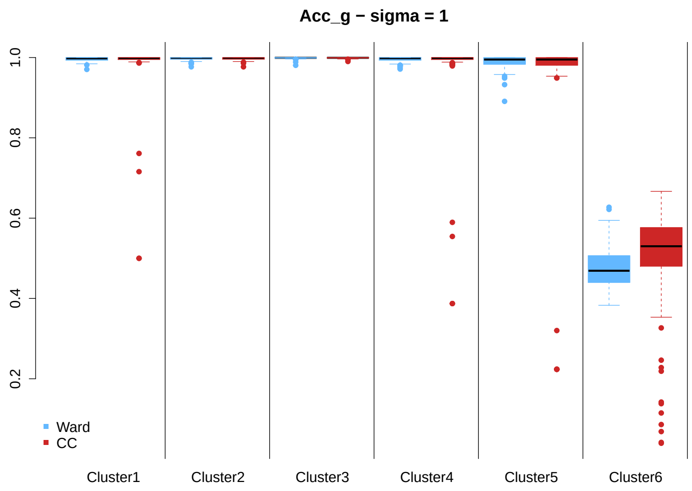

Figure S26. Boxplots of the Geometric Accuracy (Acc_g) in each of the $K = 6$ simulated clusters for $S = 100$ simulations with CC and Ward when $\sigma = 1$. The Geometric Accuracy is the geometric mean of sensitivity and PPV. CC performed always better.

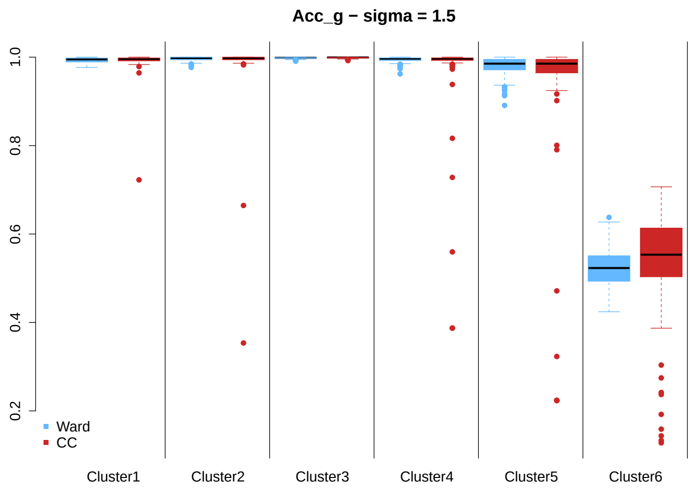

Figure S27. Boxplots of the Geometric Accuracy (Acc_g) in each of the $K = 6$ simulated clusters for $S = 100$ simulations with CC and Ward when $\sigma = 1.5$. The Geometric Accuracy is the geometric mean of sensitivity and PPV. CC performed always better.

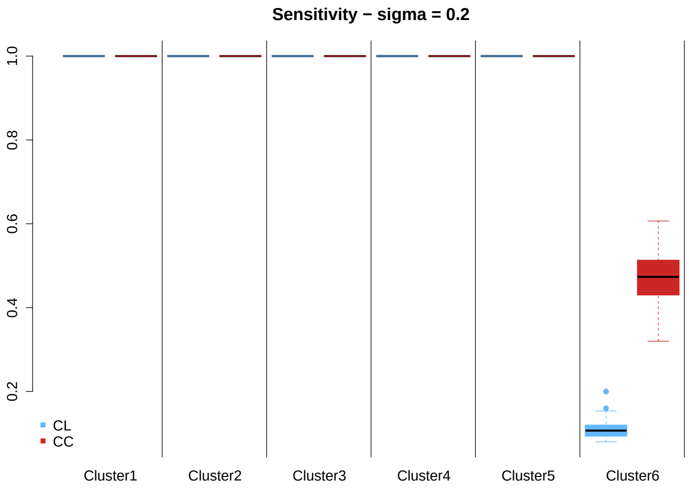

Figure S28. Boxplots of the sensitivity in each of the $K = 6$ simulated clusters for $S = 100$ simulations with CC and Complete-linkage when $\sigma = 0.2$. The sensitivity is the rate between the number of true positives and the sum of true positives and false negatives. In the first clusters the performance is approximately the same, while in the last cluster CC performs better.

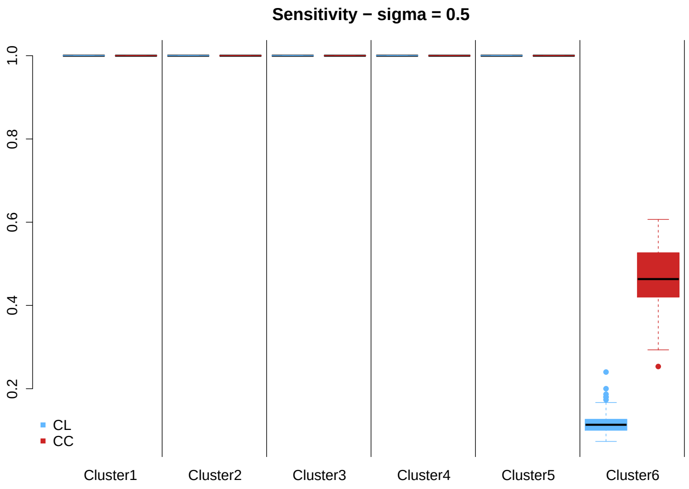

Figure S29. Boxplots of the sensitivity in each of the $K = 6$ simulated clusters for $S = 100$ simulations with CC and Complete-linkage when $\sigma = 0.5$. The sensitivity is the rate between the number of true positives and the sum of true positives and false negatives. In the first clusters the performance is approximately the same, while in the last cluster CC performs better.

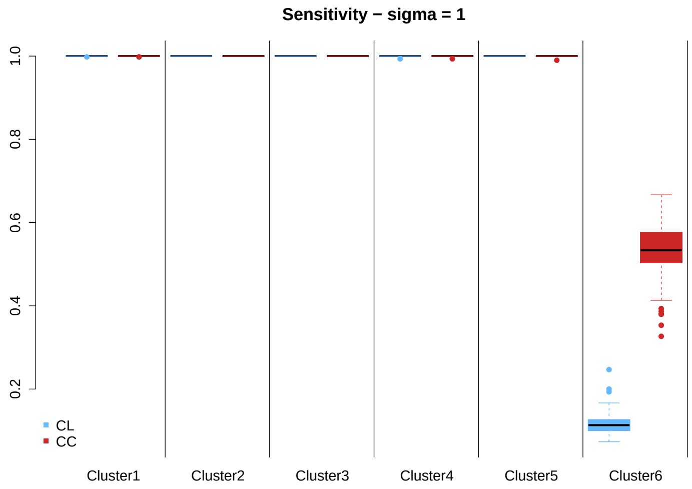

Figure S30. Boxplots of the sensitivity in each of the $K = 6$ simulated clusters for $S = 100$ simulations with CC and Complete-linkage when $\sigma = 1$. The sensitivity is the rate between the number of true positives and the sum of true positives and false negatives. In the first clusters the performance is approximately the same, while in the last cluster CC performs better.

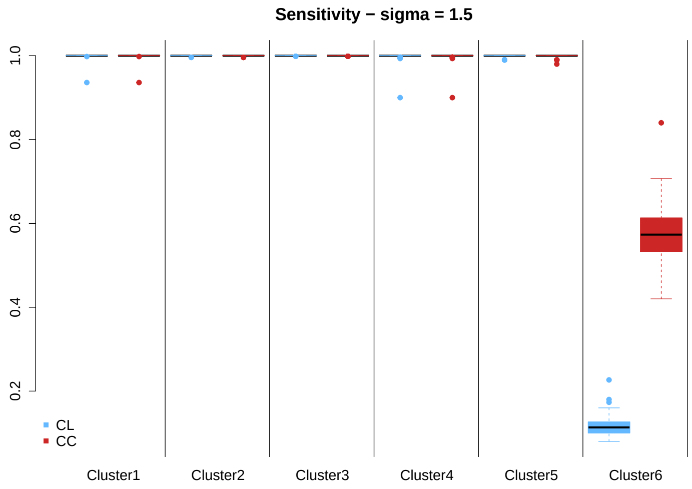

Figure S31. Boxplots of the sensitivity in each of the $K = 6$ simulated clusters for $S = 100$ simulations with CC and Complete-linkage when $\sigma = 1.5$. The sensitivity is the rate between the number of true positives and the sum of true positives and false negatives. In the first clusters the performance is approximately the same, while in the last cluster CC performs better.

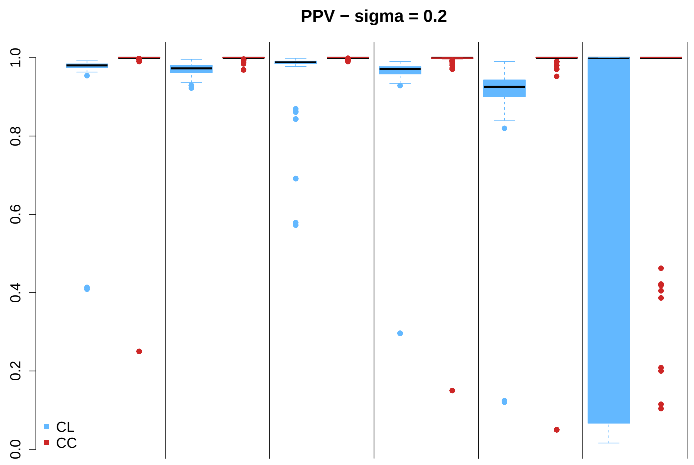

Figure S32. Boxplots of the PPV in each of the $K = 6$ simulated clusters for $S = 100$ simulations with CC and Complete-linkage when $\sigma = 0.2$. The PPV is the rate between the number of true positives and the sum of true positives and false positives. CC performs better.

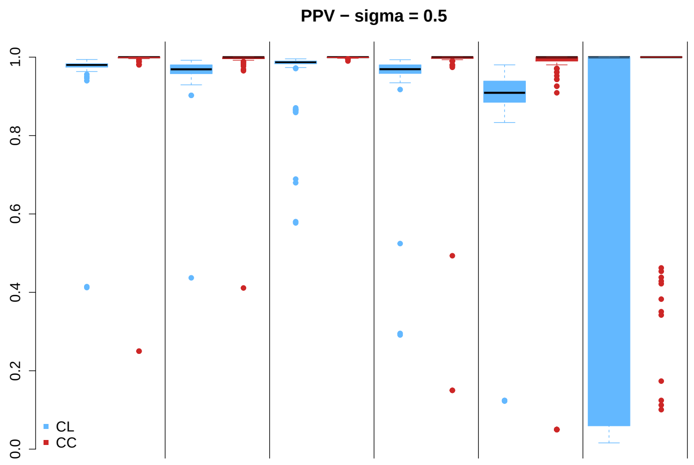

Figure S33. Boxplots of the PPV in each of the $K = 6$ simulated clusters for $S = 100$ simulations with CC and Complete-linkage when $\sigma = 0.5$. The PPV is the rate between the number of true positives and the sum of true positives and false positives. CC performs better.

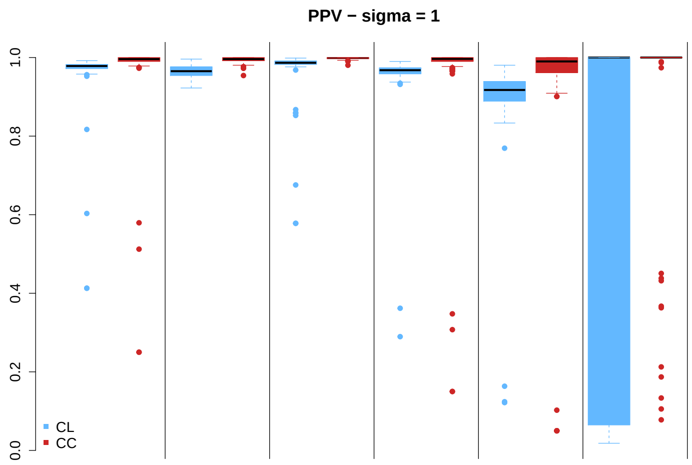

Figure S34. Boxplots of the PPV in each of the $K = 6$ simulated clusters for $S = 100$ simulations with CC and Complete-linkage when $\sigma = 1$. The PPV is the rate between the number of true positives and the sum of true positives and false positives. CC performs better.

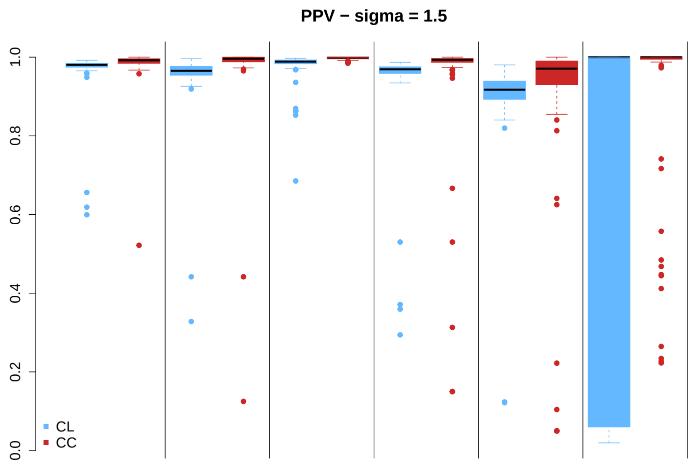

Figure S35. Boxplots of the PPV in each of the $K = 6$ simulated clusters for $S = 100$ simulations with CC and Complete-linkage when $\sigma = 1.5$. The PPV is the rate between the number of true positives and the sum of true positives and false positives. CC performs better.

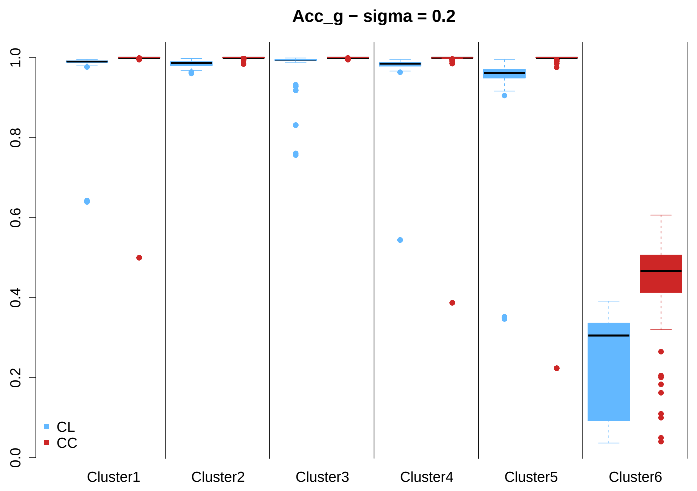

Figure S36. Boxplots of the Geometric Accuracy (Acc_g) in each of the $K = 6$ simulated clusters for $S = 100$ simulations with CC and Complete-linkage when $\sigma = 0.2$. The Geometric Accuracy is the geometric mean of sensitivity and PPV. CC performed always better.

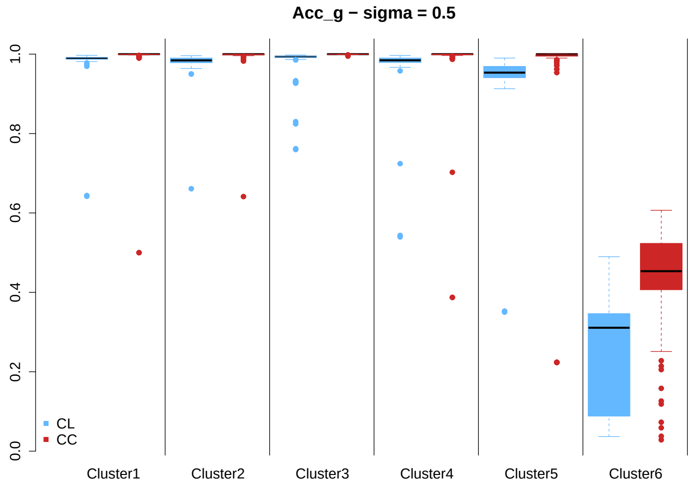

Figure S37. Boxplots of the Geometric Accuracy (Acc_g) in each of the $K = 6$ simulated clusters for $S = 100$ simulations with CC and Complete-linkage when $\sigma = 0.5$.The Geometric Accuracy is the geometric mean of sensitivity and PPV. CC performed always better.

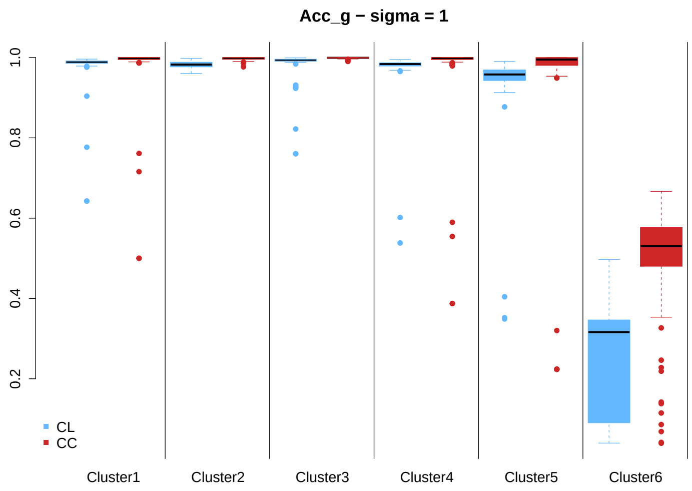

Figure S38. Boxplots of the Geometric Accuracy (Acc_g) in each of the $K = 6$ simulated clusters for $S = 100$ simulations with CC and Complete-linkage when $\sigma = 1$. The Geometric Accuracy is the geometric mean of sensitivity and PPV. CC performed always better.

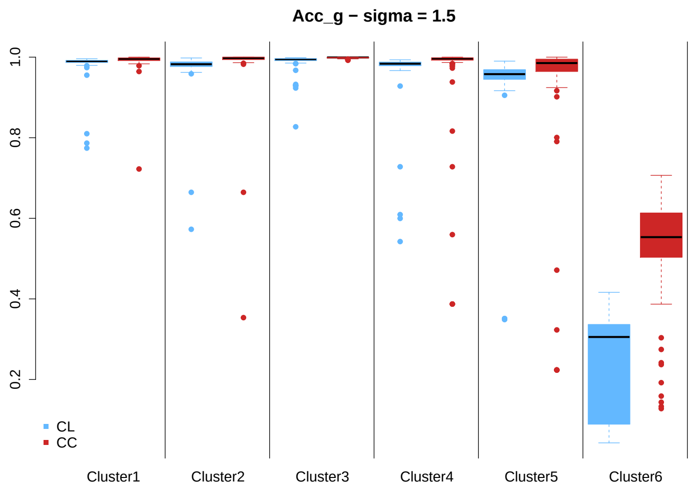

Figure S39. Boxplots of the Geometric Accuracy (Acc_g) in each of the $K = 6$ simulated clusters for $S = 100$ simulations with CC and Complete-linkage when $\sigma = 1.5$. The Geometric Accuracy is the geometric mean of sensitivity and PPV. CC performed always better.

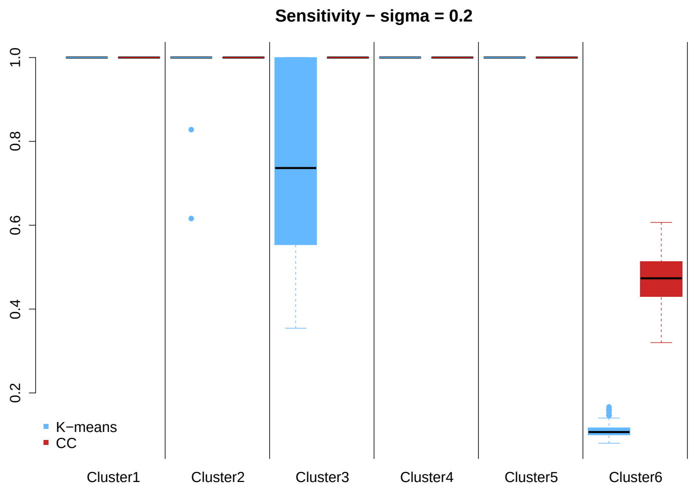

Figure S40. Boxplots of the sensitivity in each of the $K = 6$ simulated clusters for $S = 100$ simulations with CC and K-means when $\sigma = 0.2$. The sensitivity is the rate between the number of true positives and the sum of true positives and false negatives. CC performs better.

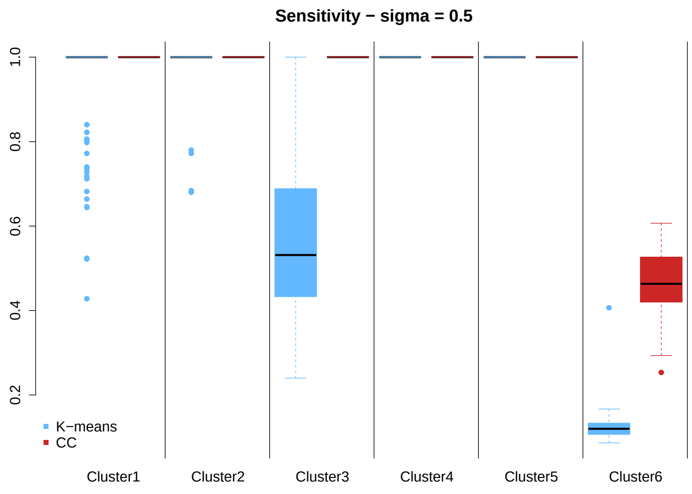

Figure S41. Boxplots of the sensitivity in each of the $K = 6$ simulated clusters for $S = 100$ simulations with CC and K-means when $\sigma = 0.5$. The sensitivity is the rate between the number of true positives and the sum of true positives and false negatives. CC performs better.

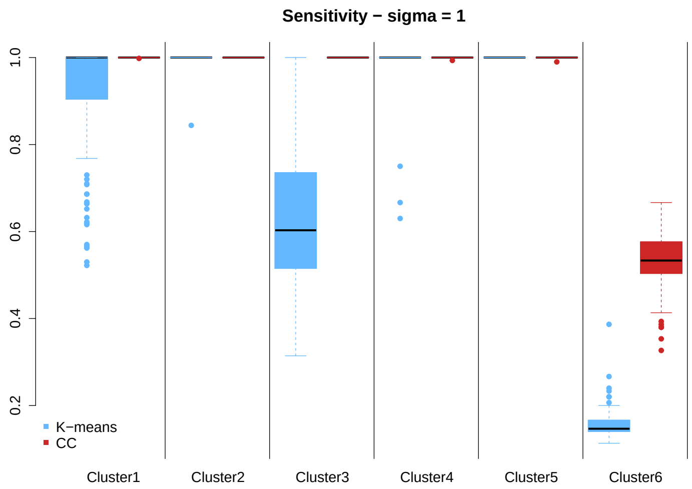

Figure S42. Boxplots of the sensitivity in each of the $K = 6$ simulated clusters for $S = 100$ simulations with CC and K-means when $\sigma = 1$. The sensitivity is the rate between the number of true positives and the sum of true positives and false negatives. CC performs better.

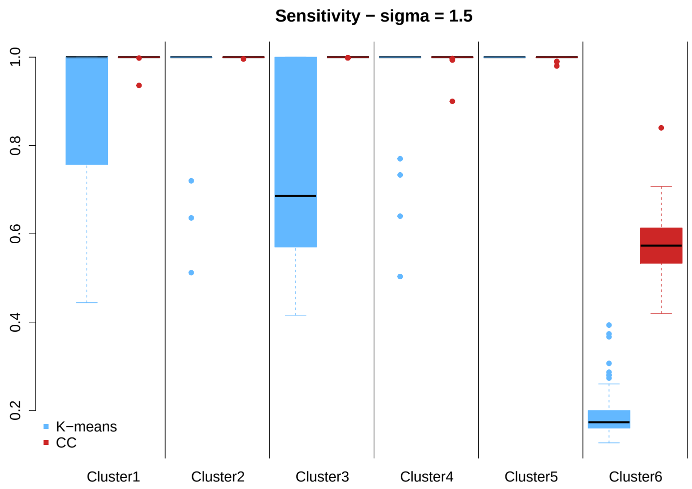

Figure S43. Boxplots of the sensitivity in each of the $K = 6$ simulated clusters for $S = 100$ simulations with CC and K-means when $\sigma = 1.5$. The sensitivity is the rate between the number of true positives and the sum of true positives and false negatives. CC performs better.

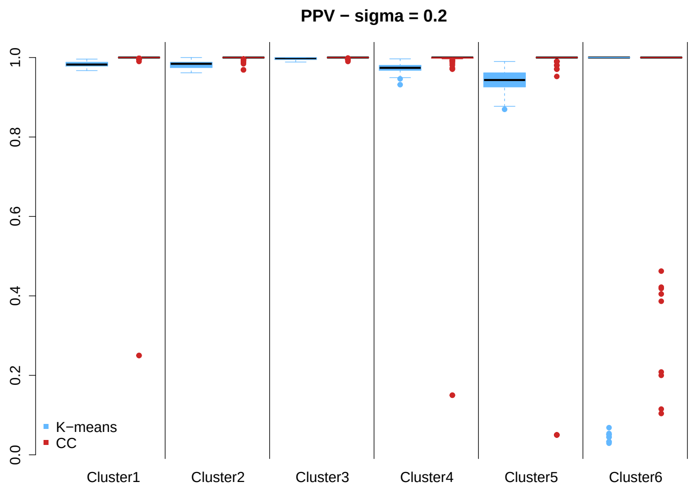

Figure S44. Boxplots of the PPV in each of the $K = 6$ simulated clusters for $S = 100$ simulations with CC and K-means when $\sigma = 0.2$. The PPV is the rate between the number of true positives and the sum of true positives and false positives. CC performs better.

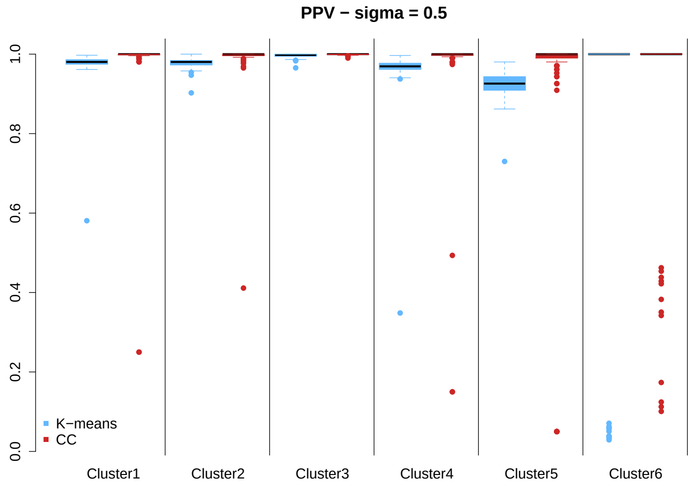

Figure S45. Boxplots of the PPV in each of the $K = 6$ simulated clusters for $S = 100$ simulations with CC and K-means when $\sigma = 0.5$. The PPV is the rate between the number of true positives and the sum of true positives and false positives. CC performs better.

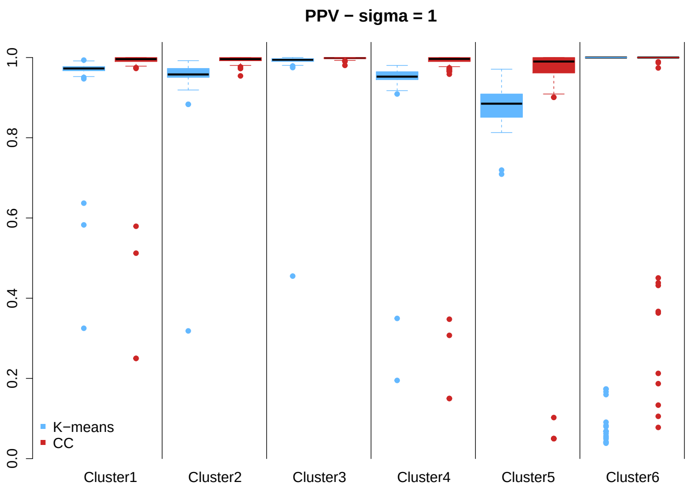

Figure S46. Boxplots of the PPV in each of the $K = 6$ simulated clusters for $S = 100$ simulations with CC and K-means when $\sigma = 1$. The PPV is the rate between the number of true positives and the sum of true positives and false positives. CC performs better.

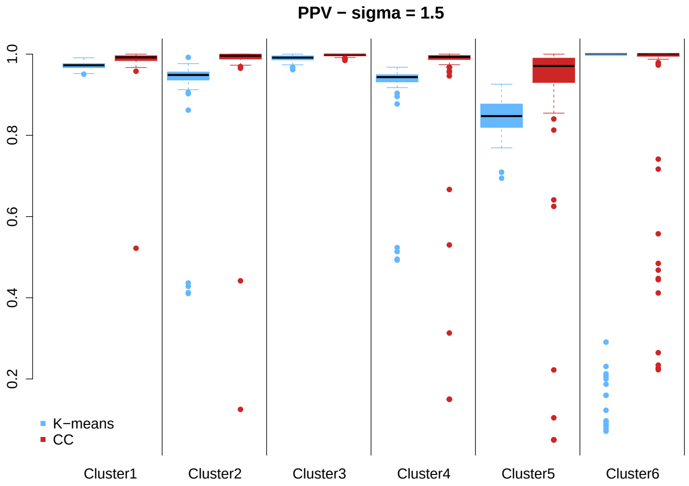

Figure S47. Boxplots of the PPV in each of the $K = 6$ simulated clusters for $S = 100$ simulations with CC and K-means when $\sigma = 1.5$. The PPV is the rate between the number of true positives and the sum of true positives and false positives. CC performs better.

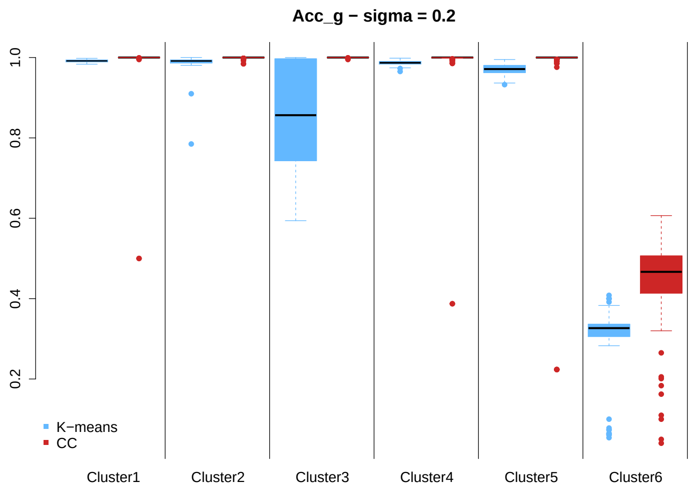

Figure S48. Boxplots of the Geometric Accuracy (Acc_g) in each of the $K = 6$ simulated clusters for $S = 100$ simulations with CC and K-means when $\sigma = 0.2$. The Geometric Accuracy is the geometric mean of sensitivity and PPV. CC performed always better.

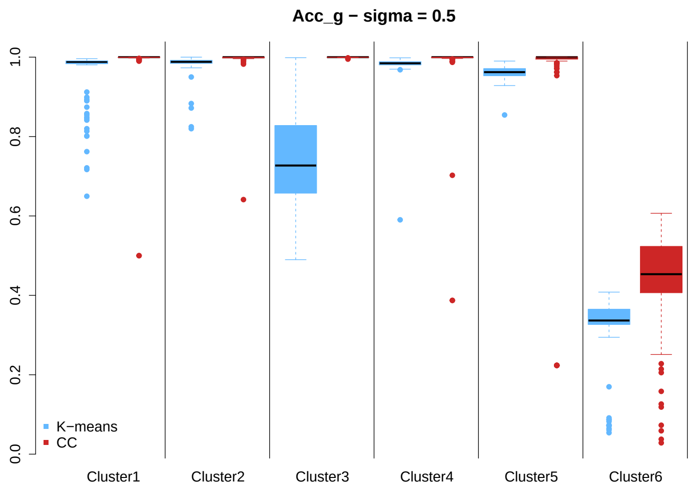

Figure S49. Boxplots of the Geometric Accuracy (Acc_g) in each of the $K = 6$ simulated clusters for $S = 100$ simulations with CC and K-means when $\sigma = 0.5$.The Geometric Accuracy is the geometric mean of sensitivity and PPV. CC performed always better.

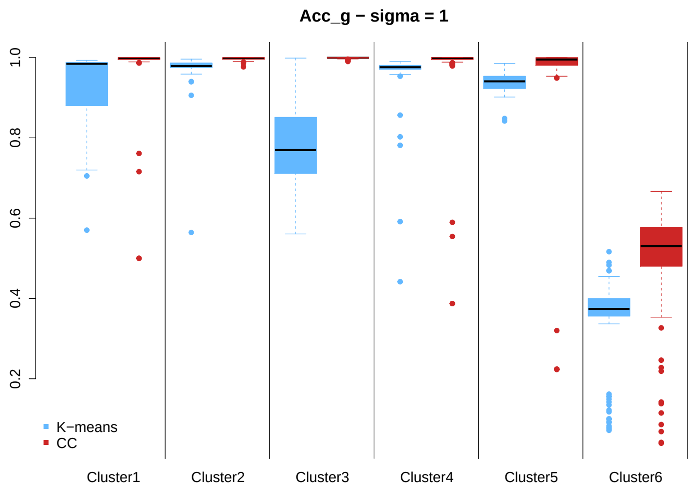

Figure S50. Boxplots of the Geometric Accuracy (Acc_g) in each of the $K = 6$ simulated clusters for $S = 100$ simulations with CC and K-means when $\sigma = 1$. The Geometric Accuracy is the geometric mean of sensitivity and PPV. CC performed always better.

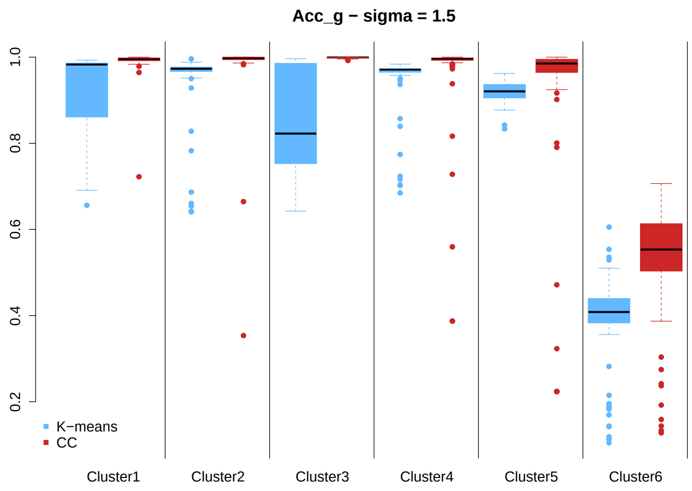

Figure S51. Boxplots of the Geometric Accuracy (Acc_g) in each of the $K = 6$ simulated clusters for $S = 100$ simulations with CC and K-means when $\sigma = 1.5$. The Geometric Accuracy is the geometric mean of sensitivity and PPV. CC performed always better.

Index	$\sigma = 0.2$				$\sigma = 0.5$				$\sigma = 1$				$\sigma = 1.5$			
	K	W	CL	CC	K	W	CL	CC	K	W	CL	CC	K	W	CL	CC
-				**100**				**100**				**100**				**100**
CH	30	100	100		28	100	100		44	100	100		55	100	100	
Sil	32	100	100		32	100	100		46	100	100		57	100	100	
Dunn	30	100	100		28	100	100		44	100	100		55	100	100	
Beale	1	100	0		1	100	6		1	100	29		6	100	47	
C-index	18	0	0		17	0	0		24	0	1		19	0	4	
Duda	4	100	0		5	100	6		8	100	29		8	100	50	
H	21	100	100		22	100	100		24	100	100		23	100	100	
KL	6	100	100		11	100	95		14	96	90		19	87	49	
Gap	21	0	37		10	0	31		11	0	30		8	0	27	
Jump	100	-	-		100	-	-		100	-	-		100	-	-	
**Average**	26.30	77.78	59.67	100	25.4	77.78	59.78	100	31.60	77.33	64.33	100	35	76.33	64.11	100
**Median**	21	100	100	100	19.5	100	95	100	24	100	90	100	21	100	50	100

Table S5. Percentages of success (identification of 5 clusters) with K-means (K), Ward's minimum variance method (W), CL and CC for $\sigma = 0.2, 0.5, 1, 1.5$ in correspondence with different indexes. Jump method is only defined for K-means. This table shows how CC was always able to detect the actual number of clusters in 100 simulations, while other methods may have some variability.

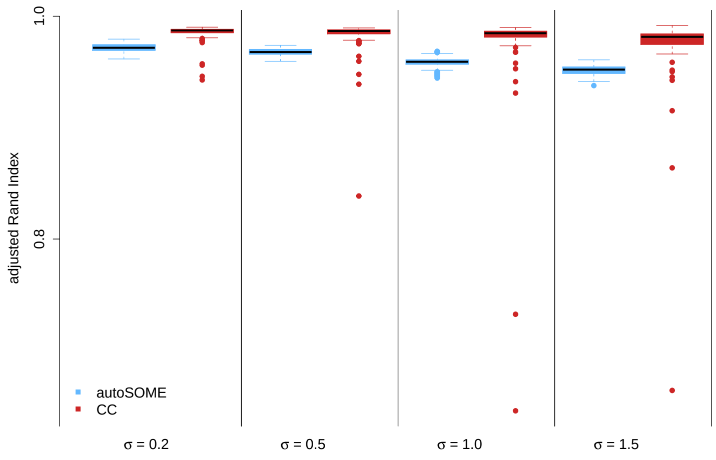

Figure S52. Boxplots of the Adjusted Rand Index (ARI) resulting from CC and autoSOME on simulated data. The ARI here is used to measure the agreement between the obtained partition with each method and the real partition, proving that CC performs always better than autoSOME in terms of ARI.

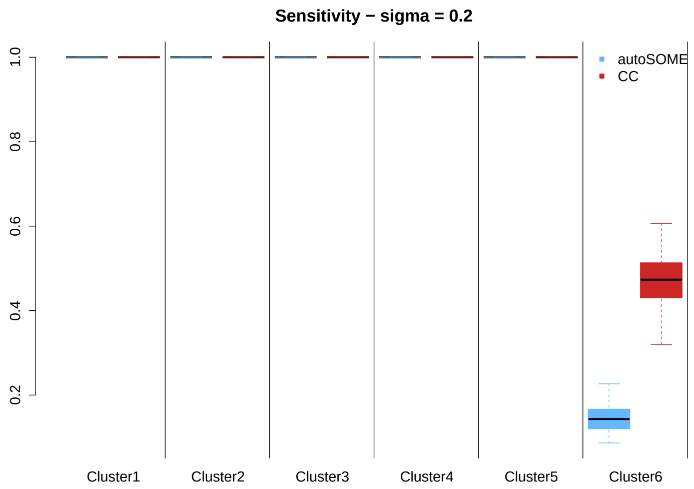

Figure S53. Boxplots of the sensitivity in each of the $K = 6$ simulated clusters for $S = 100$ simulations with CC and autoSOME when $\sigma = 0.2$. The sensitivity is the rate between the number of true positives and the sum of true positives and false negatives. In the first clusters the performance is approximately the same, while in the last group, which is the outliers one, CC performs better.

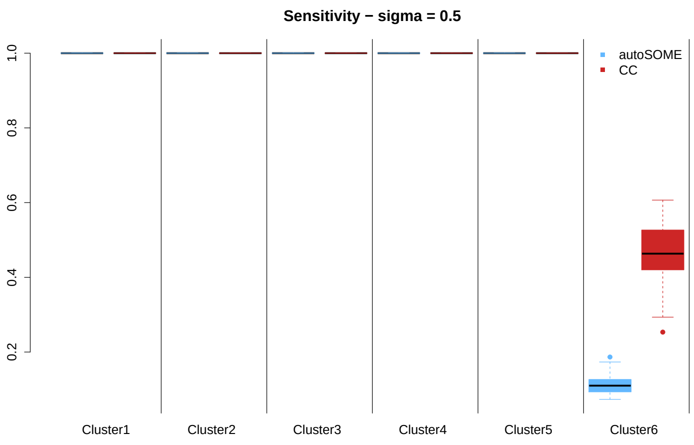

Figure S54. Boxplots of the sensitivity in each of the $K = 6$ simulated clusters for $S = 100$ simulations with CC and autoSOME when $\sigma = 0.5$. The sensitivity is the rate between the number of true positives and the sum of true positives and false negatives. In the first clusters the performance is approximately the same, while in the last group, which is the outliers one, CC performs better.

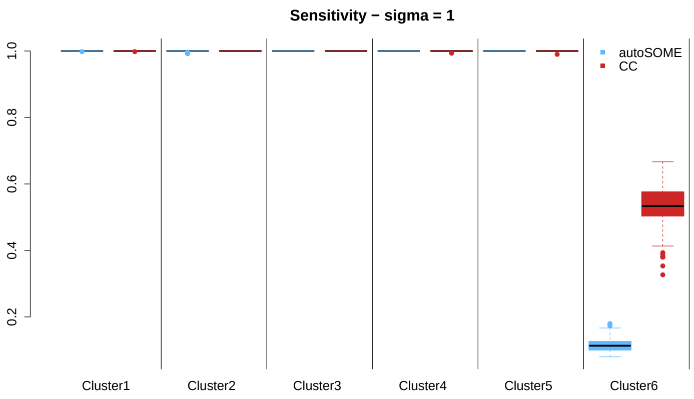

Figure S55. Boxplots of thesensitivity in each of the $K = 6$ simulated clusters for $S = 100$ simulations with CC and autoSOME when $\sigma = 1$. The sensitivity is the rate between the number of true positives and the sum of true positives and false negatives. In the first clusters the performance is approximately the same, while in the last group, which is the outliers one, CC performs better.

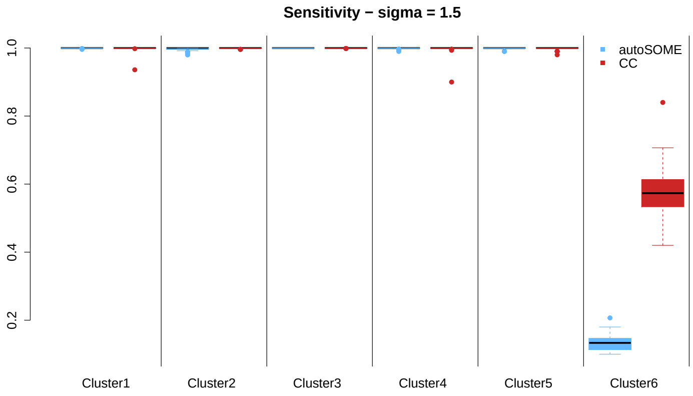

Figure S56. Boxplots of the sensitivity in each of the $K = 6$ simulated clusters for $S = 100$ simulations with CC and autoSOME when $\sigma = 1.5$. The sensitivity is the rate between the number of true positives and the sum of true positives and false negatives. In the first clusters the performance is approximately the same, while in the last group, which is the outliers one, CC performs better.

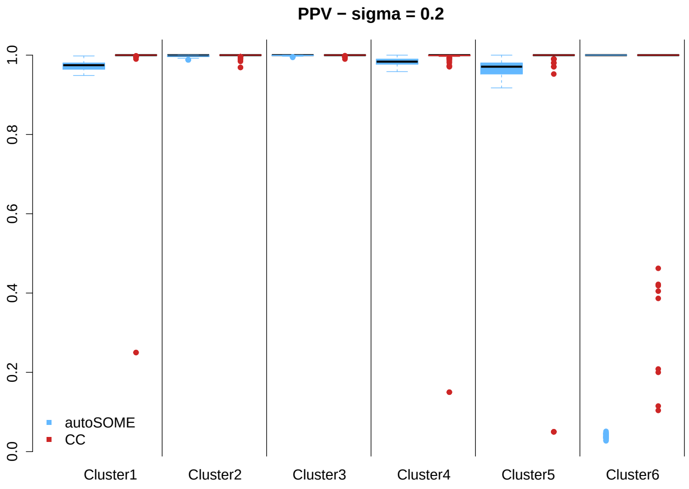

Figure S57. Boxplots of the PPV in each of the $K = 6$ simulated clusters for $S = 100$ simulations with CC and autoSOME when $\sigma = 0.2$. The PPV is the rate between the number of true positives and the sum of true positives and false positives. The performance is approximately the same.

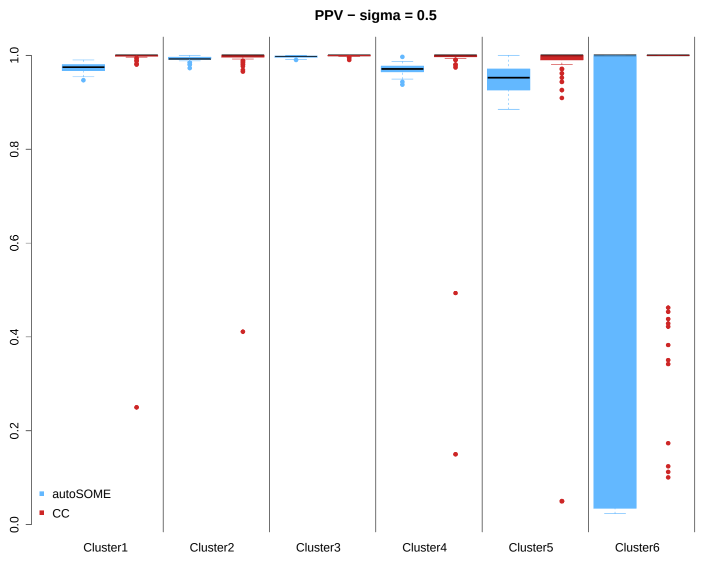

Figure S58. Boxplots of the PPV in each of the $K = 6$ simulated clusters for $S = 100$ simulations with CC and autoSOME when $\sigma = 0.5$. The PPV is the rate between the number of true positives and the sum of true positives and false positives. In the first clusters the performance is approximately the same, while in the last group, which is the outliers one, CC performs better.

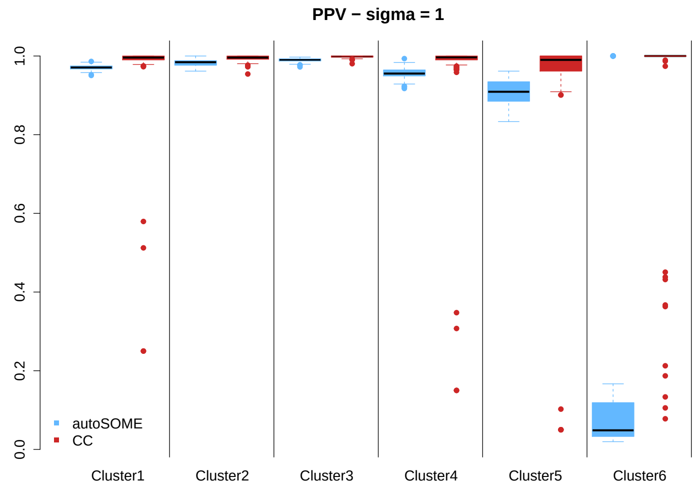

Figure S59. Boxplots of the PPV in each of the $K = 6$ simulated clusters for $S = 100$ simulations with CC and autoSOME when $\sigma = 1$. The PPV is the rate between the number of true positives and the sum of true positives and false positives. In the first clusters the performance is approximately the same, while in the last group, which is the outliers one, CC performs better.

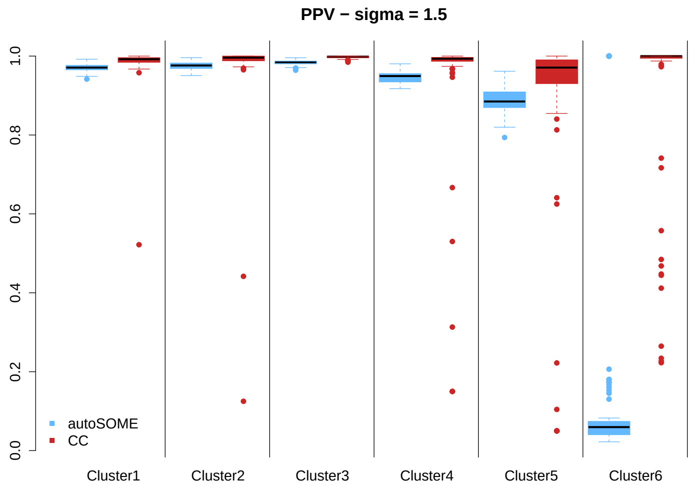

Figure S60. Boxplots of the PPV in each of the $K = 6$ simulated clusters for $S = 100$ simulations with CC and autoSOME when $\sigma = 1.5$. The PPV is the rate between the number of true positives and the sum of true positives and false positives. In the first clusters the performance is approximately the same, while in the last group, which is the outliers one, CC performs better.

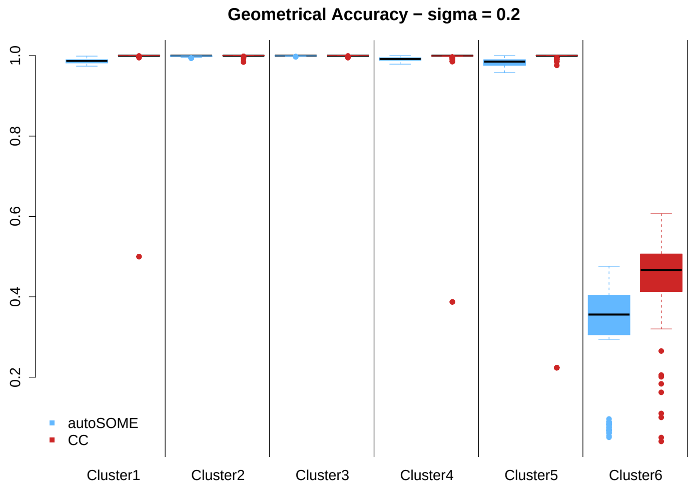

Figure S61. Boxplots of the geometric accuracy in each of the $K = 6$ simulated clusters for $S = 100$ simulations with CC and autoSOME when $\sigma = 0.2$. The geometric accuracy is the geometric mean of sensitivity and PPV. In the first clusters the performance is approximately the same, while in the last group, which is the outliers one, CC performs better.

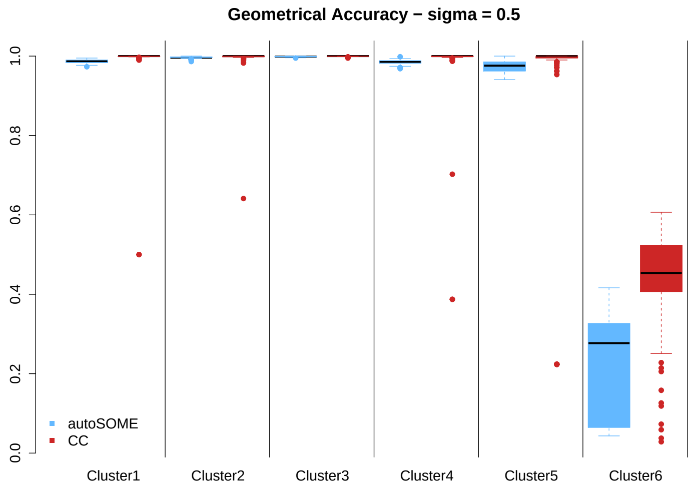

Figure S62. Boxplots of the geometric accuracy in each of the $K = 6$ simulated clusters for $S = 100$ simulations with CC and autoSOME when $\sigma = 0.5$.The geometric accuracy is the geometric mean of sensitivity and PPV. In the first clusters the performance is approximately the same, while in the last group, which is the outliers one, CC performs better.

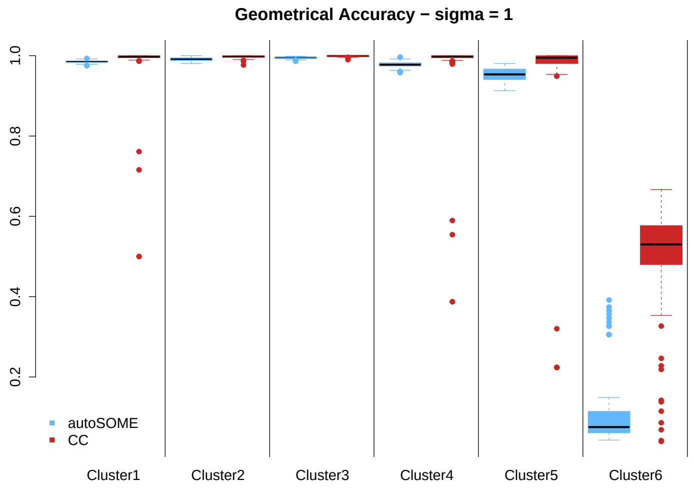

Figure S63. Boxplots of the geometric accuracy in each of the $K = 6$ simulated clusters for $S = 100$ simulations with CC and autoSOME when $\sigma = 1$. The geometric accuracy is the geometric mean of sensitivity and PPV. In the first clusters the performance is approximately the same, while in the last group, which is the outliers one, CC performs better.

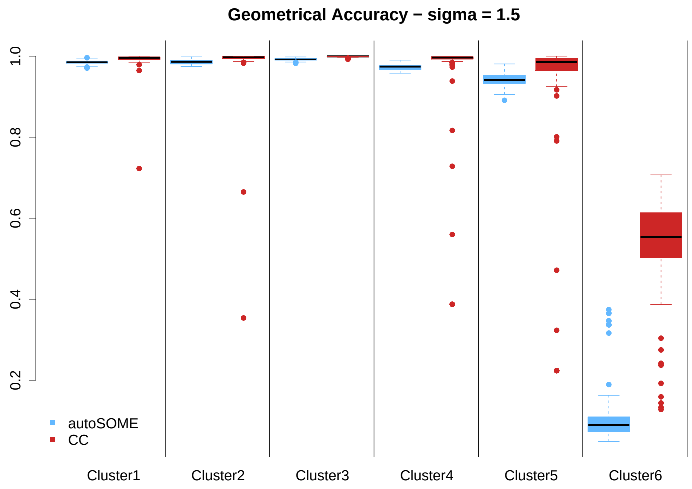

Figure S64. Boxplots of the geometric accuracy in each of the $K = 6$ simulated clusters for $S = 100$ simulations with CC and autoSOME when $\sigma = 1.5$. The geometric accuracy is the geometric mean of sensitivity and PPV. In the first clusters the performance is approximately the same, while in the last group, which is the outliers one, CC performs better.

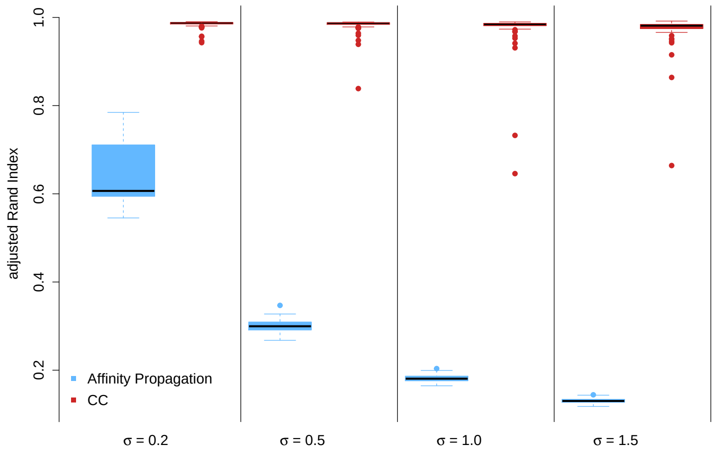

Figure S65. Boxplots of the Adjusted Rand Index (ARI) resulting from CC and Affinity Propagation on simulated data. The ARI here is used to measure the agreement between the obtained partition with each method and the real partition, proving that CC performs always better than Affinity Propagation in terms of ARI.

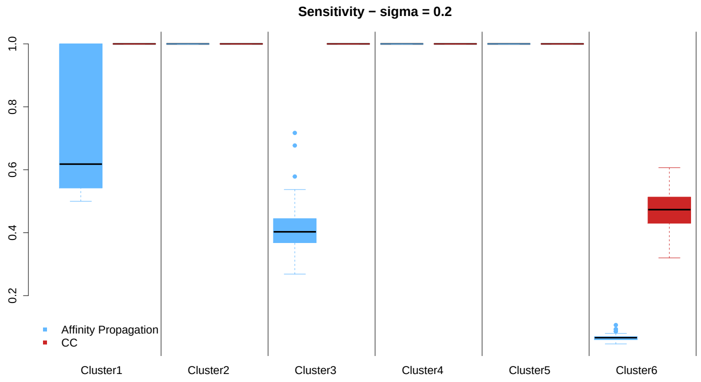

Figure S66. Boxplots of the sensitivity in each of the $K = 6$ simulated clusters for $S = 100$ simulations with CC and Affinity Propagation when $\sigma = 0.2$. The sensitivity is the rate between the number of true positivse and the sum of true positives and false negatives. In some clusters the performance is approximately the same, while in the other groups CC performs better.

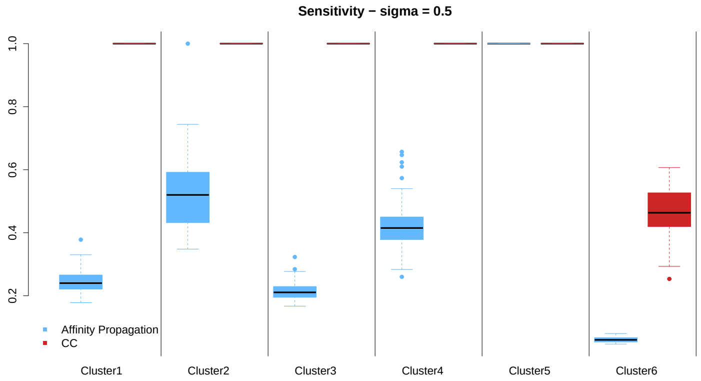

Figure S67. Boxplots of the sensitivity in each of the $K = 6$ simulated clusters for $S = 100$ simulations with CC and Affinity Propagation when $\sigma = 0.5$. The sensitivity is the rate between the number of true positives and the sum of true positives and false negatives. CC performs always better.

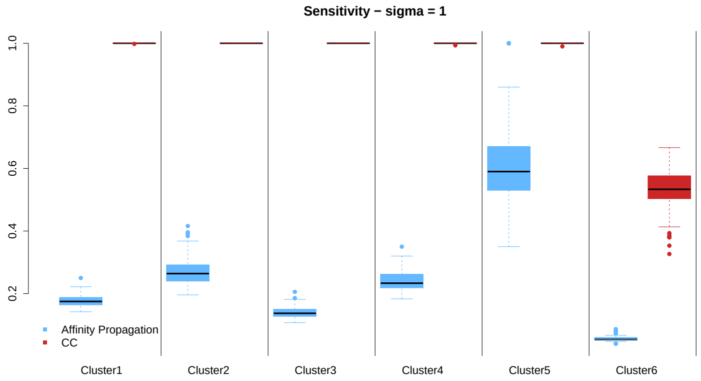

Figure S68. Boxplots of the xensitivity in each of the $K = 6$ simulated clusters for $S = 100$ simulations with CC and Affinity Propagation when $\sigma = 1$. The sensitivity is the rate between the number of true positives and the sum of true positives and false negatives. CC performs always better.

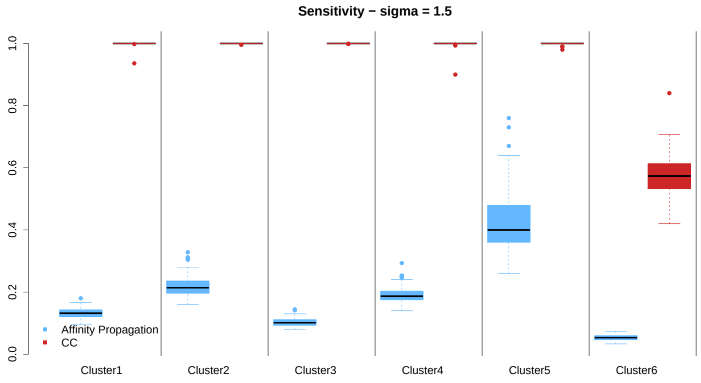

Figure S69. Boxplots of the xensitivity in each of the $K = 6$ simulated clusters for $S = 100$ simulations with CC and Affinity Propagation when $\sigma = 1.5$. The sensitivity is the rate between the number of true positives and the sum of true positives and false negatives. CC performs always better.

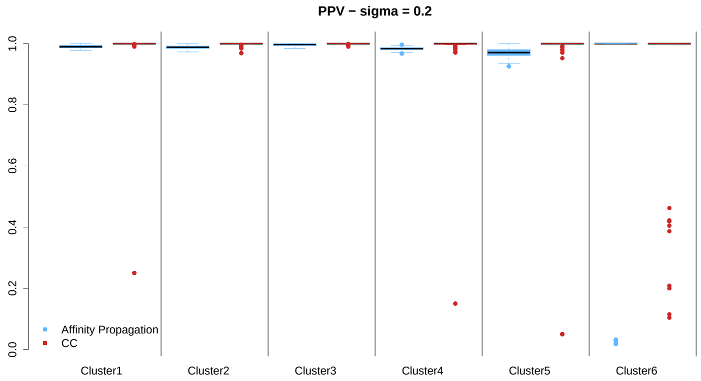

Figure S70. Boxplots of the PPV in each of the $K = 6$ simulated clusters for $S = 100$ simulations with CC and Affinity Propagation when $\sigma = 0.2$. The PPV is the rate between the number of true positives and the sum of true positivse and false positives. The performance is approximately the same.

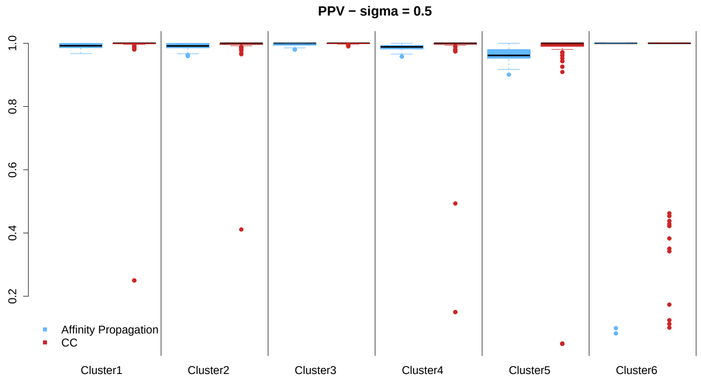

Figure S71. Boxplots of the PPV in each of the $K = 6$ simulated clusters for $S = 100$ simulations with CC and Affinity Propagation when $\sigma = 0.5$. The PPV is the rate between the number of true positives and the sum of true positivse and false positives. The performance is approximately the same.

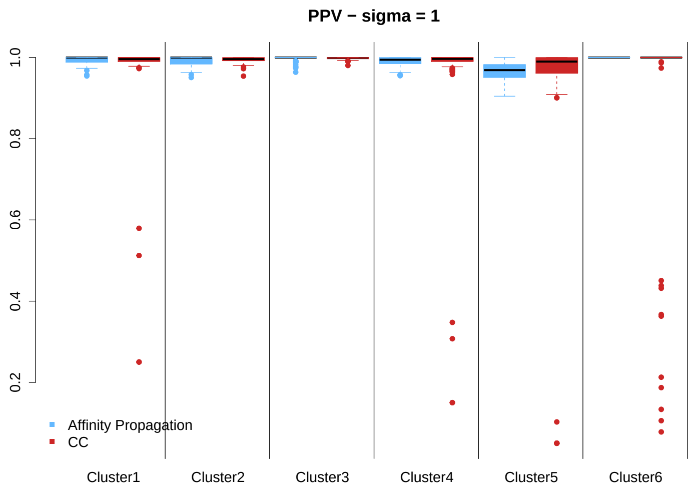

Figure S72. Boxplots of the PPV in each of the $K = 6$ simulated clusters for $S = 100$ simulations with CC and Affinity Propagation when $\sigma = 1$. The PPV is the rate between the number of true positives and the sum of true positivse and false positives.The performance is approximately the same.

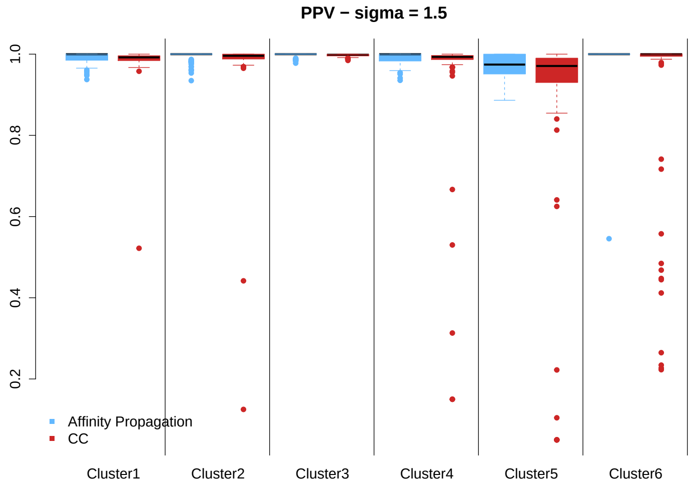

Figure S73. Boxplots of the PPV in each of the $K = 6$ simulated clusters for $S = 100$ simulations with CC and Affinity Propagation when $\sigma = 1.5$. The PPV is the rate between the number of true positives and the sum of true positivse and false positives. The performance is approximately the same.

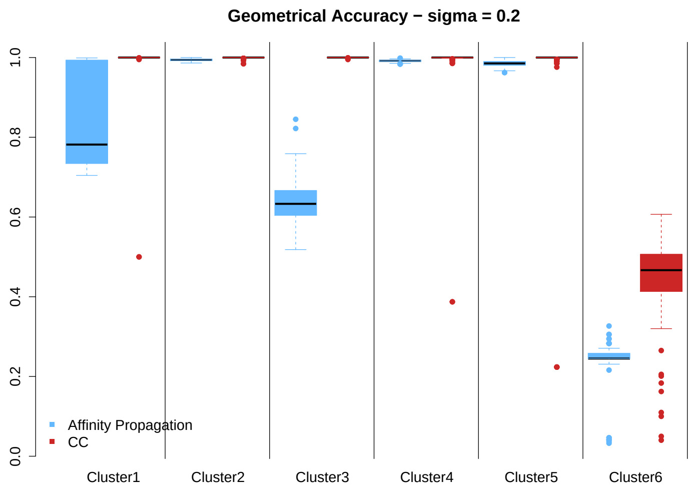

Figure S74. Boxplots of the geometric accuracy in each of the $K = 6$ simulated clusters for $S = 100$ simulations with CC and Affinity Propagation when $\sigma = 0.2$. The geometric accuracy is the geometric mean of sensitivity and PPV. In some clusters the performance is approximately the same, while in other groups CC performs better.

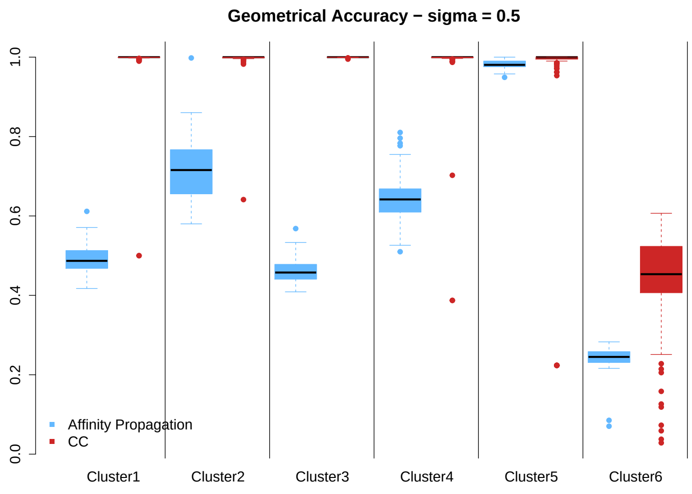

Figure S75. Boxplots of the geometric accuracy in each of the $K = 6$ simulated clusters for $S = 100$ simulations with CC and Affinity Propagation when $\sigma = 0.5$.The geometric accuracy is the geometric mean of sensitivity and PPV. CC performs always better.

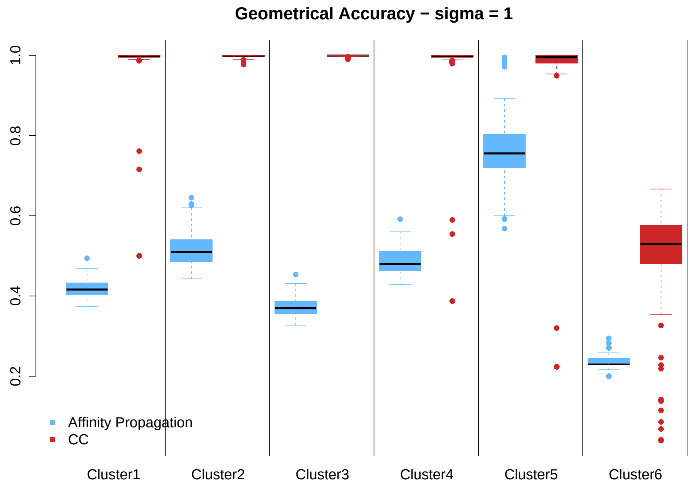

Figure S76. Boxplots of the geometric accuracy in each of the $K = 6$ simulated clusters for $S = 100$ simulations with CC and Affinity Propagation when $\sigma = 1$. The geometric accuracy is the geometric mean of sensitivity and PPV. CC performs always better.

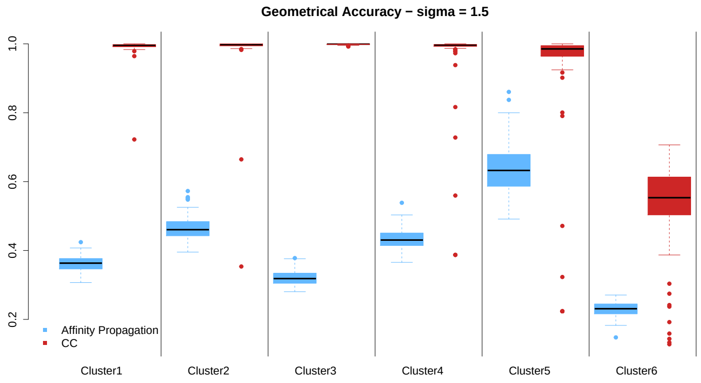

Figure S77. Boxplots of the geometric accuracy in each of the $K = 6$ simulated clusters for $S = 100$ simulations with CC and Affinity Propagation when $\sigma = 1.5$. The geometric accuracy is the geometric mean of sensitivity and PPV. CC performs always better.

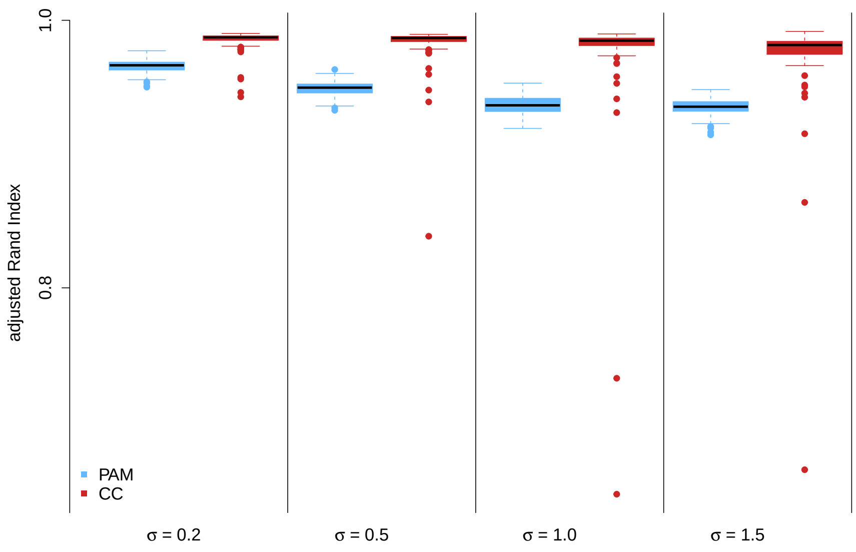

Figure S78. Boxplots of the Adjusted Rand Index (ARI) resulting from CC and PAM on simulated data. The ARI here is used to measure the agreement between the obtained partition with each method and the real partition, proving that CC performs always better than PAM in terms of ARI.

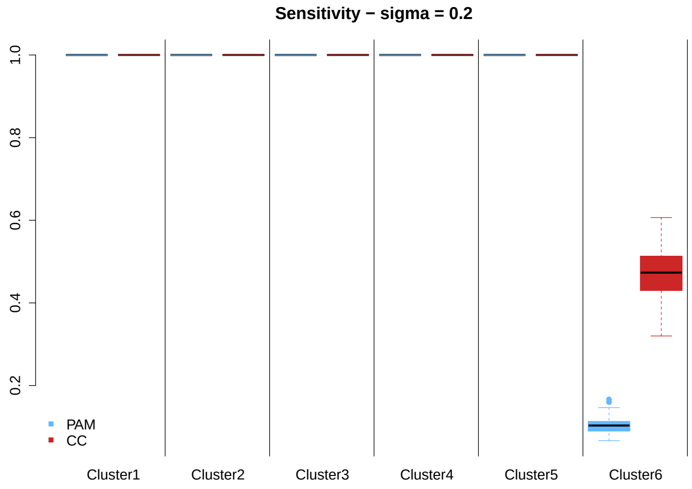

Figure S79. Boxplots of the sensitivity in each of the $K = 6$ simulated clusters for $S = 100$ simulations with CC and PAM when $\sigma = 0.2$. The sensitivity is the rate between the number of true positivse and the sum of true positives and false negatives. In the first clusters the performance is approximately the same, while in the last cluster CC performs better.

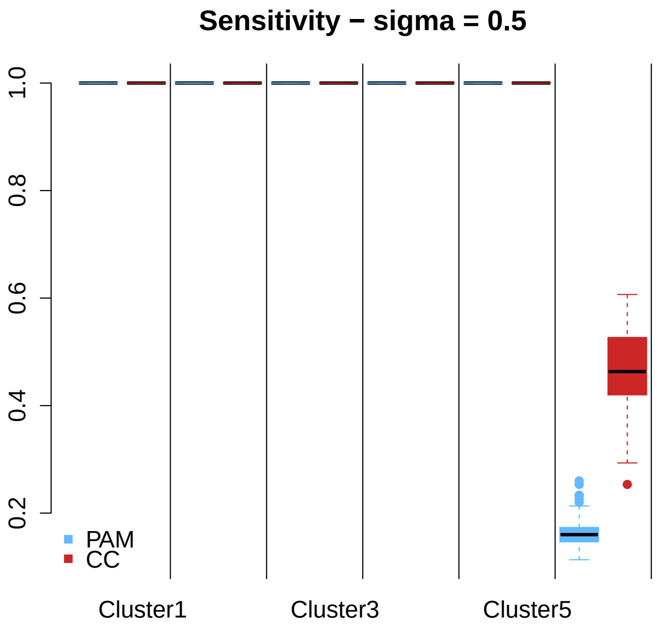

Figure S80. Boxplots of the sensitivity in each of the $K = 6$ simulated clusters for $S = 100$ simulations with CC and PAM when $\sigma = 0.5$. The sensitivity is the rate between the number of true positives and the sum of true positives and false negatives. In the first clusters the performance is approximately the same, while in the last cluster CC performs better.

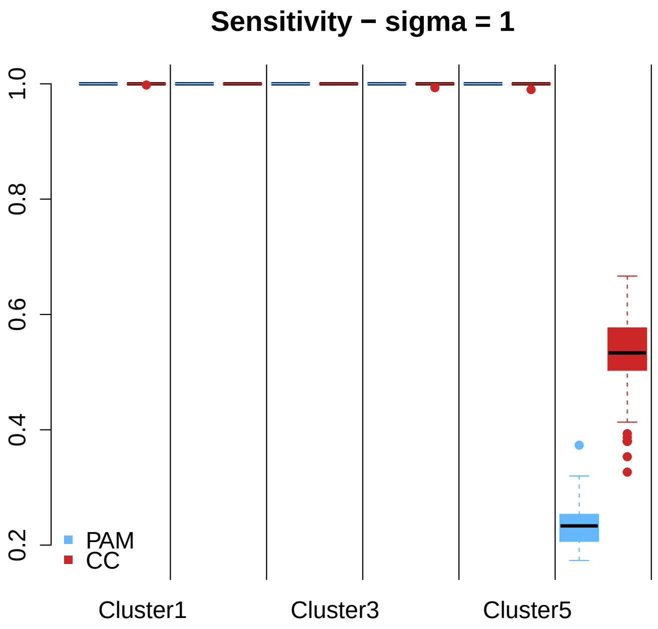

Figure S81. Boxplots of the sensitivity in each of the $K = 6$ simulated clusters for $S = 100$ simulations with CC and PAM when $\sigma = 1$. The sensitivity is the rate between the number of true positives and the sum of true positives and false negatives. In the first clusters the performance is approximately the same, while in the last cluster CC performs better.

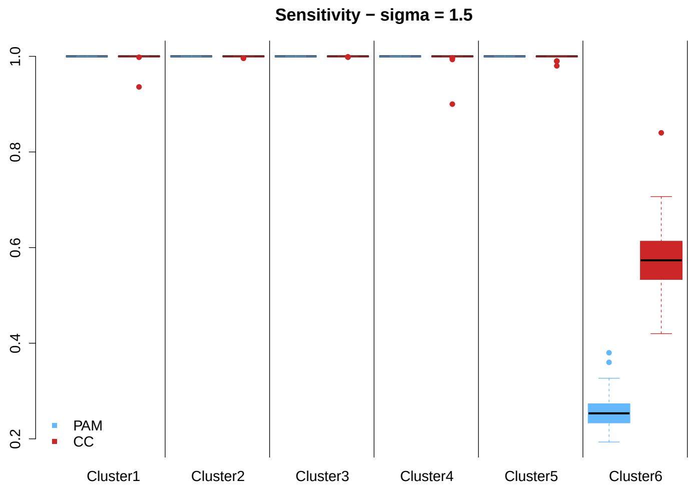

Figure S82. Boxplots of the sensitivity in each of the $K = 6$ simulated clusters for $S = 100$ simulations with CC and PAM when $\sigma = 1.5$. The sensitivity is the rate between the number of true positives and the sum of true positives and false negatives. In the first clusters the performance is approximately the same, while in the last cluster CC performs better.

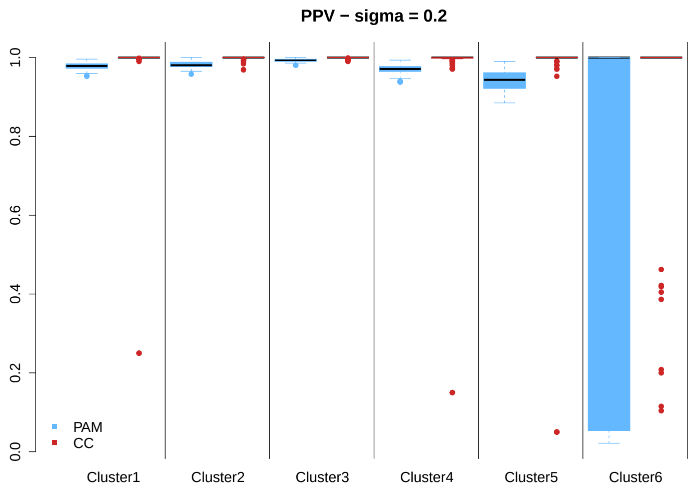

Figure S83. Boxplots of the PPV in each of the $K = 6$ simulated clusters for $S = 100$ simulations with CC and PAM when $\sigma = 0.2$. The PPV is the rate between the number of true positives and the sum of true positives and false positives. CC performed always better.

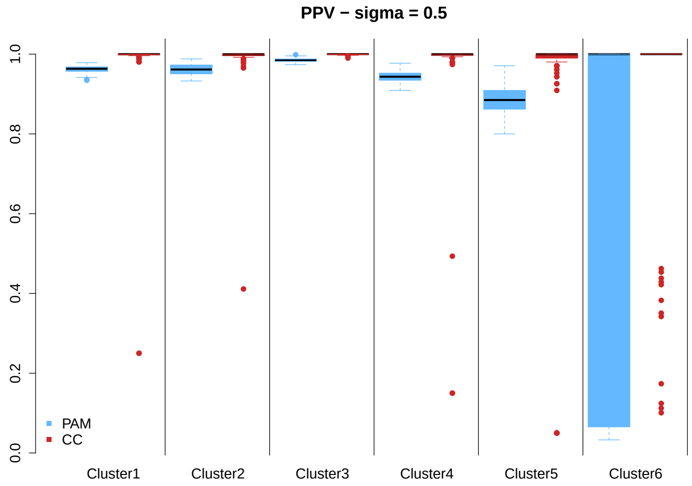

Figure S84. Boxplots of the PPV in each of the $K = 6$ simulated clusters for $S = 100$ simulations with CC and PAM when $\sigma = 0.5$. The PPV is the rate between the number of true positives and the sum of true positives and false positives. CC performed always better.

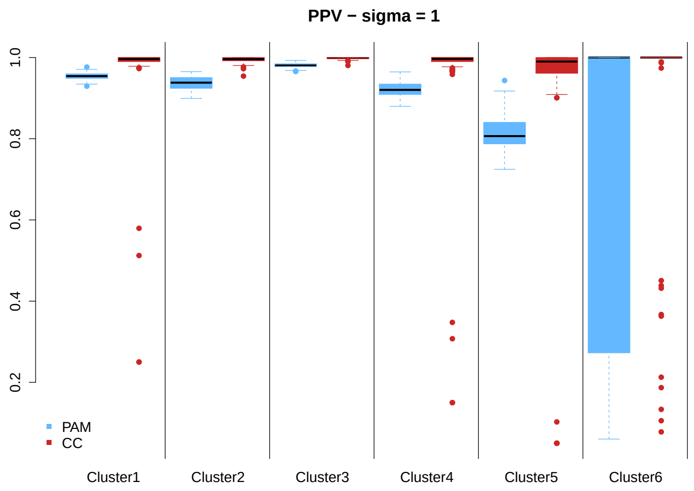

Figure S85. Boxplots of the PPV in each of the $K = 6$ simulated clusters for $S = 100$ simulations with CC and PAM when $\sigma = 1$. The PPV is the rate between the number of true positives and the sum of true positives and false positives. CC performed always better.

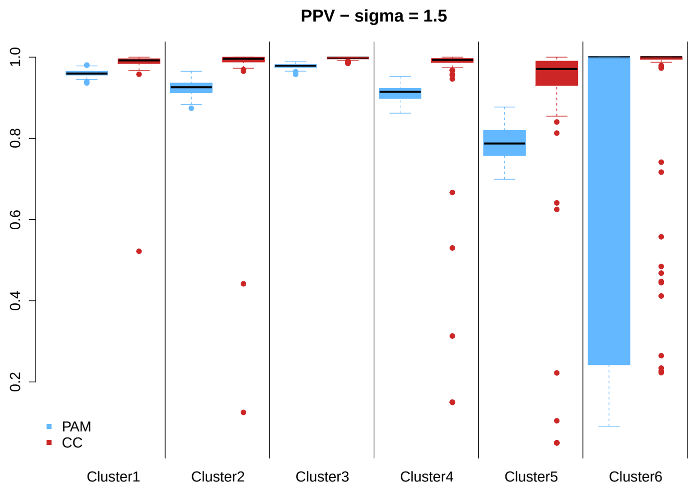

Figure S86. Boxplots of the PPV in each of the $K = 6$ simulated clusters for $S = 100$ simulations with CC and PAM when $\sigma = 1.5$. The PPV is the rate between the number of true positives and the sum of true positives and false positives. CC performed always better.

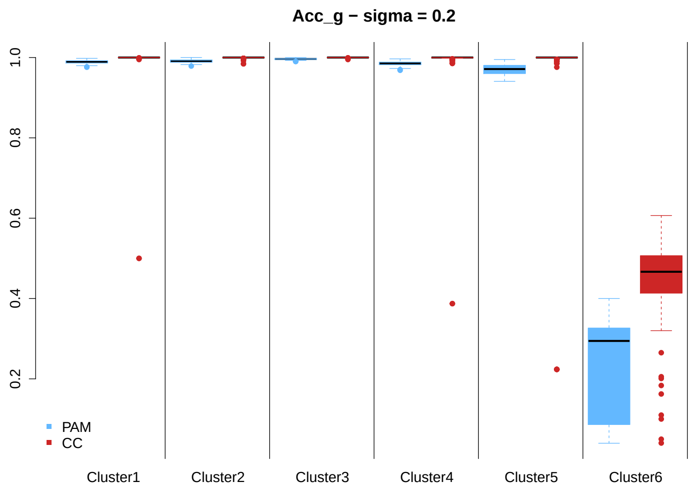

Figure S87. Boxplots of the geometric accuracy (Acc_g) in each of the $K = 6$ simulated clusters for $S = 100$ simulations with CC and PAM when $\sigma = 0.2$. The geometric accuracy is the geometric mean of sensitivity and PPV. In the first clusters the performance is approximately the same, while in last cluster CC performs better.

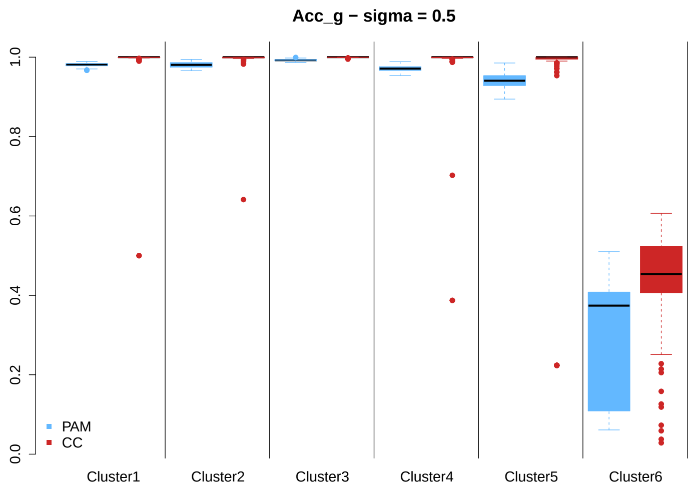

Figure S88. Boxplots of the geometric accuracy (Acc_g) in each of the $K = 6$ simulated clusters for $S = 100$ simulations with CC and PAM when $\sigma = 0.5$.The Geometric Accuracy is the geometric mean of sensitivity and PPV. In the first clusters the performance is approximately the same, while in last cluster CC performs better.

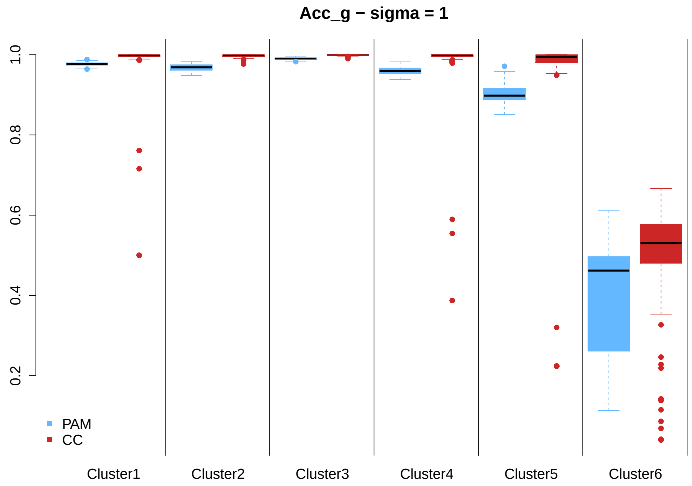

Figure S89. Boxplots of the geometric accuracy (Acc_g) in each of the $K = 6$ simulated clusters for $S = 100$ simulations with CC and PAM when $\sigma = 1$. The Geometric Accuracy is the geometric mean of sensitivity and PPV. In the first clusters the performance is approximately the same, while in last cluster CC performs better.

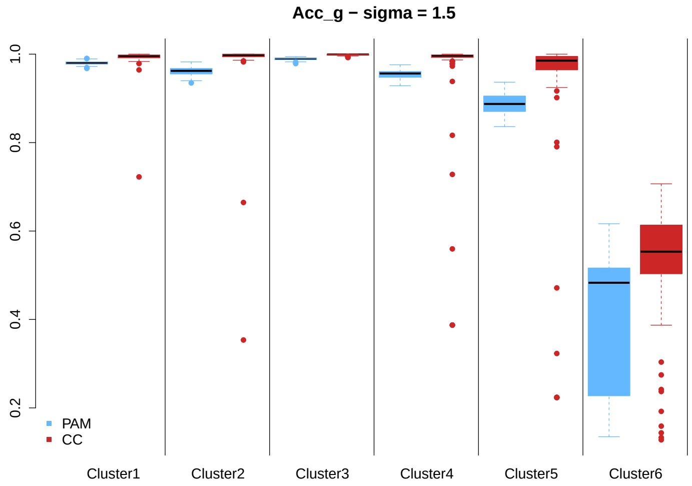

Figure S90. Boxplots of the geometric accuracy (Acc_g) in each of the $K = 6$ simulated clusters for $S = 100$ simulations with CC and PAM when $\sigma = 1.5$. The Geometric Accuracy is the geometric mean of sensitivity and PPV. In the first clusters the performance is approximately the same, while in last cluster CC performs better.

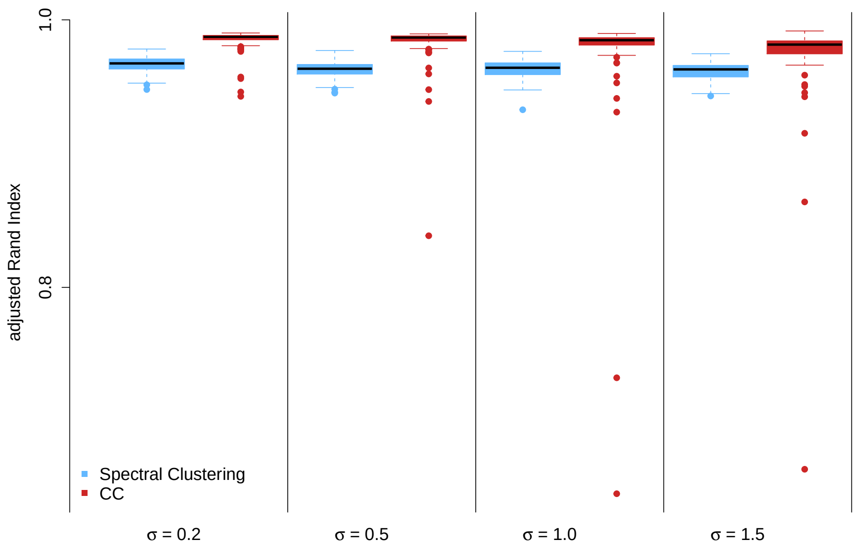

Figure S91. Boxplots of the Adjusted Rand Index (ARI) resulting from CC and Spectral clustering on simulated data. The ARI here is used to measure the agreement between the obtained partition with each method and the real partition, proving that CC performs always better than Spectral clustering in terms of ARI.

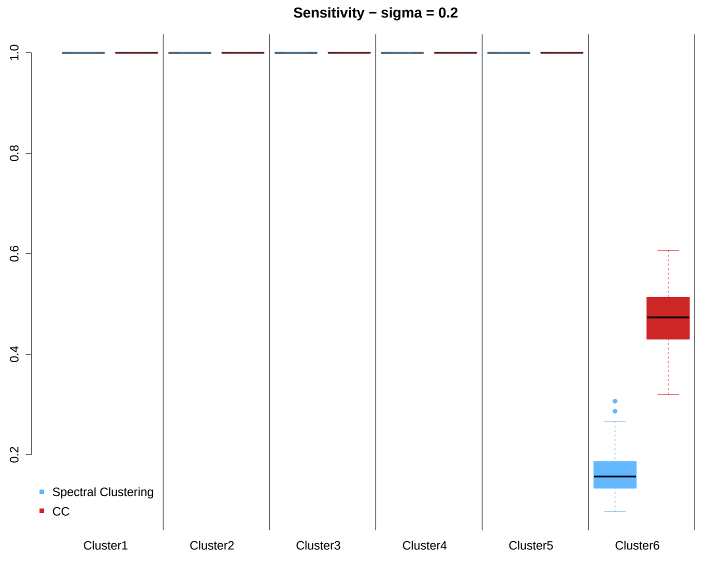

Figure S92. Boxplots of the sensitivity in each of the $K = 6$ simulated clusters for $S = 100$ simulations with CC and Spectral clustering when $\sigma = 0.2$. The sensitivity is the rate between the number of true positives and the sum of true positives and false negatives. In the first clusters the performance is approximately the same, while in the last cluster CC performs better.

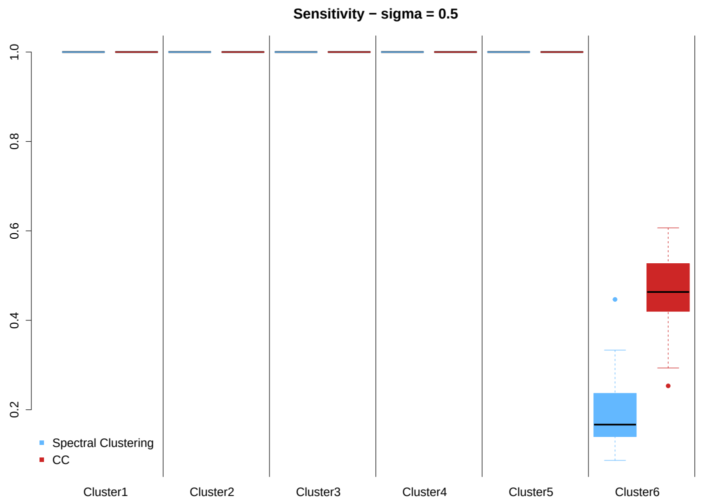

Figure S93. Boxplots of the sensitivity in each of the $K = 6$ simulated clusters for $S = 100$ simulations with CC and Spectral clustering when $\sigma = 0.5$. The sensitivity is the rate between the number of true positives and the sum of true positives and false negatives. In the first clusters the performance is approximately the same, while in the last cluster CC performs better.

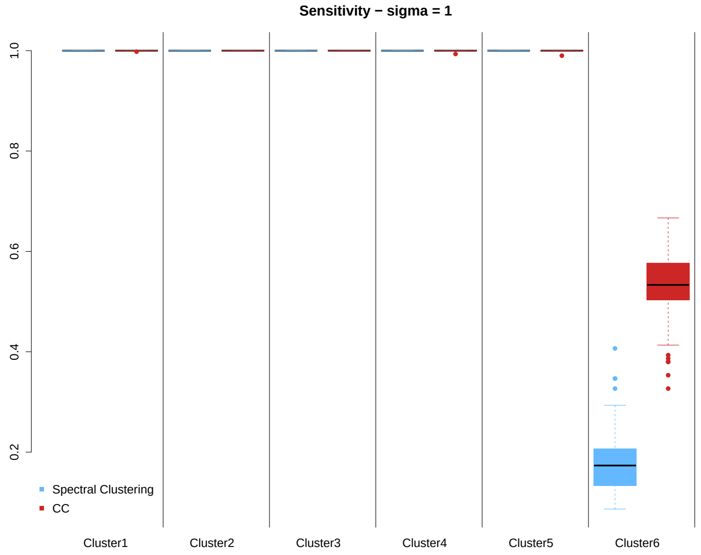

Figure S94. Boxplots of the Sensitivity in each of the $K = 6$ simulated clusters for $S = 100$ simulations with CC and Spectral clustering when $\sigma = 1$. The sensitivity is the rate between the number of true positives and the sum of true positives and false negatives. In the first clusters the performance is approximately the same, while in the last cluster CC performs better.

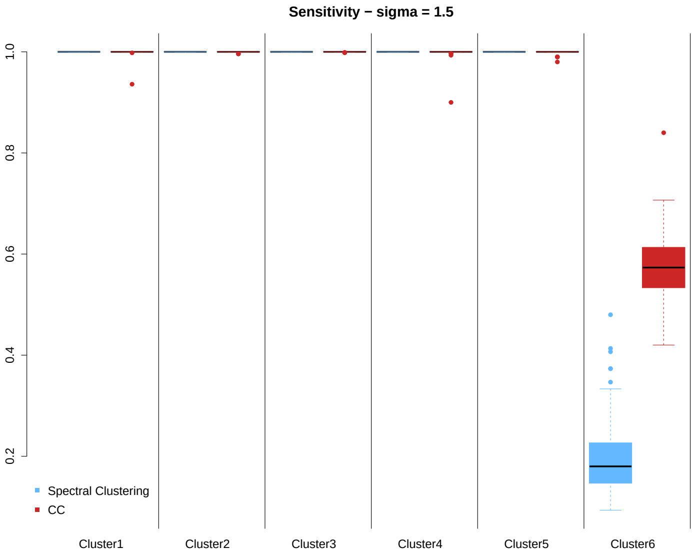

Figure S95. Boxplots of the Sensitivity in each of the $K = 6$ simulated clusters for $S = 100$ simulations with CC and Spectral clustering when $\sigma = 1.5$. The sensitivity is the rate between the number of true positives and the sum of true positives and false negatives. In the first clusters the performance is approximately the same, while in the last cluster CC performs better.

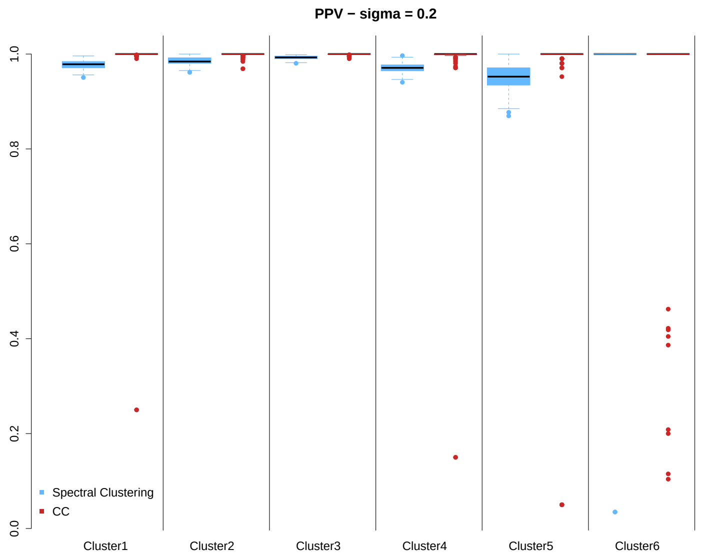

Figure S96. Boxplots of the PPV in each of the $K = 6$ simulated clusters for $S = 100$ simulations with CC and Spectral clustering when $\sigma = 0.2$. The PPV is the rate between the number of true positives and the sum of true positives and false positives. CC performed better on the first clusters, while the performance is approximately the same in the last cluster.

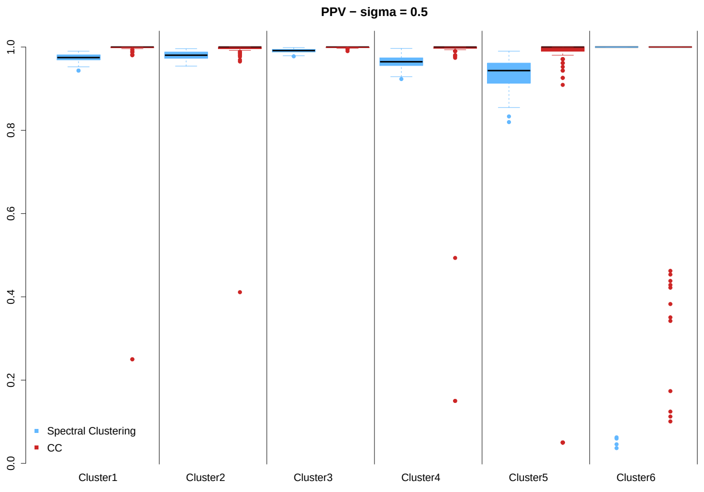

Figure S97. Boxplots of the PPV in each of the $K = 6$ simulated clusters for $S = 100$ simulations with CC and Spectral clustering when $\sigma = 0.5$. The PPV is the rate between the number of true positives and the sum of true positives and false positives. CC performed better on the first clusters, while the performance is approximately the same in the last cluster.

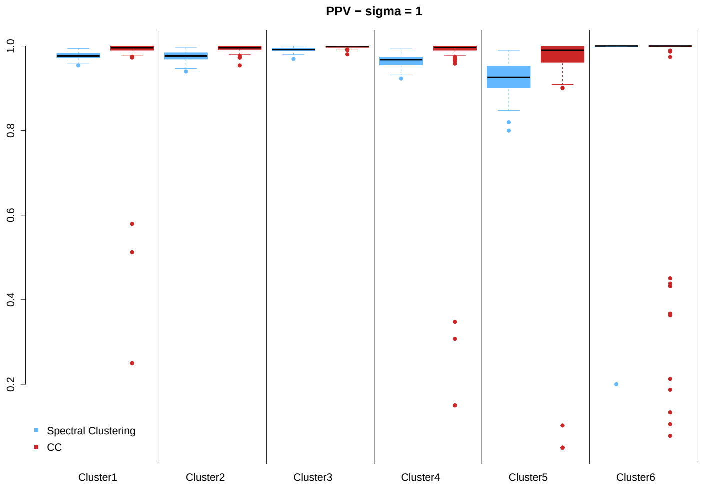

Figure S98. Boxplots of the PPV in each of the $K = 6$ simulated clusters for $S = 100$ simulations with CC and Spectral clustering when $\sigma = 1$. The PPV is the rate between the number of true positives and the sum of true positives and false positives. CC performed better on the first clusters, while the performance is approximately the same in the last cluster.

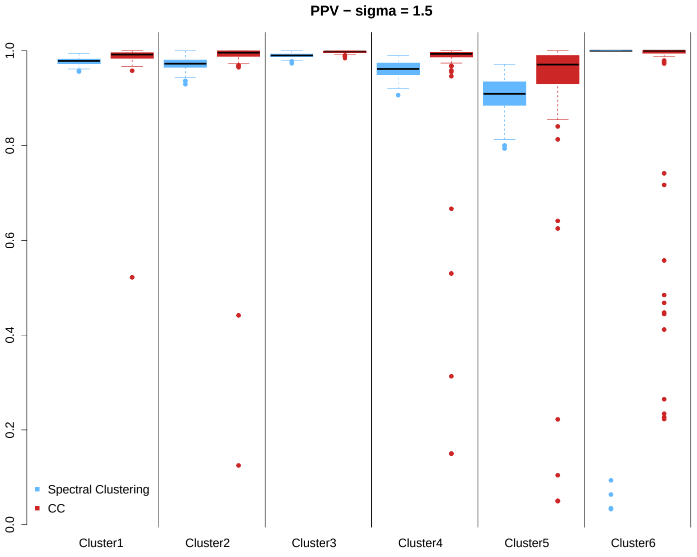

Figure S99. Boxplots of the PPV in each of the $K = 6$ simulated clusters for $S = 100$ simulations with CC and Spectral clustering when $\sigma = 1.5$. The PPV is the rate between the number of true positives and the sum of true positives and false positives. CC performed better on the first clusters, while the performance is approximately the same in the last cluster.

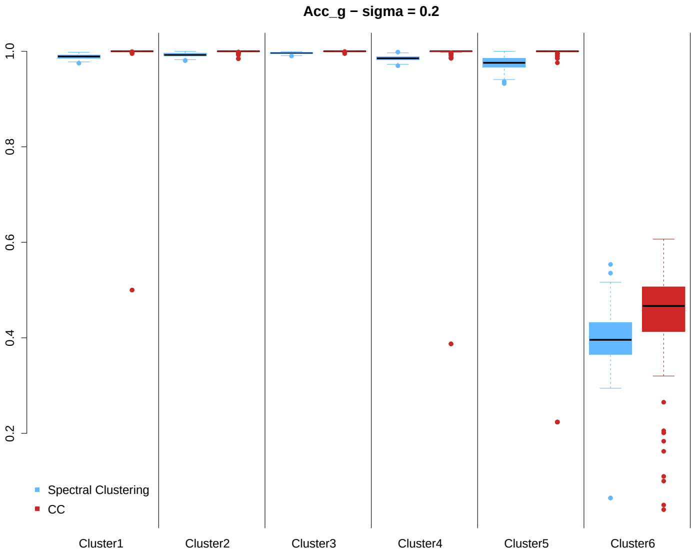

Figure S100. Boxplots of the geometric accuracy (Acc_g) in each of the $K = 6$ simulated clusters for $S = 100$ simulations with CC and Spectral clustering when $\sigma = 0.2$. The Geometric Accuracy is the geometric mean of sensitivity and PPV. CC performed always better.

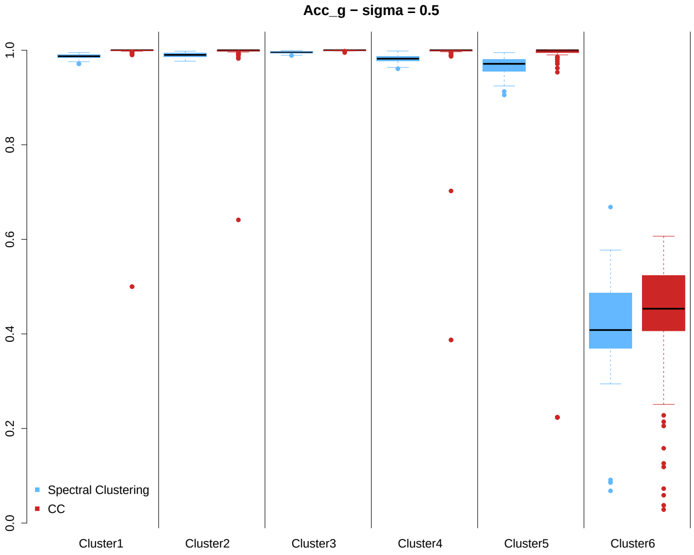

Figure S101. Boxplots of thegeometric accuracy (Acc_g) in each of the $K = 6$ simulated clusters for $S = 100$ simulations with CC and Spectral clustering when $\sigma = 0.5$.The Geometric Accuracy is the geometric mean of sensitivity and PPV. CC performed always better.

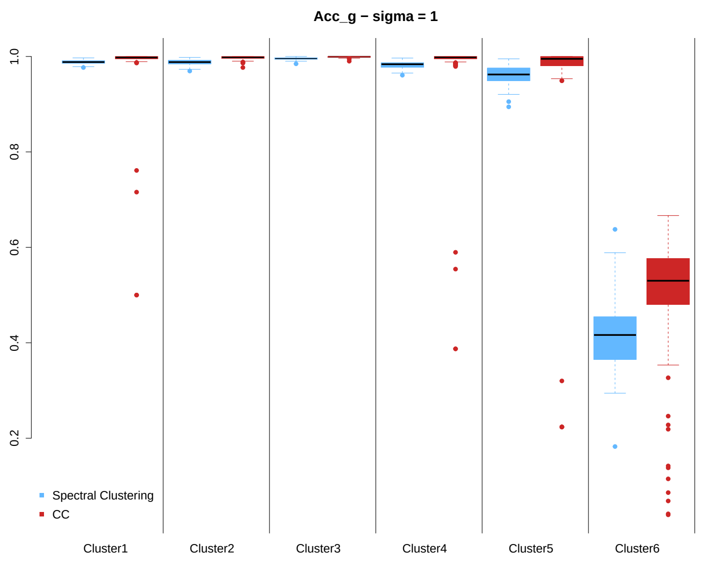

Figure S102. Boxplots of the geometric accuracy (Acc_g) in each of the $K = 6$ simulated clusters for $S = 100$ simulations with CC and Spectral clustering when $\sigma = 1$. The Geometric Accuracy is the geometric mean of sensitivity and PPV. CC performed always better.

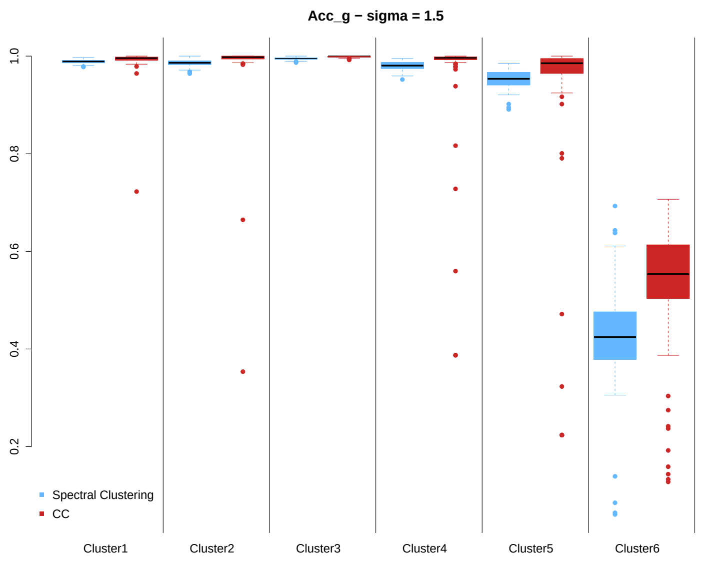

Figure S103. Boxplots of the geometric accuracy (Acc_g) in each of the $K = 6$ simulated clusters for $S = 100$ simulations with CC and Spectral clustering when $\sigma = 1.5$. The Geometric Accuracy is the geometric mean of sensitivity and PPV. CC performed always better.

Identification of the number of clusters - CC versus Complete

Figs. S104 to S107 show the ability of CC to identify the correct number of clusters versus CL combined with several methods for the identification of such number. The figures show that CC is consistent across all the 100 simulated datasets, similarly to CL with some of the methods tested.

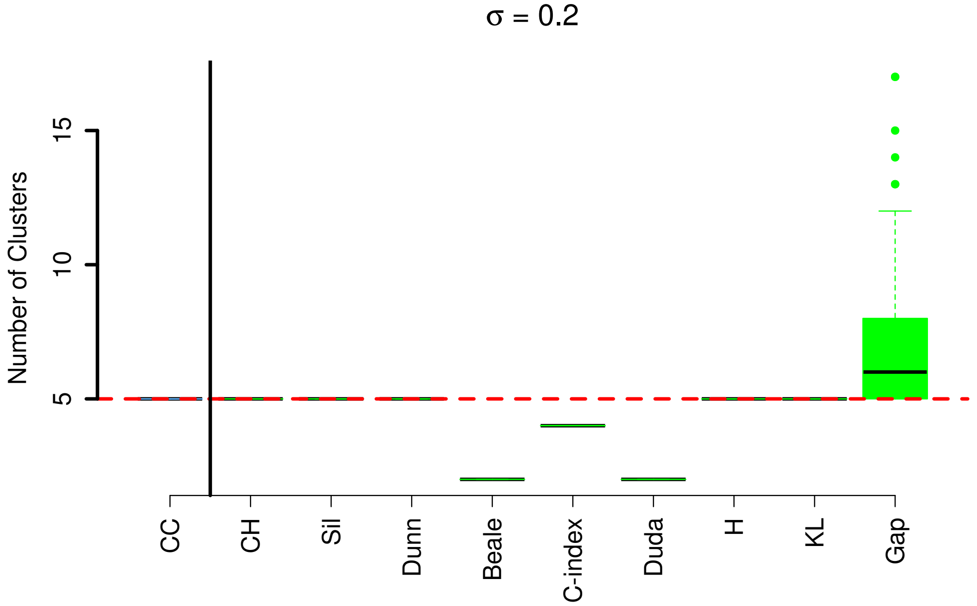

Figure S104. Boxplots reporting the number of clusters detected on simulated data with $\sigma = 0.2$ by CC and CL method using different methods, respectively: CH, average Silhouette Width, Dunn index, Beale, C-index, Duda index, H, KL, and Gap. The horizontal red line represents the actual number of clusters, $K = 5$. Here CC proved to be one of the best performing methods, always being able to detect the actual number of clusters in 100 simulations. Beale, C-index, and Duda consistently missed the actual number of clusters, while Gap has high variability with median far from reality.

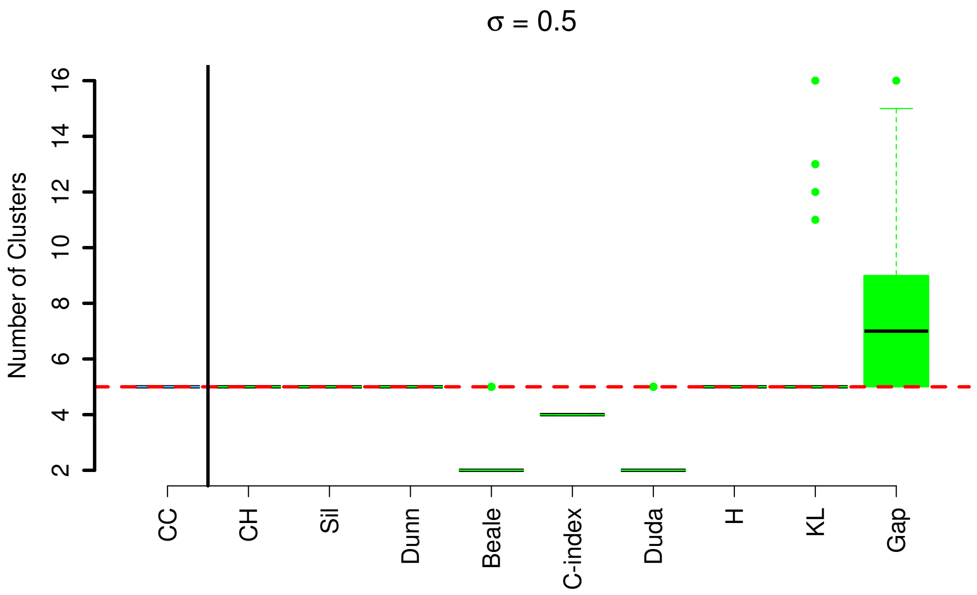

Figure S105. Boxplots reporting the number of clusters detected on simulated data with $\sigma = 0.5$ by CC and CL method using different methods, respectively: CH, average Silhouette Width, Dunn index, Beale, C-index, Duda index, H, KL, and Gap. The horizontal red line represents the actual number of clusters, $K = 5$. Here CC proved to be one of the best performing methods, always being able to detect the actual number of clusters in 100 simulations. Beale, C-index, and Duda consistently missed the actual number of clusters, while Gap has high variability with median far from reality.

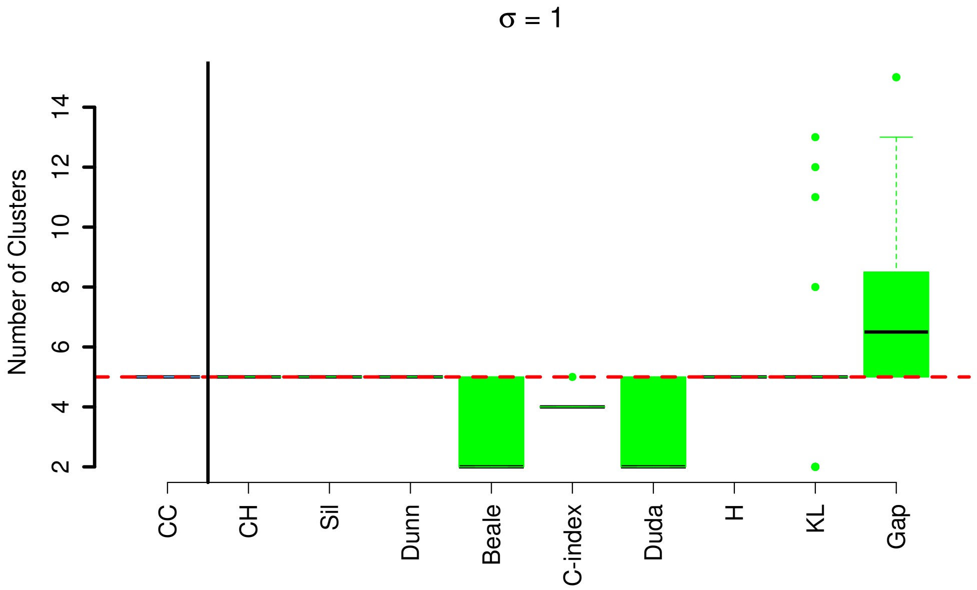

Figure S106. Boxplots reporting the number of clusters detected on simulated data with $\sigma = 1$ by CC and CL method using different methods, respectively: CH, average Silhouette Width, Dunn index, Beale, C-index, Duda index, H, KL, and Gap. The horizontal red line represents the actual number of clusters, $K = 5$. Here CC proved to be one of the best performing methods, always being able to detect the actual number of clusters in 100 simulations. C-index consistently missed the actual number of clusters, while Beale, Duda, and Gap have high variability with median far from reality. At the increase of σ even KL starts performing badly.

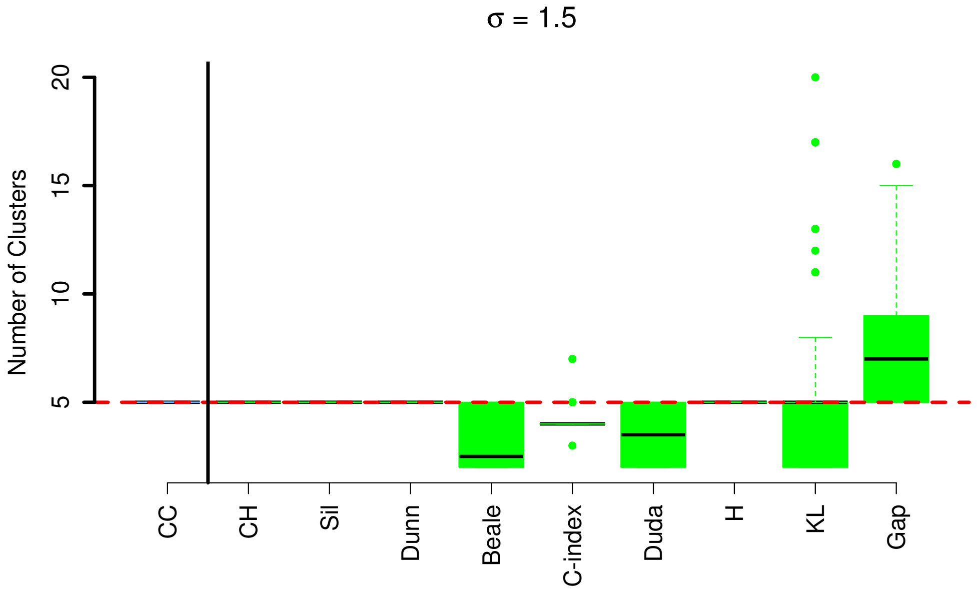

Figure S107. Boxplots reporting the number of clusters detected on simulated data with $\sigma = 1.5$ by CC and CL method using different methods, respectively: CH, average Silhouette Width, Dunn index, Beale, C-index, Duda index, H, KL, and Gap. The horizontal red line represents the actual number of clusters, $K = 5$. Here CC proved to be one of the best performing methods, always being able to detect the actual number of clusters in 100 simulations. C-index consistently missed the actual number of clusters, while Beale, Duda, and Gap have high variability with median far from reality. At the increase of σ even KL starts performing badly.

Identification of the number of clusters - CC versus Ward

Figs. S108 to S111 show the ability of CC to identify the correct number of clusters versus Ward combined with several methods for the identification of such number. The figures show that CC is consistent across all the 100 simulated datasets, similarly to Ward with some of the methods tested.

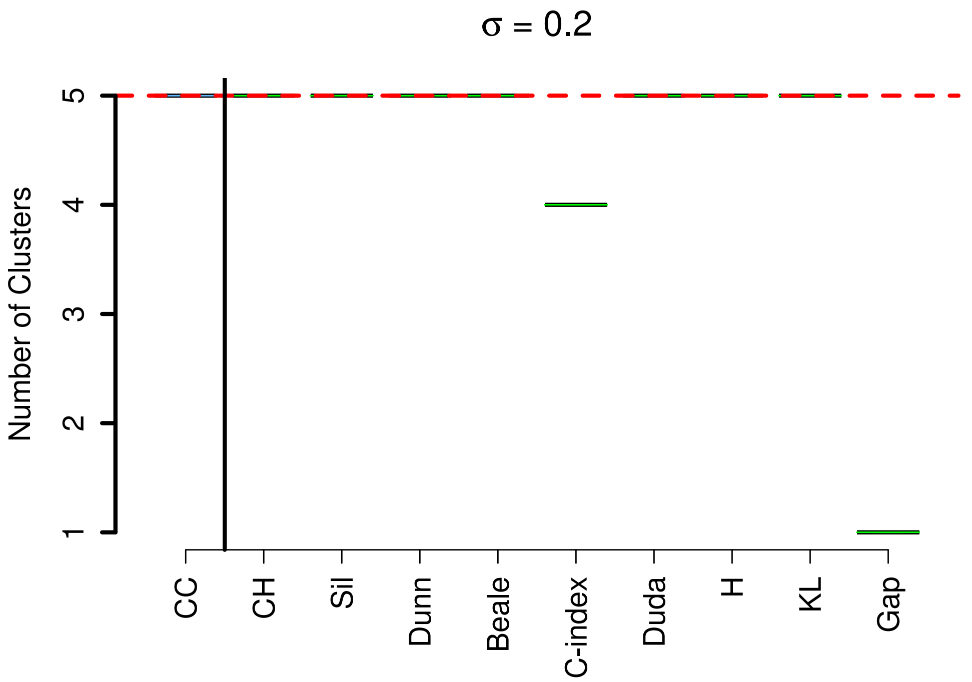

Figure S108. Boxplots reporting the number of clusters detected on simulated data with $\sigma = 0.2$ by CC and Ward's minimum variance method using different methods, respectively: CH, average Silhouette Width, Dunn index, Beale, C-index, Duda index, H, KL, and Gap. The horizontal red line represents the actual number of clusters, $K = 5$. Here CC proved to be one of the best performing methods, always being able to detect the actual number of clusters in 100 simulations. C-index and Gap consistently missed the actual number of clusters.

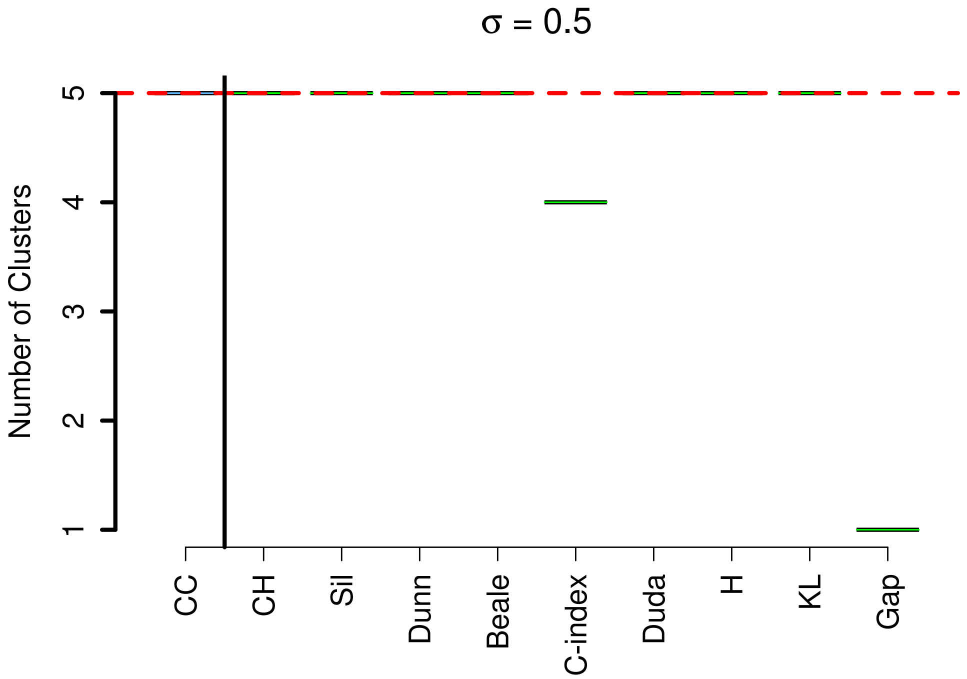

Figure S109. Boxplots reporting the number of clusters detected on simulated data with $\sigma = 0.5$ by CC and Ward's minimum variance method using different methods, respectively: CH, average Silhouette Width, Dunn index, Beale, C-index, Duda index, H, KL, and Gap. The horizontal red line represents the actual number of clusters, $K = 5$. Here CC proved to be one of the best performing methods, always being able to detect the actual number of clusters in 100 simulations. C-index and Gap consistently missed the actual number of clusters.

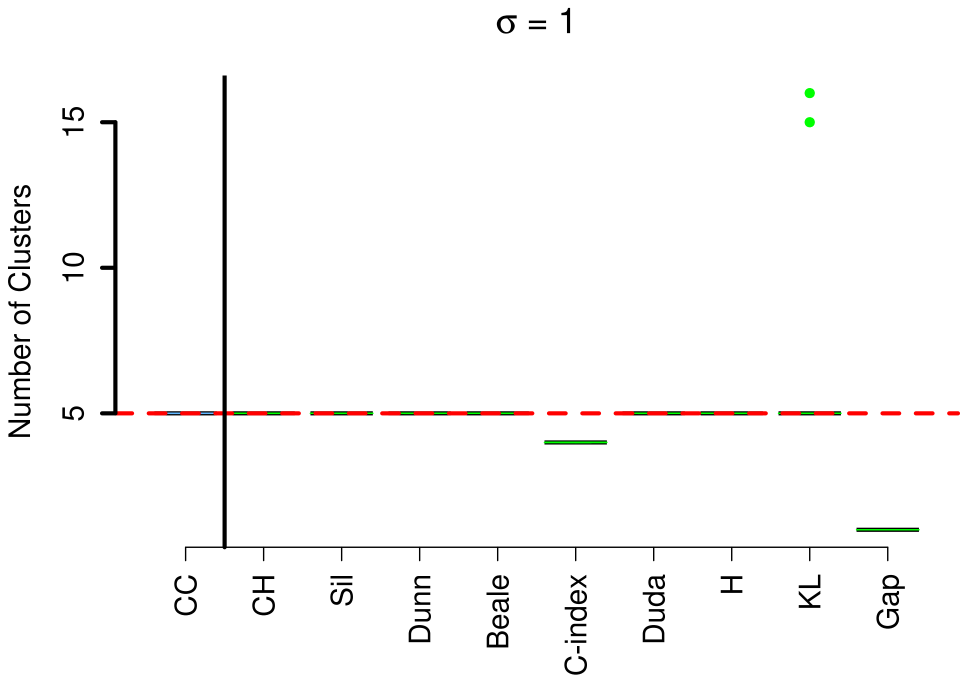

Figure S110. Boxplots reporting the number of clusters detected on simulated data with $\sigma = 1$ by CC and Ward's minimum variance method using different methods, respectively: CH, average Silhouette Width, Dunn index, Beale, C-index, Duda index, H, KL, and Gap. The horizontal red line represents the actual number of clusters, $K = 5$. Here CC proved to be one of the best performing methods, always being able to detect the actual number of clusters in 100 simulations. C-index and Gap consistently missed the actual number of clusters.

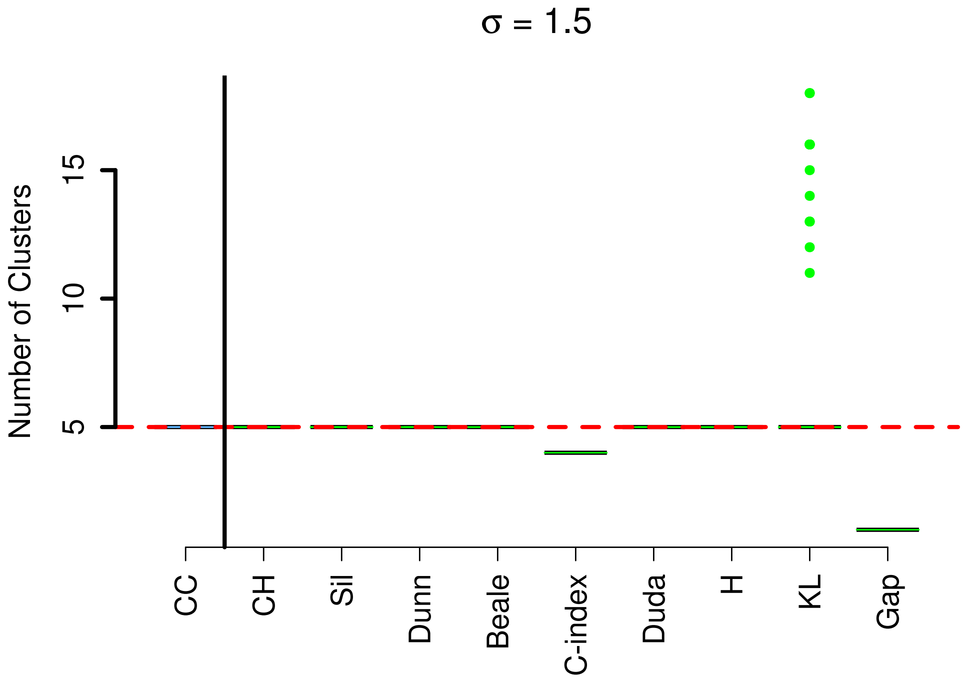

Figure S111. Boxplots reporting the number of clusters detected on simulated data with $\sigma = 1.5$ by CC and Ward's minimum variance method using different methods, respectively: CH, average Silhouette Width, Dunn index, Beale, C-index, Duda index, H, KL, and Gap. The horizontal red line represents the actual number of clusters, $K = 5$. Here CC proved to be one of the best performing methods, always being able to detect the actual number of clusters in 100 simulations. C-index and Gap consistently missed the actual number of clusters. At the increase of σ even KL starts performing badly.

Identification of the number of clusters - CC versus K-means

Figs. S112 to S115 show the ability of CC to identify the correct number of clusters versus K-means combined with several methods for the identification of such number. The figures show that CC is consistent across all the 100 simulated datasets, similarly to K-means with Jump method.

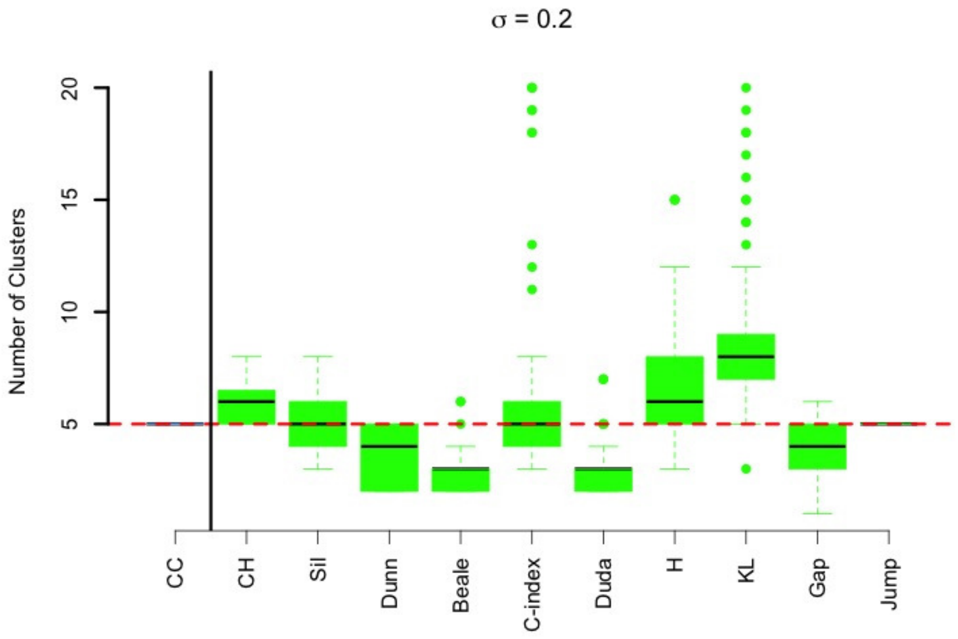

Figure S112. Boxplots reporting the number of clusters detected on simulated data with $\sigma = 0.2$ by CC and K-means using different methods, respectively: CH, average Silhouette Width, Dunn index, Beale, C-index, Duda index, H, KL, Gap, and Jump. The horizontal red line represents the actual number of clusters, $K = 5$. Here CC and Jump methods proved to be the best performing methods, showing the smaller variability of results in 100 simulations.

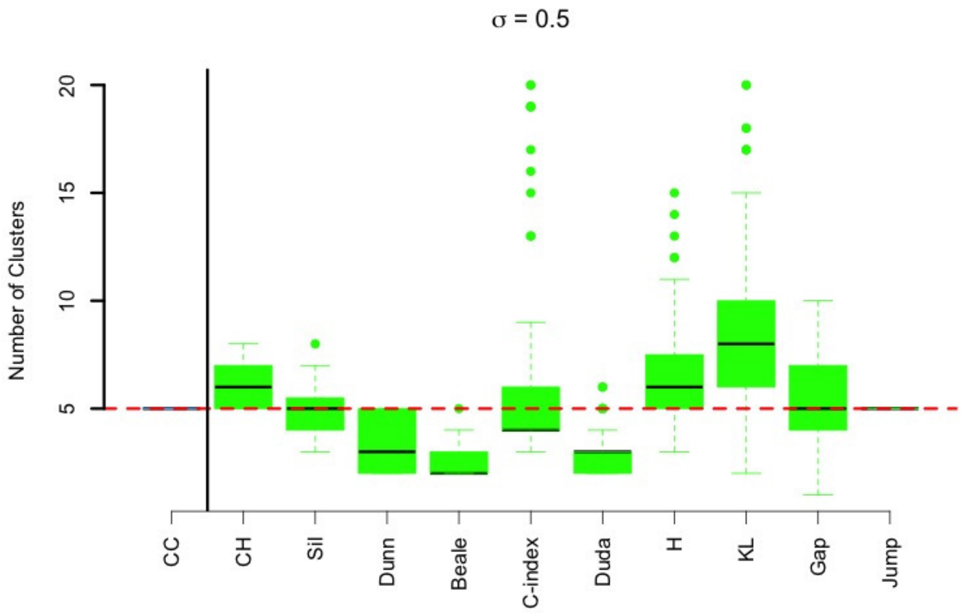

Figure S113. Boxplots reporting the number of clusters detected on simulated data with $\sigma = 0.5$ by CC and K-means using different methods, respectively: CH, average Silhouette Width, Dunn index, Beale, C-index, Duda index, H, KL, Gap, and Jump. The horizontal red line represents the actual number of clusters, $K = 5$. Here CC and Jump methods proved to be the best performing methods, , always being able to detect the actual number of clusters in 100 simulations.

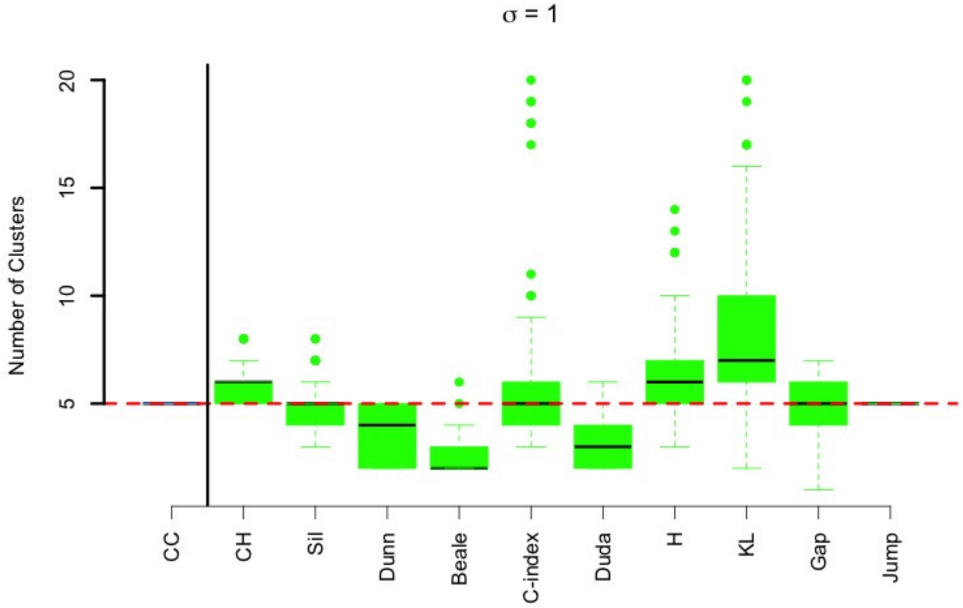

Figure S114. Boxplots reporting the number of clusters detected on simulated data with $\sigma = 1$ by CC and K-means using different methods, respectively: CH, average Silhouette Width, Dunn index, Beale, C-index, Duda index, H, KL, Gap, and Jump. The horizontal red line represents the actual number of clusters, $K = 5$. Here CC and Jump methods proved to be the best performing methods, , always being able to detect the actual number of clusters in 100 simulations.

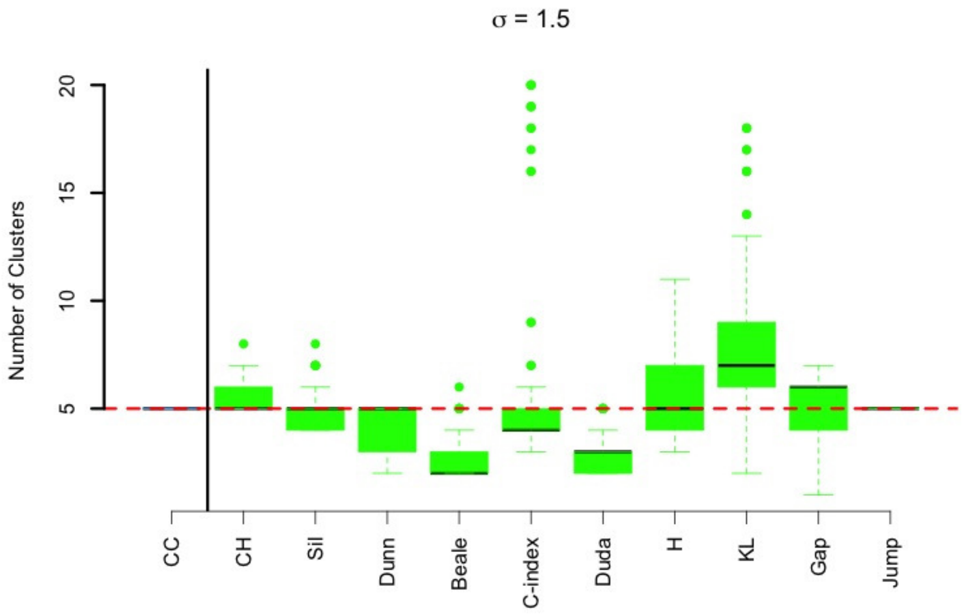

Figure S115. Boxplots reporting the number of clusters detected on simulated data with $\sigma = 1.5$ by CC and K-means using different methods, respectively: CH, average Silhouette Width, Dunn index, Beale, C-index, Duda index, H, KL, Gap, and Jump. The horizontal red line represents the actual number of clusters, $K = 5$. Here CC and Jump methods proved to be the best performing methods, , always being able to detect the actual number of clusters in 100 simulations.

Stability of CC

We checked the stability of CC with regard to the input parameters. We performed CC with 10 different pairs of values (shown in Fig. S116) for the boundaries of I^W, while setting a high $n_{c_{max}} = 99$ on the simulated data after the removal of outliers. Figs. S117 to S120 prove the robustness of the method, which is always able in 100 simulations to identify the correct number of clusters ($K = 5$).

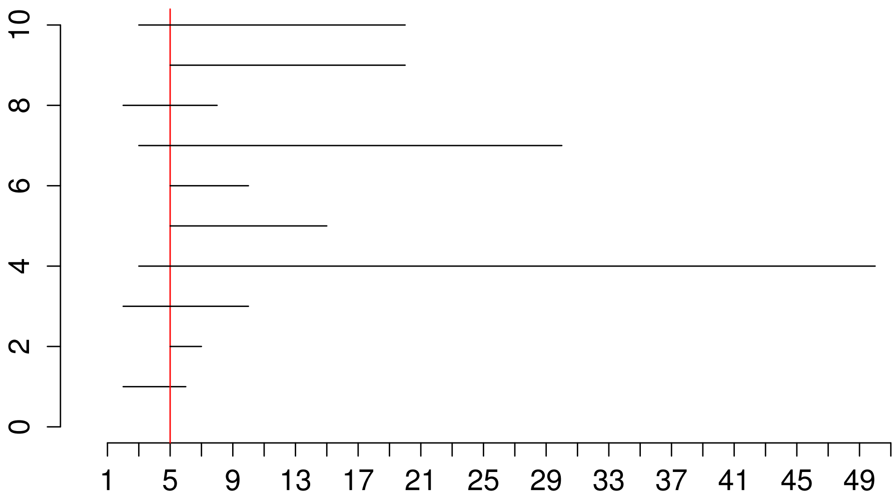

Figure S116. Representation of the boundaries of the ten different intervals I^W used for choosing the number of clusters in the Ward's minimum variance method inside the CC algorithm. The red line represents the correct number of clusters, $k = 5$, in simulated data after the removal of outliers. The length of the line represents the width of the boundary.

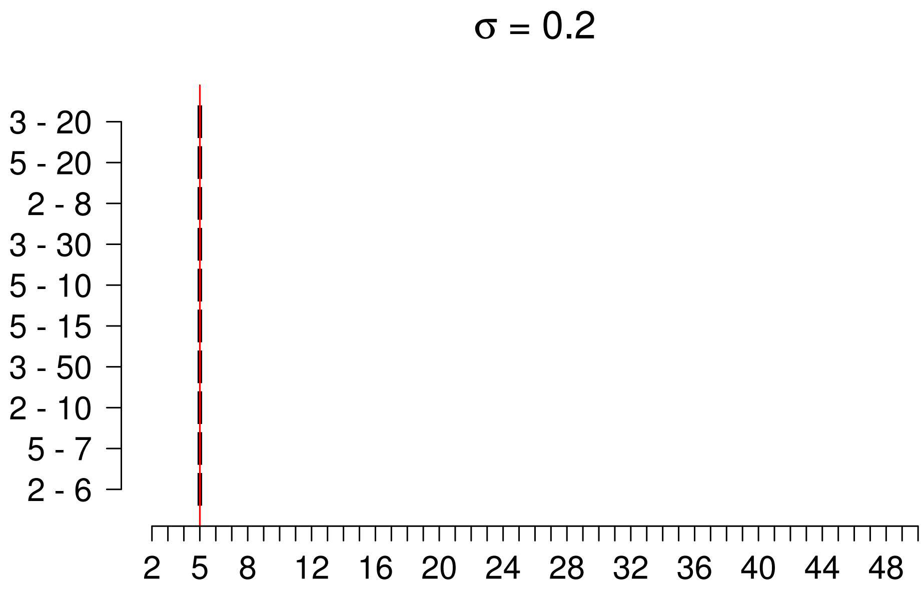

Figure S117. Boxplots of the number of clusters found by the CC algorithm with ten different intervals I^W and with $\sigma = 0.2$. The vertical line represents the correct number of clusters, $k = 5$. Labels on the y-axis represent the lower and upper boundaries of each interval. The maximum number of clusters for the CL is always set equal 99. In this case CC was always able to detect the actual number of clusters.

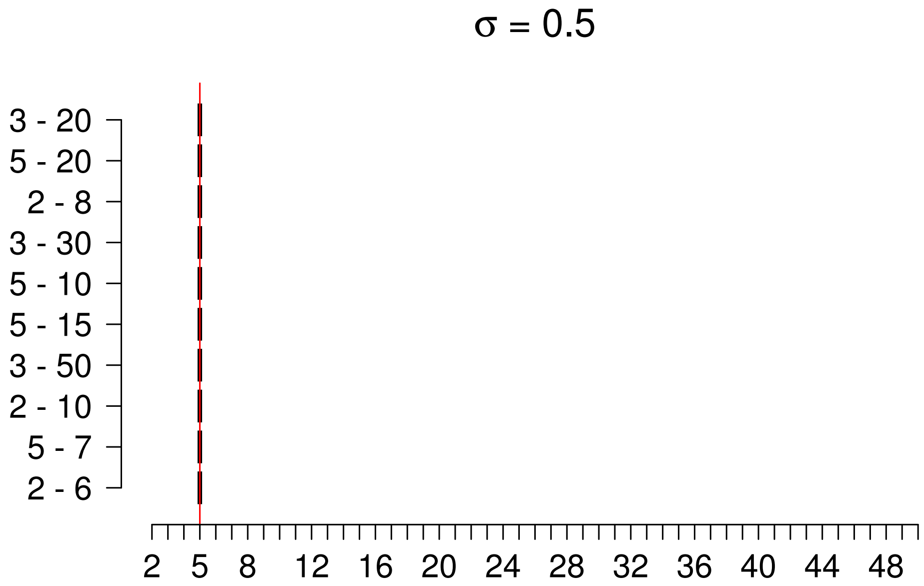

Figure S118. Boxplots of the number of clusters found by the CC algorithm with ten different intervals I^W and with $\sigma = 0.5$. The vertical line represents the correct number of clusters, $k = 5$. Labels on the y-axis represent the lower and upper boundaries of each interval. The maximum number of clusters for the CL is always set equal 99. In this case CC was always able to detect the actual number of clusters.

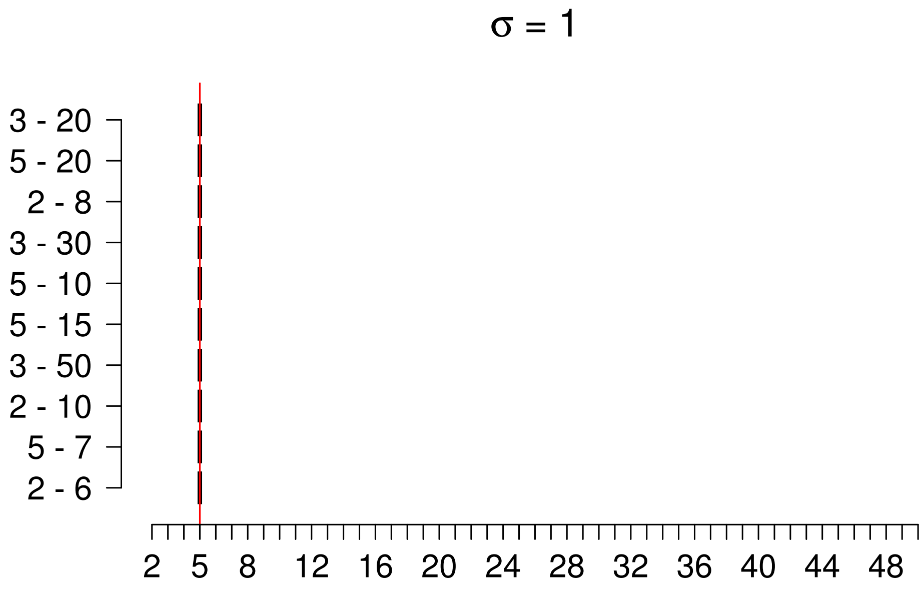

Figure S119. Boxplots of the number of clusters found by the CC algorithm with ten different intervals I^W and with $\sigma = 1$. The vertical line represents the correct number of clusters, $k = 5$. Labels on the y-axis represent the lower and upper boundaries of each interval. The maximum number of clusters for the CL is always set equal 99. In this case CC was always able to detect the actual number of clusters.

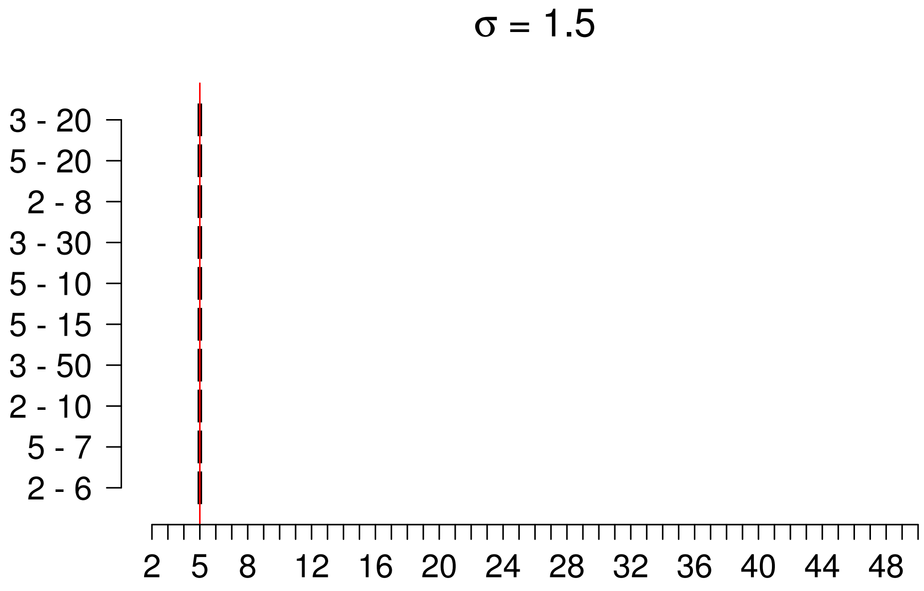

Figure S120. Boxplots of the number of clusters found by the CC algorithm with ten different intervals I^W and with $\sigma = 1.5$. The vertical line represents the correct number of clusters, $k = 5$. Labels on the y-axis represent the lower and upper boundaries of each interval. The maximum number of clusters for the CL is always set equal 99. In this case CC was always able to detect the actual number of clusters.

Brain tumors dataset

Comb. #	$x$dim	$y$dim	Tot.
1	1	2	2
2	1	3	3
3	1	4	4
4	1	5	5
5	1	6	6
6	1	7	7
7	1	8	8
8	1	9	9
9	1	10	10
10	1	11	11
11	1	12	12
12	1	13	13
13	1	14	14
14	1	15	15
15	1	16	16
16	1	17	17
17	1	18	18
18	1	19	19
19	1	20	20
20	2	1	2
21	2	2	4
22	2	3	6
23	2	4	8
24	2	5	10
25	2	6	12
26	2	7	14
27	2	8	16
28	2	9	18
29	2	10	20
30	3	1	3
31	3	2	6
32	3	3	9
33	3	4	12
34	3	5	15
35	3	6	18

Comb. #	$x$dim	$y$dim	Tot.
36	3	7	21
37	4	1	4
38	4	2	8
39	4	3	12
40	4	4	16
41	4	5	20
42	5	1	5
43	5	2	10
44	5	3	15
45	5	4	20
46	6	1	6
47	6	2	12
48	6	3	18
49	6	4	24
50	7	1	7
51	7	2	14
52	7	3	21
53	8	1	8
54	8	2	16
55	8	3	24
56	9	1	9
57	9	2	18
58	9	3	27
59	10	1	1
60	10	2	20
61	11	1	11
62	12	1	12
63	13	1	13
64	14	1	14
65	15	1	15
66	16	1	16
67	17	1	17
68	18	1	18
69	19	1	19
70	20	1	20

Table S6. 70 combinations of x and y dimensions for the SOM's grid in order to decide which one to use on the brain tumors dataset. The last column of both tables reports the total number of resulting available cells.

Parameters for SOM

We applied SOM with a rectangular topology, Euclidean distance, number of training iterations $rlen = 100$, learning rate (amount of change) α decreasing linearly from 0.05 to 0.01, the radius of the neighborhood starts with a value that covers 2/3 of all unit-to-unit distances, and initial values for each node is chosen randomly without replacement from the data.

Parameters for DBSCAN

The idea is that points in a cluster are roughly at same distance from their n^{th} nearest neighbor, while the distance from noise points is higher. The suggested n for

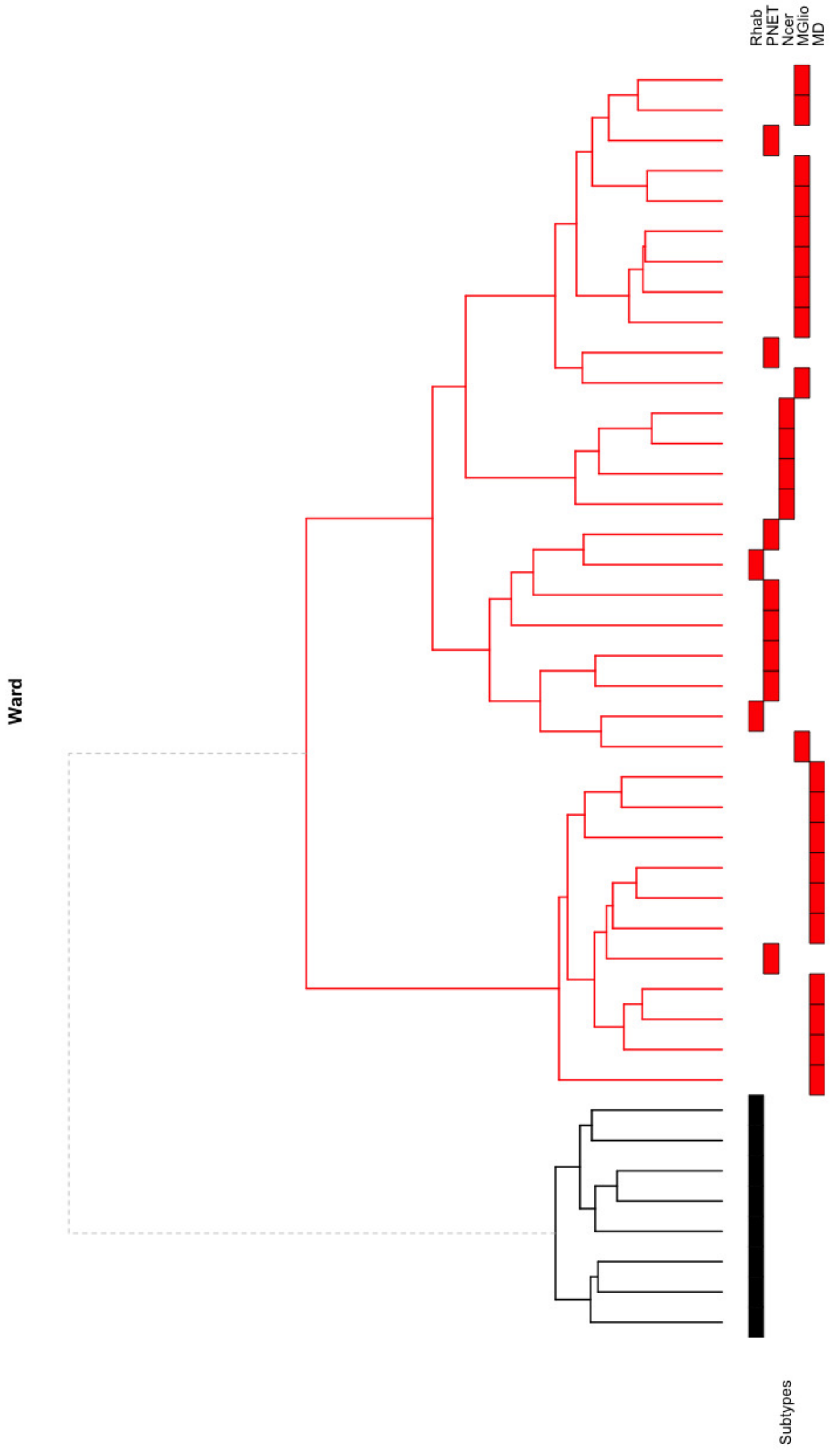

Figure S121. Dendrogram resulting from clustering 42 brain tumors samples with Ward. Leafs are colored depending on the results of the clustering. Boxes in the bottom represent the real subtypes.

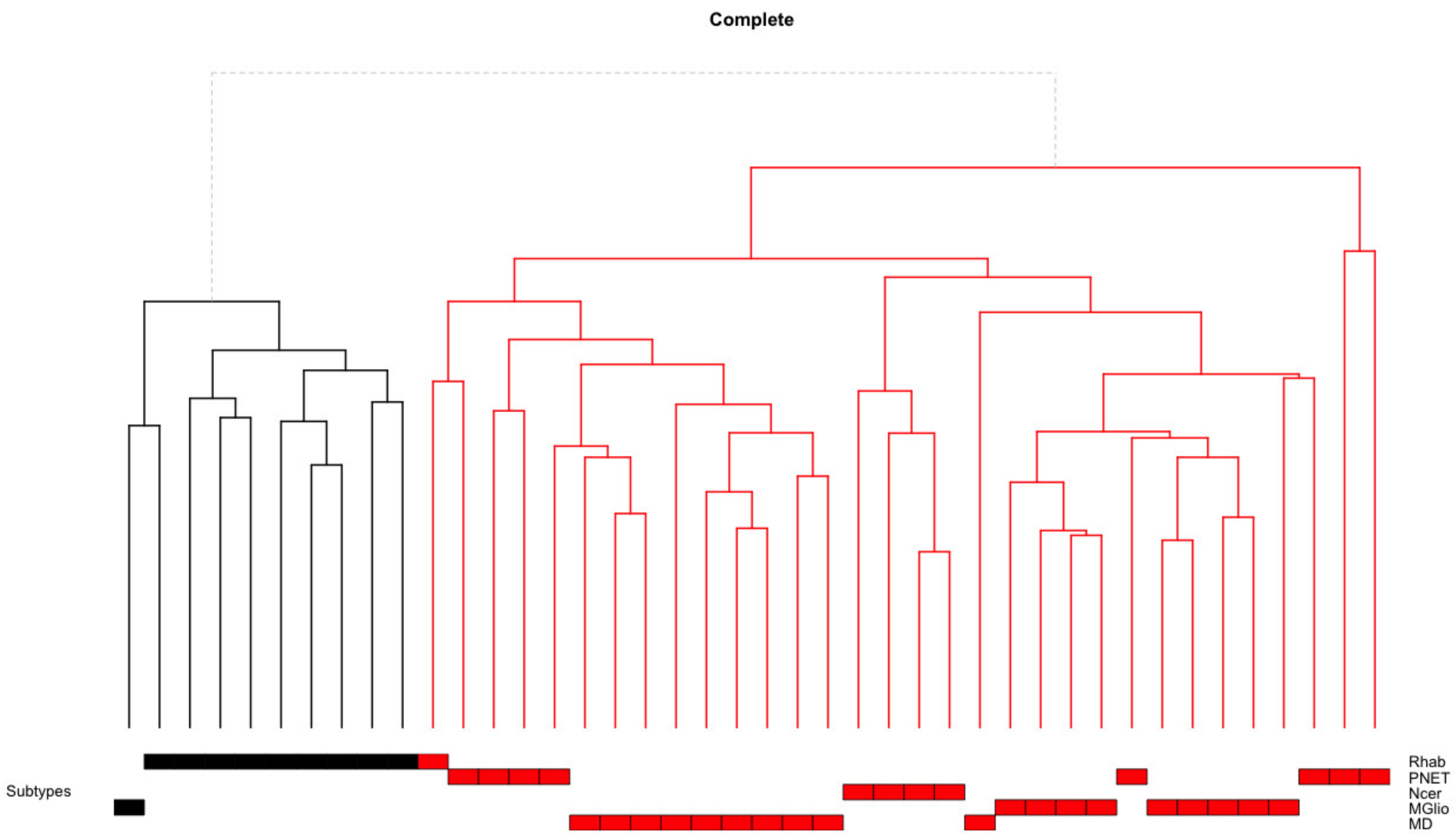

Figure S122. Dendrogram resulting from clustering 42 brain tumors samples with CL. Leafs are colored depending on the results of the clustering. Boxes in the bottom represent the real subtypes.

two-dimensional data is 4. Therefore, plotting the sorted distances, sorted in descending order, of each point from its nth nearest neighbor gives hints concerning the proximity of the elements in the data. A threshold point p should be chosen to be the first one in the first “valley” of the sorted distances: all the points on the left of the threshold are considered to be noise, while all other points are assigned to some cluster. The parameters are then set such as $\epsilon = dist(p)$ and $MinPts = n$.

DBSCAN results

On the lung cancer dataset we tried different types of n-th nearest neighbors ($n \in [4, 7]$), obtaining $\epsilon = 209.969$ for $MinPts = 4$; $\epsilon = 213.4471$ for $MinPts = 5$; $\epsilon = 215.1246$ for $MinPts = 6$; $\epsilon = 215.296$ for $MinPts = 7$.

	MD	MGlio	Ncer	PNET	Rhab	
Clu1	10	10	4	8	2	34
Clu2	0	0	0	0	**8**	8
	10	10	4	8	10	42

Table S7. Contingency table reporting both the real classification in subtypes and the clusters identified by the Ward’s minimum variance method on the brain tumors dataset. Values in bold are those resulted statistically significant after the over-representation analysis.

	MD	MGlio	Ncer	PNET	Rhab	
Clu1	10	9	2	1	**10**	32
Clu2	0	1	2	7	0	10
	10	10	4	8	10	42

Table S8. Contingency table reporting both the real classification in subtypes and the clusters identified by the CL method on the brain tumors dataset. Values in bold are those resulted statistically significant after the over-representation analysis.

	MD	MGlio	Ncer	PNET	Rhab	
Clu1	10	9	1	1	9	30
Clu2	0	0	1	0	1	2
Clu3	0	1	2	**7**	0	10
	10	10	4	8	10	42

Table S9. Contingency table reporting both the real classification in subtypes and the clusters identified by the K-means method on the brain tumors dataset. Values in bold are those resulted statistically significant after the over-representation analysis.

	MD	MGlio	Ncer	PNET	Rhab	
Clu1	0	0	0	1	0	1
Clu2	10	10	4	7	10	41
	10	10	4	8	10	42

Table S10. Contingency table reporting both the real classification in subtypes and the clusters identified by the SOM method on the brain tumors dataset. No subtypes are over-represented by any cluster.

	MD	MGlio	Ncer	PNET	Rhab	
Outliers	1	0	0	0	0	1
Clu1	0	**9**	0	2	0	11
Clu2	**9**	0	0	1	0	10
Clu3	0	0	0	0	**8**	8
Clu4	0	0	**4**	0	0	4
Clu5	0	1	0	0	1	2
Clu6	0	0	0	1	1	2
Clu7	0	0	0	2	0	2
Clu8	0	0	0	1	0	1
Clu9	0	0	0	1	0	1
	10	10	4	8	10	42

Table S11. Contingency table reporting both the real classification in subtypes and the clusters identified by CC on the brain tumors dataset. Values in bold are those resulted statistically significant after the over-representation analysis. Although CC identified more than five clusters, four of them almost perfectly represented MD, MGlio, Ncer, and Rhab subtypes. The PNET subtype was represented by six of CC clusters, but it is well known for having an high heterogeneity.

	Results	
Method	# Clusters	ARI
Ward	2	0.14
CL	2	0.18
K-means	3	0.19
SOM	2	0.003
CC	9 + 1	**0.64**

Table S12. Summarization of results on brain tumors dataset with Euclidean distance. The table reports every method applied with the resulting number of clusters and adjusted Rand Index (ARI) (the higher the better). The actual number of subtypes in the dataset was five. CC identified nine clusters plus one cluster containing one outlier, leading to the highest ARI. None of the other methods was able to identify the correct number of clusters. Although CC identified more than five clusters, four of them almost perfectly represented MD, MGlio, Ncer, and Rhab subtypes. The PNET subtype was represented by six of CC clusters, but it is well known for having an high heterogeneity.

Breast cancer dataset

	Results	
Method	# Clusters	ARI
Ward	11	0.09
CL	11	0.10
K-means	11	0.04
SOM	18	0.07
CC	2 + 1	**0.63**

Table S13. Summarization of results on breast cancer dataset with Euclidean distance. The table reports every method applied with the resulting number of clusters and adjusted Rand Index (ARI) (the higher the better). The actual number of subtypes in the dataset was two. CC identified two clusters plus one cluster containing two outliers, leading to the highest ARI.

	Luminal	TN	
Clu1	6	1	7
Clu2	2	4	6
Clu3	1	0	1
Clu4	2	0	2
Clu5	2	0	2
Clu6	0	2	2
Clu7	0	2	2
Clu8	0	2	2
Clu9	0	2	2
Clu10	0	1	1
Clu11	3	0	3
	16	14	30

Table S14. Contingency table reporting both the real classification in subtypes and the clusters identified by the Ward's minimum variance method on the breast cancer dataset. No subtypes are over-represented by any cluster.

	Luminal	TN	
Clu1	3	5	8
Clu2	4	2	6
Clu3	1	0	1
Clu4	2	0	2
Clu5	1	1	2
Clu6	2	0	2
Clu7	0	2	2
Clu8	0	2	2
Clu9	2	0	2
Clu10	1	0	1
Clu11	0	2	2
	16	14	30

Table S15. Contingency table reporting both the real classification in subtypes and the clusters identified by the Complete-linkage method on the breast cancer dataset. No subtypes are over-represented by any cluster.

	Luminal	TN	
1	2	0	2
2	1	0	1
4	0	2	2
6	0	1	1
7	1	0	1
8	0	2	2
9	2	1	3
10	2	0	2
11	1	0	1
12	0	1	1
13	0	3	3
14	0	2	2
15	0	2	2
16	1	0	1
17	2	0	2
18	4	0	4
	16	14	30

Table S16. Contingency table reporting both the real classification in subtypes and the clusters identified by SOM on the breast cancer dataset. No subtypes are over-represented by any cluster.

	Luminal	TN	
Clu1	1	0	1
Clu2	3	1	4
Clu3	0	2	2
Clu4	0	2	2
Clu5	1	1	2
Clu6	1	0	1
Clu7	4	5	9
Clu8	2	0	2
Clu9	2	0	2
Clu10	2	0	2
Clu11	0	3	3
	16	14	30

Table S17. Contingency table reporting both the real classification in subtypes and the clusters identified by K-means on the breast cancer dataset. No subtypes are over-represented by any cluster.

	Luminal	TN	
Outliers	1	1	2
Clu1	2	**13**	15
Clu2	**13**	0	13
	16	14	30

Table S18. Contingency table reporting both the real classification in subtypes and the clusters identified by Cross-clustering on the breast cancer dataset. Values in bold are those resulted statistically significant after the over-representation analysis.

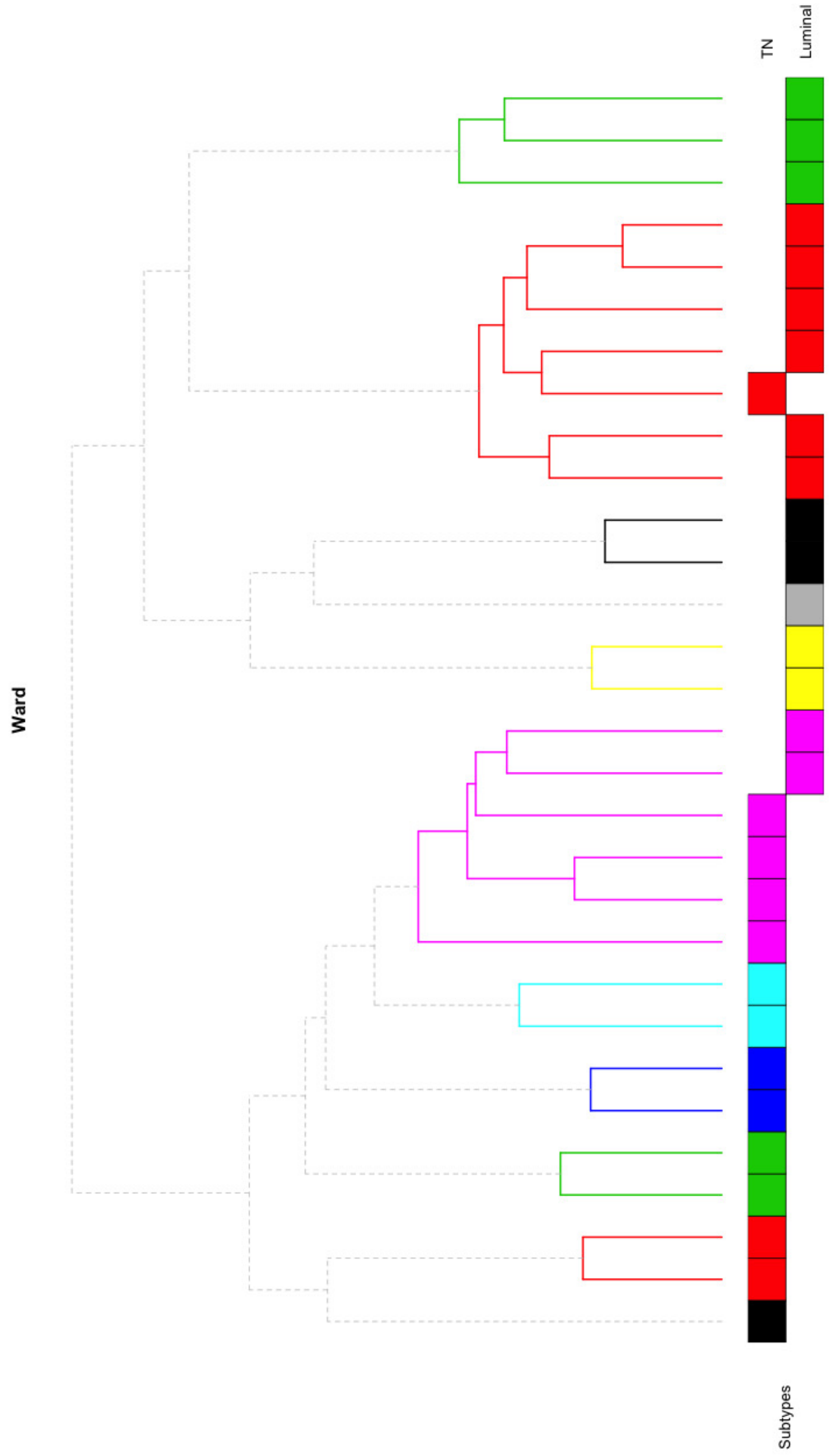

Figure S123. Dendrogram resulting from clustering 30 breast cancer tumor samples with Ward on Euclidean distance. Leafs are colored depending on the results of the clustering. Boxes in the bottom represent the real subtypes.

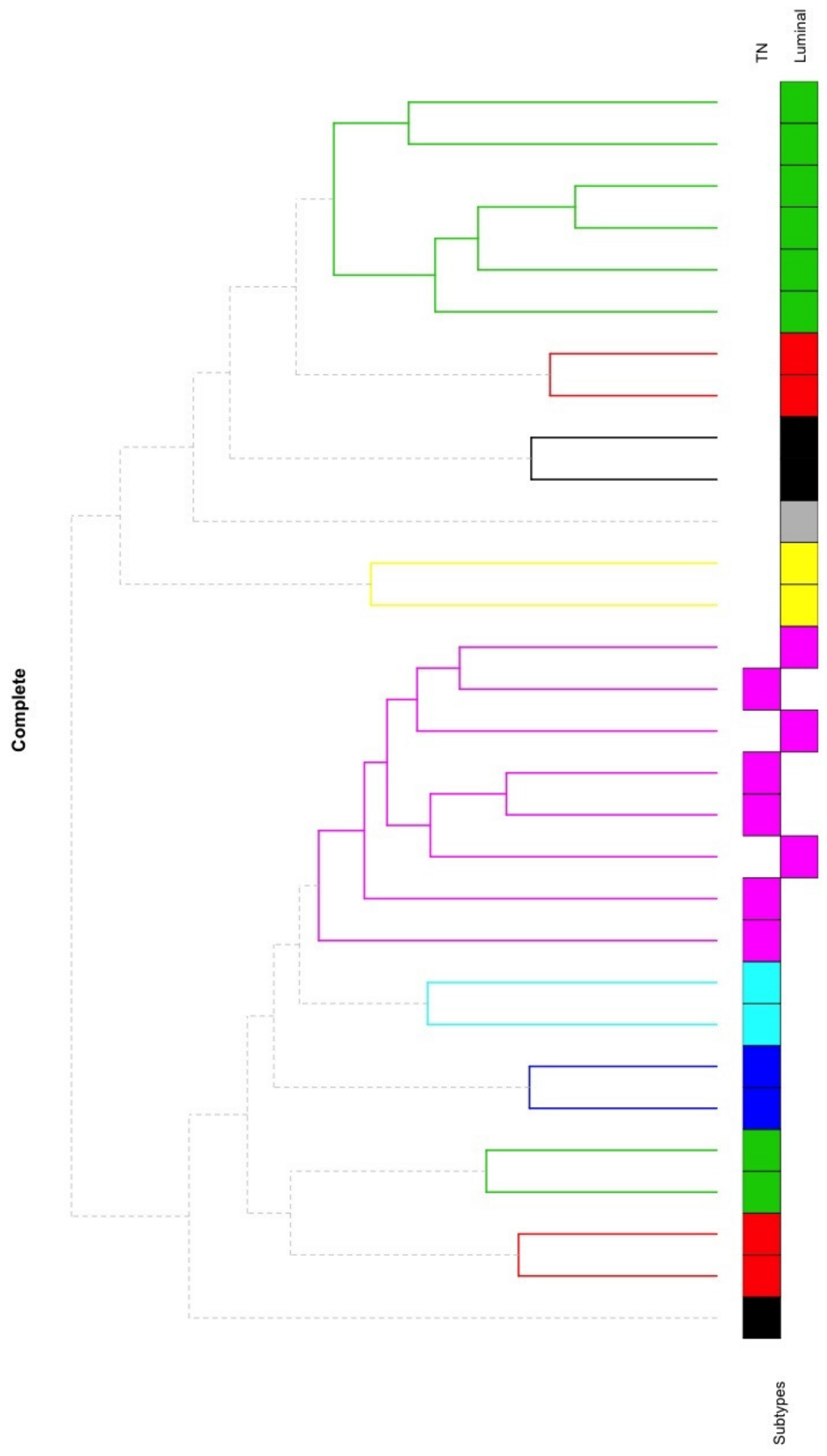

Figure S124. Dendrogram resulting from clustering 30 breast cancer tumor samples with CL on Euclidean distance. Leafs are colored depending on the results of the clustering. Boxes in the bottom represent the real subtypes.

Olive oil dataset

	Results	
Method	# Clusters	ARI
Ward	2	0.29
CL	2	0.31
K-means	2	0.29
SOM	2	0.30
CC	3 + 1	**0.60**

Table S19. Summarization of results on the olive oil dataset with Euclidean distance. The table reports every method applied with the resulting number of clusters and adjusted Rand Index (ARI) (the higher the better). The actual number of clusters in the dataset was nine. CC identified three clusters plus one cluster containing 114 outliers, leading to the highest ARI.

	Clu1	Clu2	
Apulia north	**24**	1	25
Calabria	5	**51**	56
Apulia south	0	**206**	206
Sicily	15	21	36
Sardinia inland	0	**65**	65
Sardinia coast	0	**33**	33
Liguria east	**49**	1	50
Liguria west	**48**	2	50
Umbria	**51**	0	51
	192	380	572

Table S20. Contingency table reporting both the real classification in subtypes and the clusters identified by Ward's minimum variance method on the olive oil dataset. Values in bold are those resulted statistically significant after the over-representation analysis.

	Clu1	Clu2	
Apulia north	**25**	0	25
Calabria	**47**	9	56
Apulia south	6	**200**	206
Sicily	25	11	36
Sardinia inland	**63**	2	65
Sardinia coast	0	**33**	33
Liguria east	**50**	0	50
Liguria west	**50**	0	50
Umbria	**51**	0	51
	317	255	572

Table S21. Contingency table reporting both the real classification in subtypes and the clusters identified by Complete-linkage method on the olive oil dataset. Values in bold are those resulted statistically significant after the over-representation analysis.

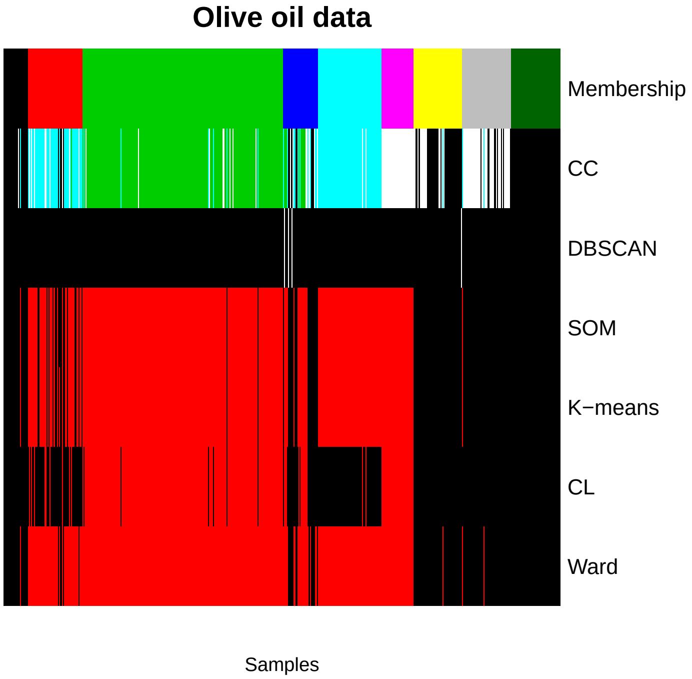

Figure S125. Graphical representation of the true membership (first row) of the 572 specimens of oliveoil in olive oil data, compared with the memberships resulting from CC, DBSCAN, SOM, K-means, CL, and Ward. The nine natural clusters in the order of the first row of the image are: Apulia north, Calabria, Apulia south, Sicily, Sardinia inland, Sardinia coast, Liguria east, Liguria west, Umbria. The colors represent the index of the cluster fiven by each method. The white color represents outliers, only detected by CC and DBSCAN. Classical approaches performed poorly, obtaining ARI values ranging around 0.30. In terms of number of clusters, the ASW criterion always identified 2 clusters. In contrast, CC obtained an ARI of 0.60, identifying three clusters and one sample as an outlier.

	Clu1	Clu2	
Apulia north	1	**24**	25
Calabria	39	17	56
Apulia south	**204**	2	206
Sicily	15	21	36
Sardinia inland	**65**	0	65
Sardinia coast	**33**	0	33
Liguria east	0	**50**	50
Liguria west	1	**49**	50
Umbria	**51**	0	51
	358	214	572

Table S22. Contingency table reporting both the real classification in subtypes and the clusters identified by K-means method on the olive oil dataset. Values in bold are those resulted statistically significant after the over-representation analysis.

	Clu1	Clu2	
Apulia north	**24**	1	25
Calabria	18	38	56
Apulia south	2	**204**	206
Sicily	21	15	36
Sardinia inland	0	**65**	65
Sardinia coast	0	**33**	33
Liguria east	**50**	0	50
Liguria west	**49**	1	50
Umbria	**51**	0	51
	215	357	572

Table S23. Contingency table reporting both the real classification in subtypes and the clusters identified by SOM method on the olive oil dataset. Values in bold are those resulted statistically significant after the over-representation analysis.

	Outliers	Clu1	Clu2	Clu3	
Apulia north	1	0	**23**	1	25
Calabria	9	1	4	**42**	56
Apulia south	8	**192**	0	6	206
Sicily	8	9	9	10	36
Sardinia inland	2	0	0	**63**	65
Sardinia coast	**33**	0	0	0	33
Liguria east	14	0	**35**	1	50
Liguria west	**39**	0	9	2	50
Umbria	0	0	**51**	0	51
	114	202	131	125	572

Table S24. Contingency table reporting both the real classification in subtypes and the clusters identified by Cross-clustering method on the olive oil dataset. Values in bold are those resulted statistically significant after the over-representation analysis.

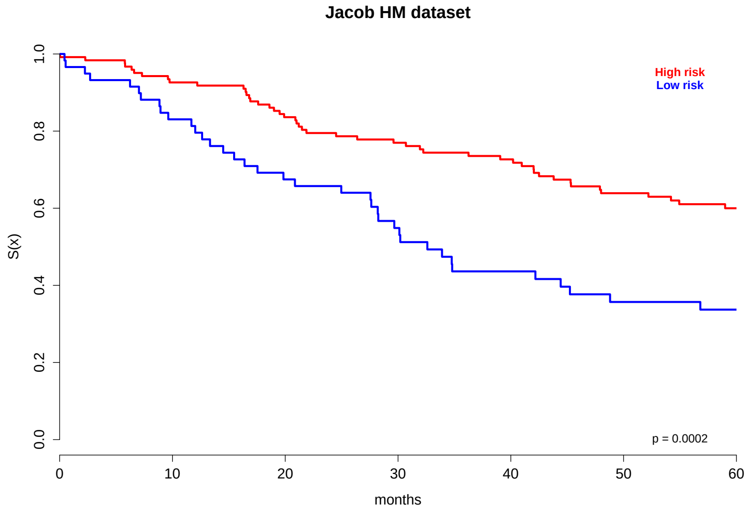

Figure S126. Kaplan-Meier survival curves of the 2 clusters obtained with CC on the HM subgroup of the Jacob dataset. The log-rank test p-value for the difference among the survival curves resulted equal 0.0002.

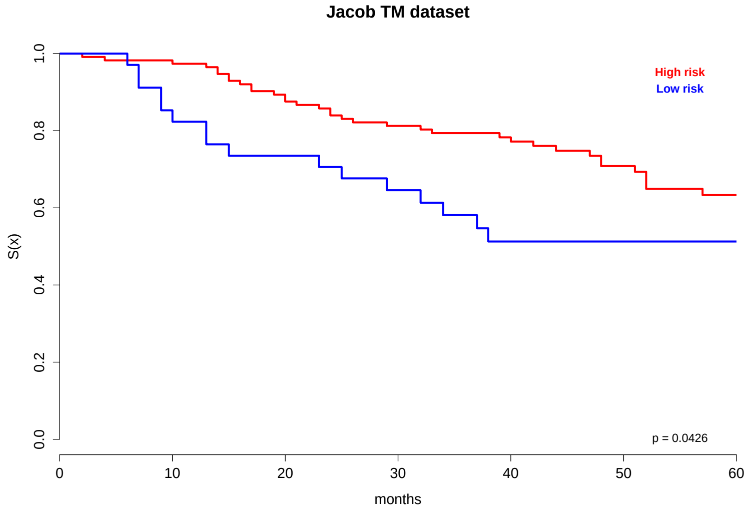

Figure S127. Kaplan-Meier survival curves of the 2 clusters obtained with CC on the TM subgroup of the Jacob dataset. The log-rank test p-value for the difference among the survival curves resulted equal 0.0426.

Running times

	σ			
	**0.2**	**0.5**	**1.0**	**1.5**
K-means	5.04s	5.50s	6.23s	6.41s
Complete	3.30m	3.41m	3.64m	3.65m
Ward	3.22m	7.99m	5.05m	3.86m
CC	6.85m	6.85m	6.99m	7.15m
autoSOME	25.67m	30.83m	34.63m	38.6
Affinity Propagation	3.66h	3.36h	3.43h	3.90h
PAM	9.58h	11.30h	18.25h	21.94h
Spectral Clustering	73.08h	71.43h	64.95h	70.82h

Table S25. Running times of different methods in clustering the same 100 simulated data sets, sorted by increasing average time. Here *s* stands for "seconds", *m* stands for "minutes", and *h* for "hours". Computer information: Windows Server 2012 R2 Standard (64bit). QUAD-CORE AMD OPTERON(tm) Processor 8356 (4 2.30GHz quad-cores). 128GB RAM. Spectral clustering has been parallelized on 16 cores.

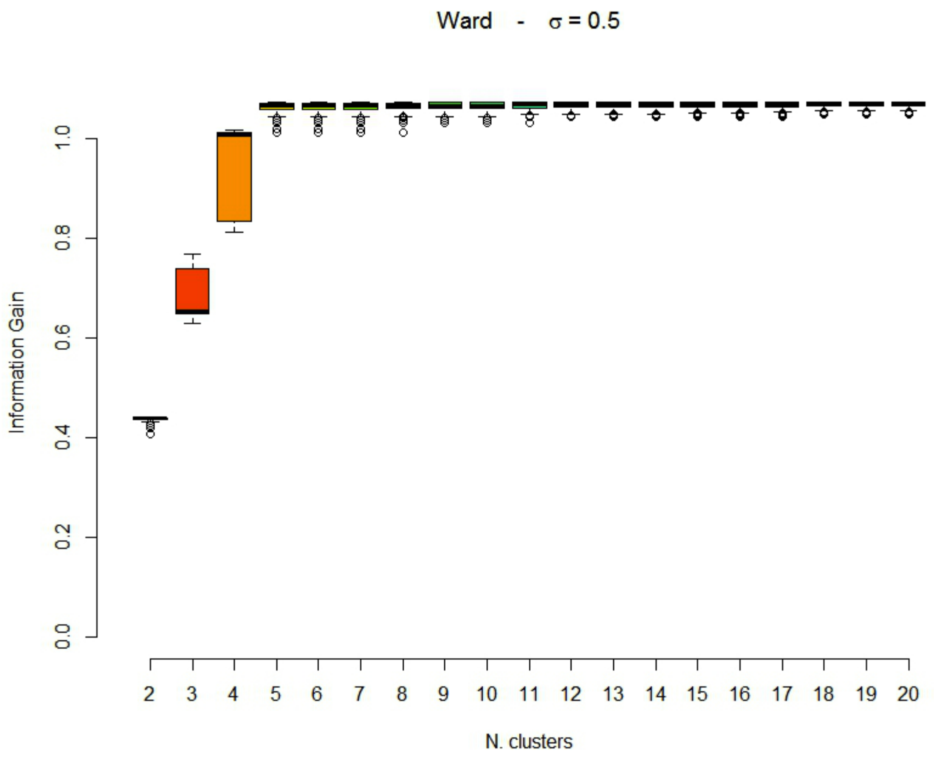

Figure S128. Boxplots of the information gain computed over Ward's results on 100 simulated datasets with $\sigma = 0.5$ for every number of clusters ranging between 2 and 20 are shown. Here at the beginning the information gain tends to increase when the number of clusters increases, showing an high variability of the the information gained by splitting the entire dataset in 2, 3, and 4 clusters. When approaching to the real number of clusters (6) the information gained by splitting the data is high, with a low variability of the results, meaning that clusters have a good quality in terms of impurity.

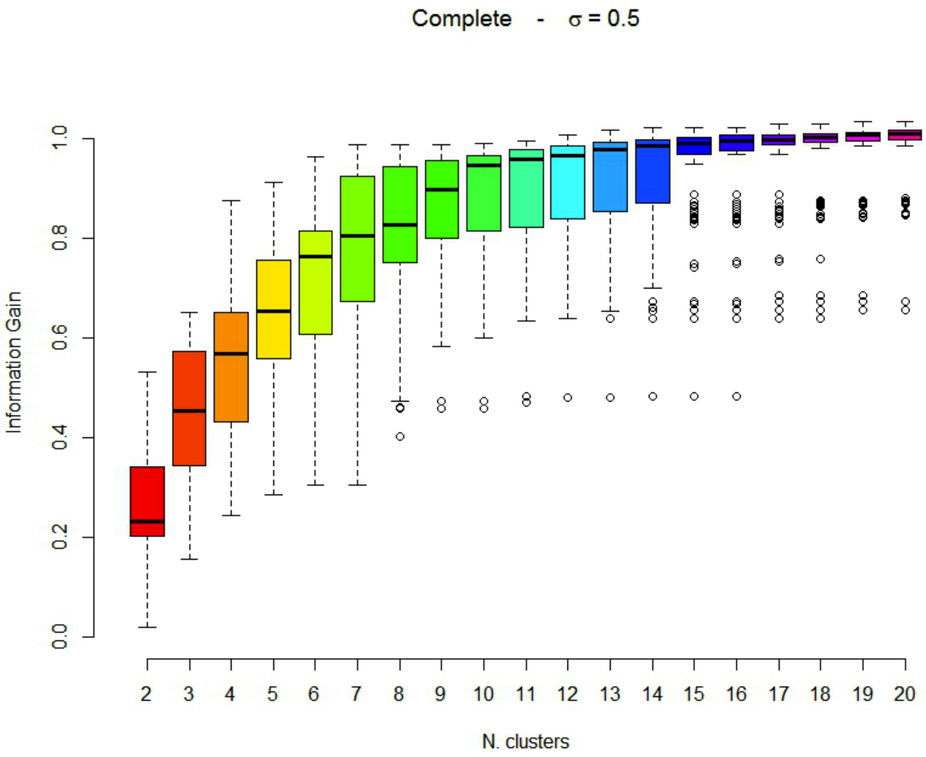

Figure S129. Boxplots of the information gain computed over CL results on 100 simulated datasets with $\sigma = 0.5$ for every number of clusters ranging between 2 and 20 are shown. Here at the beginning the information gain tends to increase when the number of clusters increases, showing an high variability of the results. The information gained by splitting the entire dataset in each value of the number of clusters tried is very variable and is not very high in correspondence with the real number of clusters (which is 6).

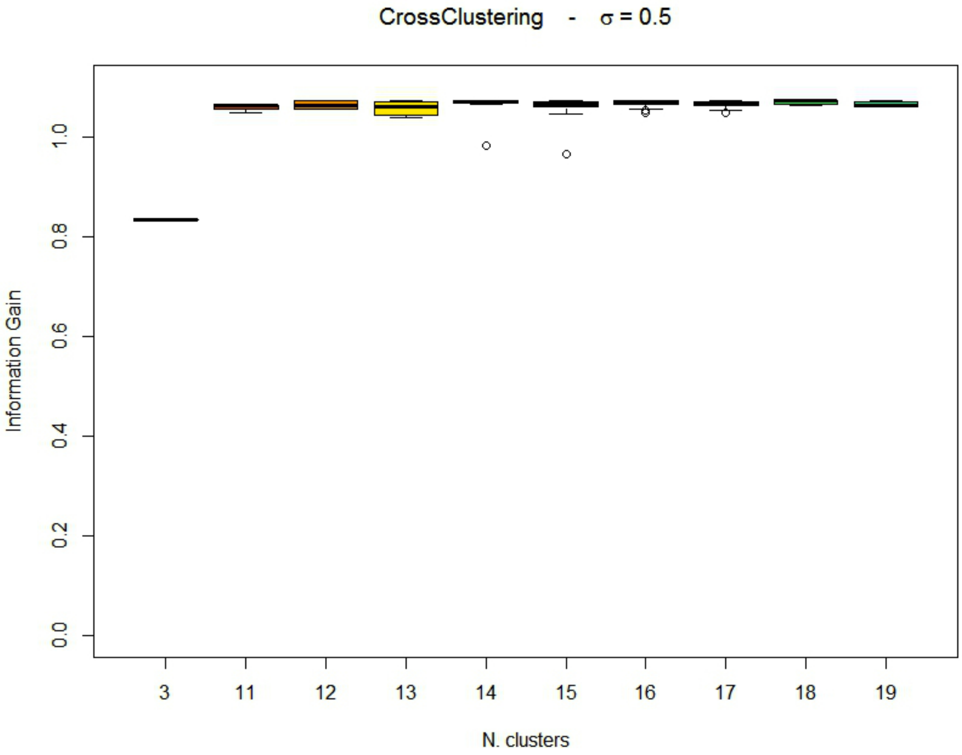

Figure S130. Boxplots of the information gain computed over CC results on 100 simulated datasets with $\sigma = 0.5$ for every number of clusters found by the algorithm are shown. The information gained by splitting the whole dataset in the number of clusters chosen by the algorithm is high, even if the real number of clusters (6) is never correctly identified.

References

1. Kaufman L, Rousseeuw P. Finding Groups in Data: An Introduction to Cluster Analysis; 1990.

2. R Core Team R: A Language and Environment for Statistical Computing. R Foundation for Statistical Computing 2015.

3. Xu R, Wunsch D. Survey of clustering algorithms. Neural Networks, IEEE Transactions on. 2005;16(3):645–678.
